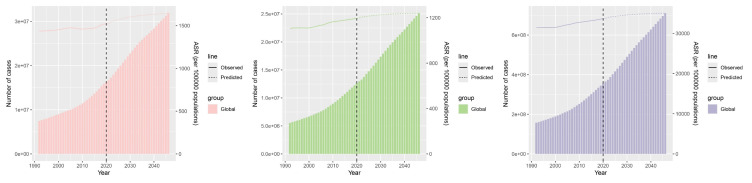# Global, regional, and national burden of osteoarthritis in elderly from 1990 to 2021: Insights from the global burden of disease study 2021

**DOI:** 10.1097/MD.0000000000048799

**Published:** 2026-05-15

**Authors:** Tianchen Zhang, Yinghong Li, Mingjie Tang, Zilei Xia, Yanhao Ge, Xinyi Hou, Yuyang Zhai, Shiwei Li, Tian Xiao, Tianwei Xia, Jirong Shen

**Affiliations:** a Department of Orthopedics, Affiliated Hospital of Nanjing University of Chinese Medicine, Nanjing, Jiangsu Province, China; b First Clinical Medical College, Nanjing University of Chinese Medicine, Jiangsu Province, Nanjing, China; c School of Medicine, Central South University, Changsha, Hunan Province, China.

**Keywords:** DALYs, elderly, GBD 2021, global burden of disease, osteoarthritis

## Abstract

Osteoarthritis (OA) in the elderly represents a critical public health challenge. This study utilized the Global Burden of Disease 2021 database to comprehensively assess the global burden of OA among individuals aged 60 and older from 1990 to 2021. We analyzed trends in disability-adjusted life years, prevalence, and incidence using the estimated annual percentage change. Additional methodologies included Joinpoint regression, decomposition analysis, and Nordpred predictive modeling to forecast epidemiological trajectories through 2046. In 2021, the estimated global number of prevalent OA cases among the elderly reached approximately 365.72 million, with a demographic distribution comprising 61.8% females and 38.2% males. Globally, the age-standardized rates per 100,000 population were 1197.55 for disability-adjusted life years, 33,751.01 for prevalence, and 1524.89 for incidence. From 1990 to 2021, the global OA burden exhibited a substantial and consistent rise. Geographically, the highest persistent burden occurred in high-income Asia Pacific, high-income North America, and regions with a high sociodemographic index. Stratified analyses identified the 60 to 64 age group and females as the populations bearing the greatest burden. Decomposition analysis revealed that population growth was the primary driver of the escalating OA burden globally. Furthermore, predictive modeling forecasts continued increases in these metrics by 2046. The escalating burden of OA among older adults is characterized by marked geographic and demographic disparities. These findings underscore the critical necessity for policymakers to implement targeted global public health strategies to mitigate the impact of this condition in aging populations.

## 1. Introduction

Osteoarthritis (OA) is a common chronic condition primarily impacting the elderly, significantly linked to increased mortality and disability rates, thus contributing to a substantial disease burden.^[1–4]^ The condition is marked by pain, deformity, and dysfunction, significantly affecting patients’ quality of life.^[5,6]^ Furthermore, it has been noted to markedly elevate the risk of detrimental cardiovascular incidents,^[7]^ lower extremity deep vein thromboembolism,^[8]^ and all-cause mortality.^[9]^ The increasing and aging global population is significantly elevating the prevalence of older patients with OA, presenting a substantial challenge to national healthcare systems.^[2]^ In 2020, the global prevalence of OA was estimated to be around 595 million individuals, representing 7.6% of the total population.^[10]^ The incidence of OA significantly contributes to years lived with disability (YLD), especially in individuals aged 70 and older.^[10]^ Moreover, substantial geographic disparities exist in the prevalence of OA.^[11]^ The 2021 data indicate that the age-standardized prevalence (ASPR) is greatest in the high-income Asia Pacific, high-income North America, and Australasia regions, whereas the lowest rates are found in Southeast Asia and Eastern Sub-Saharan Africa.^[11]^

The primary factor leading to the elevated incidence of OA in the elderly is age-associated joint degeneration, marked by the depletion of proteoglycans and diminished cartilage elasticity.^[12,13]^ Furthermore, the accumulation of prolonged mechanical stress (e.g., occupational strain injuries) and heightened joint loading resulting from obesity can expedite cartilage degradation.^[14–17]^ Moreover, the substantial decline in estrogen levels during menopause in middle-aged and elderly women accelerates cartilage degradation.^[18,19]^ Ultimately, diminished joint stability resulting from skeletal muscle atrophy and aberrant bone metabolism attributable to vitamin D deficiency converge to constitute the etiological factors of OA in the elderly.^[20,21]^ Contemporary management approaches for OA predominantly emphasize symptomatic alleviation rather than tackling the root causes, with the effectiveness and possible adverse effects of these strategies differing among individuals.^[1,22]^ The underdiagnosis and undertreatment of OA in older adults is frequently due to comorbidities and sociocognitive biases.^[2]^ Therefore, it is essential to investigate the impact of OA in older adults to formulate targeted interventions worldwide.

Prior research has concentrated on individuals of all ages,^[10,11,23]^ special populations,^[24,25]^ and particular risk factors,^[26]^ but there is a lack of research on the burden of OA in the globaland regional older populations. This study utilized the Global Burden of Disease (GBD) 2021 database to examine the disability-adjusted life years (DALYs), prevalence, and incidence of OA in individuals aged 60 and above from 1990 to 2021. Furthermore, we analyzed regional trends by clustering based on sociodemographic index (SDI) quintiles to evaluate the distribution and variations in the burden of OA in elderly worldwide. The findings of our study contribute to understanding the global burden of OA among the elderly while highlighting the need for potential strategies to support equitable healthcare policies, rational resource allocation, and the implementation of preventive measures worldwide.

## 2. Materials and methods

### 2.1. Overview, data sources, and definitions

The GBD 2021 study is a comprehensive epidemiological effort that quantifies health loss from over 300 diseases and injuries across 204 countries and territories.^[27]^ We extracted all raw data for this study at no cost from the Global Health Data Exchange query tool, which serves as the official public data catalog and repository maintained by the Institute for Health Metrics and Evaluation. In the GBD 2021 framework, the reference case definition for OA is symptomatic OA confirmed by radiological evidence, corresponding to Kellgren-Lawrence grades 2 through 4.^[28]^ Grade 2 indicates the presence of distinct osteophytes, grade 3 involves multiple osteophytes with joint space narrowing, and grade 4 adds severe bone deformity.^[29,30]^ Our study focused specifically on the elderly, defined here as individuals aged 60 and older. We categorized this population into 5-year age brackets (ranging from 60–64 up to 95+) for the period between 1990 and 2021. To assess the disease burden, we primarily evaluated DALYs, followed by prevalence and incidence. DALYs are standardly calculated as the sum of YLDs and years of life lost due to premature mortality. However, because OA is rarely a direct, underlying cause of death, the GBD framework assigns it a years of life lost value of zero. Consequently, the DALYs reported for OA in this study are driven entirely by YLDs, accurately reflecting the healthy life years lost to OA-related disability among the elderly. To explore socioeconomic disparities, we stratified our regional analysis using the SDI.^[31]^ The SDI is a composite indicator (ranging from 0 to 1) that evaluates a region based on per capita income, educational attainment, and fertility rates. We utilized the GBD’s 5-tier classification system: high (>0.81), high-middle (0.71–0.81), middle (0.62–0.71), low-middle (0.47–0.62), and low SDI (<0.47).

### 2.2. Statistical analyses

To mitigate the bias in the study results attributable to varying age structures, it is essential to calculate age-standardized rates using the following formula:



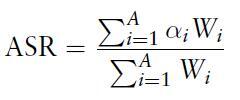



where *A* represents the total number of age groups, α_*i*_ denotes the age-specific rate per 100,000 population for the *i*th age group, and *W_i_* represents the standard population weight corresponding to the *i*th age group within the GBD reference population. The aim of this study was to evaluate the trends in DALYs, prevalence, and incidence among elderly OA patients. Consequently, to provide a standardized measure of the average annual rate of change, the estimated annual percentage changes (EAPC) and their 95% confidence intervals (CI) were computed using generalized linear regression models based on the following formula:^[32]^



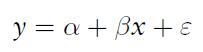





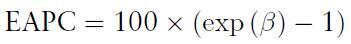



where *x* is the year, *y* is the natural logarithm of rates, α is the intercept, β is the slope, and ε is the random error. If the EAPC and its 95% CI are both above zero, the ASR shows an increasing trend. Conversely, if the EAPC and its 95% CI are both below zero, the ASR exhibits a decreasing trend. When the 95% CI includes zero, the change in ASR is not statistically significant. We also utilized Spearman correlation analysis to examine the relationship between the burden indicators of OA in the elderly and the SDI. Joinpoint regression analysis was employed to identify significant inflection points in the temporal trends of the OA burden.^[33]^ Allowing for a maximum of 5 joinpoints, we evaluated the trajectories of the age-standardized rates for disability-adjusted life years (ASDALYR), prevalence (ASPR), and incidence (ASIR) among the elderly from 1990 to 2021. The corresponding annual percentage changes (APC) and CI were subsequently calculated to quantify these shifts. To enhance the comprehensiveness of the assessment, we additionally calculated the average annual percentage change (AAPC) and CIs. The AAPC was determined utilizing the subsequent formula:^[34]^



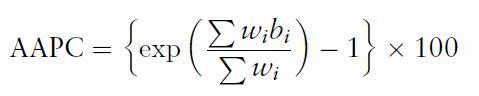



where *w*_*i*_ is the length of each year segment and *b*_*i*_ is the slope coefficient for each year segment. An APC/AAPC with a 95% CI lower bound >0 indicates an upward trend; if the upper bound <0, a downward trend. A 95% CI including zero suggests stability.

Decomposition analysis^[35]^ was employed to disaggregate the overall results into various factors and examine the contribution of each factor. Changes in the DALYs, prevalence, and incidence of OA were analyzed through 3 primary factors: demographic aging, population expansion, and epidemiological shifts.^[36]^ This enables us to comprehend how overarching trends in OA are shaped by the interplay of epidemiological and demographic factors in a multidimensional manner.

The Nordpred package in R software (R Foundation for Statistical Computing, Vienna, Austria)^[37]^ was utilized to project the trend of disease burden from OA in the elderly between 2021 and 2046. The model was developed using the age–period–cohort framework, which comprehensively accounts for demographic fluctuations, thereby offering a robust foundation for disease prediction.^[38]^ The R software version 4.4.1 was employed for data processing and visualization. A *P*-value threshold of <.05 indicates statistical significance. Information pertaining to the data sources and methodologies is available in the Supplementary Data.

## 3. Results

### 3.1. Global level

**Figure 1.**
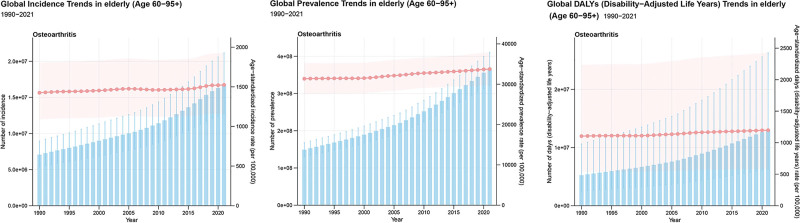
The trends in numbers and rates of incidence, prevalence, and DALYs for osteoarthritis in elderly in global from 1990 to 2021. DALYs = disability-adjusted life years.

In 2021, the global DALYs of OA in the elderly were 12,979,946 (95% uncertainty interval [UI]: 6,308,508–26,294,426), with an ASDALYR of 1197.55 (95% UI: 582.19–2425.53) per 100,000. The global prevalent cases were 365,716,375 (95% UI: 321,649,030–410,734,774), with an ASPR of 33,751.01 (95% UI: 29,707.93–37,886.37) per 100,000. The global incident numbers were 16,734,498 (95% UI: 12,745,585–21,167,115), corresponding to an ASIR of 1524.89 (95% UI: 1160.51–1929.26) per 100,000. From 1990 to 2021, the global ASDALYR (EAPC = 0.30, 95% CI: 0.27–0.32), ASPR (EAPC = 0.27, 95% CI: 0.24–0.29), and ASIR (EAPC = 0.17, 95% CI: 0.14–0.20) all showed an increasing trend, with the most pronounced increase observed in the ASDALYR (Tables 1–3; Fig. 1).

**Table 1:** The prevalence of osteoarthritis cases and rates in the elderly in 1990 and 2021, and the trends from 1990 to 2021.

	Prevalence				
Location	No., 1990 (95% UI)	ASPR, 1990 per 100,000 people (95% UI)	No., 2021 (95% UI)	ASPR, 2021 per 100,000 people (95% UI)	EAPC, 1990–2021 (95% CI)
Global	148,995,910 (130,926,112–167,680,414)	31,355.43 (27,617.54–35,239.11)	365,716,375 (321,649,030–410,734,774)	33,751.01 (29,707.93–37,886.37)	0.27 (0.24–0.29)
Low SDI	6,108,853 (5,337,100–6,957,396)	24,922.84 (21,867.89–28,308.19)	15,045,209 (13,167,736–17,038,263)	27,421.7 (24,075.5–30,989.29)	0.3 (0.28–0.32)
Low-middle SDI	17,281,564 (15,055,746–19,639,783)	25,789.17 (22,554.33–29,241.01)	49,290,610 (43,119,507–55,754,230)	29,434.31 (25,815.63–33,244.32)	0.42 (0.39–0.45)
Middle SDI	33,336,765 (29,135,892–37,793,264)	28,648.13 (25,138.3–32,396)	106,542,085 (93,421,482–120,147,566)	32,577.31 (28,615.58–36,692.49)	0.49 (0.45–0.52)
High-middle SDI	39,210,433 (34,345,859–44,135,139)	31,876.58 (27,992.98–35,840.58)	87,055,395 (76,388,331–97,746,374)	34,026.65 (29,871.7–38,189.4)	0.26 (0.23–0.3)
High SDI	52,887,311 (46,782,429–59,303,553)	36,579.6 (32,338.17–41,032.24)	107,456,129 (95,337,784–120,098,266)	38,751.6 (34,310.47–43,373.18)	0.18 (0.14–0.23)
Andean Latin America	754,822 (661,208–851,859)	32,230.87 (28,284.09–36,339.92)	2,556,594 (2,245,804–2,875,344)	35,635.38 (31,322.77–40,062.77)	0.32 (0.3–0.34)
Australasia	1,100,205 (972,479–1,232,428)	35,557.47 (31,429.21–39,829.44)	2,728,029 (2,420,097–3,041,348)	38,621.94 (34,209.07–43,102.81)	0.26 (0.24–0.28)
Caribbean	1,017,347 (891,626–1,148,183)	31,902.37 (27,979.82–35,988.34)	2,343,196 (2,062,644 to 2,632,023)	34,846.59 (30,669.04–39,144.26)	0.31 (0.3–0.32)
Central Asia	1,719,486 (1,485,147–1,971,418)	31,186.27 (26,996.21–35,703.47)	3,338,439 (2,875,295–3,833,969)	35,099.28 (30,355.3–40,158.21)	0.41 (0.37–0.46)
Central Europe	5,993,080 (5,223,580–6,791,957)	31,295.78 (27,330.3–35,417.87)	10,355,505 (9,058,297–11,695,982)	34,263.27 (29,940.98–38,712.56)	0.32 (0.3–0.33)
Central Latin America	3,089,004 (2,709,453–3,485,786)	32,651.11 (28,691.65–36,799.58)	11,178,561 (9,810,094–12,569,551)	36,399.71 (31,980.22–40,900.94)	0.37 (0.36–0.37)
Central Sub-Saharan Africa	654,958 (570,446–745,427)	27,627.35 (24,215.1–31,320.7)	1,619,203 (1,410,639–1,833,816)	29,279.49 (25,637.18–33,066.53)	0.15 (0.1–0.19)
East Asia	28,224,109 (24,540,726–32,149,192)	28,180.08 (24,596.67–32,008.86)	88,979,959 (77,998,021–100,861,663)	32,243.3 (28,283.35–36,498.6)	0.55 (0.47–0.63)
Eastern Europe	13,173,154 (11,417,776–14,991,785)	36,669.89 (31,860.81–41,659.9)	18,494,690 (16,082,739–20,993,177)	38,574.41 (33,574.73–43,748.04)	0.26 (0.23–0.29)
Eastern Sub-Saharan Africa	2,052,023 (1,793,592–2,332,479)	25,485.04 (22,371.73–28,880.73)	5,156,146 (4,505,119–5,830,425)	29,001.31 (25,436–32,727.97)	0.44 (0.43–0.45)
High-income Asia Pacific	10,257,237 (9,047,287–11,489,809)	40,807.94 (36,038.76–45,677.02)	25,821,860 (22,989,053–28,745,594)	42,652.09 (37,774.3–47,631.73)	0.34 (0.18–0.5)
High-income North America	18,138,957 (16,070,709–20,341,782)	38,874.16 (34,403.55–43,621.94)	36,127,677 (32,025,431–40,428,016)	40,744 (36,096.15–45,609.44)	−0.01 (−0.14 to 0.12)
North Africa and Middle East	4,913,226 (4,285,742–5,584,318)	26,208.56 (22,953.83–29,709.51)	15,468,167 (13,561,952–17,468,841)	30,281.16 (26,628.93–34,129.8)	0.45 (0.42–0.49)
Oceania	80,646 (70,533–91,721)	26,188.44 (23,031.56–29,673.92)	221,764 (194,297–251,330)	28,834.42 (25,389.09–32,585.67)	0.29 (0.28–0.31)
South Asia	16,074,817 (13,991,327–18,266,214)	25,989.17 (22,719.22–29,465.99)	52,673,365 (46,039,930–59,534,117)	30,250.33 (26,500.79–34,144.23)	0.48 (0.45–0.51)
Southeast Asia	6,416,040 (5,594,306–7,288,577)	22,900.54 (20,032.81–25,966.25)	20,896,497 (18,195,569–23,760,617)	27,080.44 (23,651.63–30,740.05)	0.56 (0.55–0.57)
Southern Latin America	2,024,672 (1,781,906–2,280,474)	34,537.65 (30,424.73–38,872.24)	4,236,527 (3,757,543–4,750,694)	37,532.23 (33,270.89–42,104.36)	0.26 (0.23–0.28)
Southern Sub-Saharan Africa	1,008,316 (880,389–1,140,551)	32,491.68 (28,434.42–36,697.86)	2,365,990 (2,062,734–2,673,062)	35,409.47 (30,957.74–39,937.85)	0.3 (0.29–0.31)
Tropical Latin America	3,451,143 (3,017,548–3,901,824)	32,827.38 (28,778.65–37,052.5)	11,734,378 (10,293,101–13,213,682)	36,630.53 (32,165.61–41,222.63)	0.37 (0.36–0.38)
Western Europe	26,177,433 (23,206,535–29,325,168)	34,085.92 (30,179.06–38,217.26)	43,194,889 (38,392,242–48,251,251)	35,588.1 (31,534.84–39,838.69)	0.13 (0.11–0.14)
Western Sub-Saharan Africa	2,675,236 (2,336,478–3,035,639)	27,185.66 (23,823.24–30,780.53)	6,224,940 (5,440,669–7,049,167)	29,824.4 (26,163.67–33,692.06)	0.31 (0.3–0.32)

ASPR = age-standardized prevalence rate, CI = confidence interval, EAPC = estimated annual percentage change, SDI = sociodemographic index, UI = uncertainty interval.

**Table 2:** The incidence of osteoarthritis cases and rates in the elderly in 1990 and 2021, and the trends from 1990 to 2021.

Location	Incidence				
	No., 1990 (95% UI)	ASIR, 1990 per 100,000 people (95% UI)	No., 2021 (95% UI)	ASIR, 2021 per 100,000 people (95% UI)	EAPC, 1990–2021 (95% CI)
Global	7,071,425 (5,407,513–8,942,103)	1425.2 (1088.01–1805.01)	16,734,498 (12,745,585–21,167,115)	1524.89 (1160.51–1929.26)	0.17 (0.14–0.2)
Low SDI	333,343 (255,815–421,432)	1266.04 (969.07–1604.29)	791,724 (607,721–997,707)	1361.85 (1043.02–1719.64)	0.23 (0.22–0.25)
Low-middle SDI	911,604 (695,995–1,154,254)	1280.06 (975.24–1623.86)	2,476,928 (1,890,911–3,133,973)	1416.28 (1079.39–1794.03)	0.33 (0.31–0.35)
Middle SDI	1,599,821 (1,208,412–2,042,282)	1305.86 (984.54–1669.09)	4,899,027 (3,703,755–6,248,275)	1460.17 (1102.46–1863.2)	0.34 (0.29–0.38)
High-middle SDI	1,825,859 (1,397,457–2,307,262)	1423.14 (1087.63–1801.49)	3,888,675 (2,958,489–4,925,320)	1510.37 (1148.12–1913.56)	0.13 (0.08–0.18)
High SDI	2,392,620 (1,845,028–3,008,332)	1669.93 (1287.98–2100.04)	4,663,327 (3,588,908–5,866,658)	1764.7 (1360.85–2214.58)	0.07 (0.04–0.1)
Andean Latin America	37,308 (28,607–47,137)	1545.48 (1184–1954.3)	120,892 (93,019–152,374)	1665.83 (1281.39–2100.42)	0.25 (0.25–0.26)
Australasia	50,154 (38,667–63,188)	1615.85 (1245.64–2036.45)	120,212 (92,298–152,274)	1775.63 (1366.13–2244.81)	0.24 (0.21–0.26)
Caribbean	49,387 (37,981 to 62,647)	1526.85 (1173.59–1938.11)	108,663 (83,265–137,230)	1622.48 (1243.76–2047.98)	0.23 (0.22–0.23)
Central Asia	75,203 (57,232–95,663)	1301.94 (991.51–1656.85)	140,537 (107,365–179,102)	1374.48 (1049.26 to 1750.23)	0.19 (0.17–0.21)
Central Europe	284,590 (217,727–359,352)	1439.05 (1099.03–1820.93)	454,438 (347,982–576,071)	1529.33 (1170.67 to 1938.59)	0.21 (0.2–0.21)
Central Latin America	154,384 (118,392 to 195,069)	1570.85 (1203.39–1987.29)	527,200 (403,817–666,060)	1681.17 (1287–2124.7)	0.22 (0.22–0.23)
Central Sub-Saharan Africa	34,678 (26,493–43,821)	1323.04 (1007.9–1675.32)	84,146 (64,702–106,071)	1383.51 (1059.12 to 1749.4)	0.12 (0.1–0.14)
East Asia	1,309,123 (974,601–1,698,736)	1253.63 (932.95–1626.04)	3,950,116 (2,957,316–5,073,342)	1416.52 (1058.72–1820.99)	0.33 (0.22–0.44)
Eastern Europe	580,203 (442,532–736,307)	1557.58 (1187.57–1977.98)	803,769 (614,831–1,021,544)	1651.42 (1262.41–2100)	0.21 (0.2–0.22)
Eastern Sub-Saharan Africa	111,665 (85,849–140,716)	1290.86 (989.57–1630.28)	268,024 (205,608–337,697)	1411.59 (1080.29–1782.22)	0.31 (0.3–0.31)
High-income Asia Pacific	506,958 (392,128–637,961)	1956.43 (1511.56–2466.12)	1,077,517 (827,272–1,360,704)	1996.02 (1540.87–2505.74)	0.04 (0.03–0.06)
High-income North America	761,569 (578,680–965,010)	1677.32 (1275.23–2123.37)	1,564,750 (1,197,518–1,974,863)	1793.27 (1373.1–2260.3)	−0.05 (−0.18 to 0.07)
North Africa and Middle East	257,310 (197,128–327,786)	1287.17 (983.74–1641.51)	765,145 (582,552–969,898)	1422.45 (1081.33–1804.77)	0.32 (0.31–0.33)
Oceania	3910 (2965–5001)	1178.03 (892.68–1508.83)	10,454 (7892–13,296)	1271.25 (958.94–1620.29)	0.23 (0.22–0.25)
South Asia	860,024 (656,071–1,091,044)	1298.61 (988.08–1651.11)	2,632,013 (2,004,441–3,343,036)	1446.15 (1099.14 to 1839.12)	0.33 (0.31–0.36)
Southeast Asia	316,638 (238,753–404,114)	1080.16 (813.82 to 1380.17)	986,206 (743,976–1,259,934)	1221.42 (920.77–1561.76)	0.42 (0.41–0.43)
Southern Latin America	94,724 (72,889–119,186)	1573.85 (1209.62–1983.07)	191,274 (147,158–240,708)	1721.83 (1325.61–2165.18)	0.21 (0.18–0.24)
Southern Sub-Saharan Africa	49,521 (37,859–62,787)	1525.26 (1163.93–1936.95)	115,675 (88,350–145,959)	1633.29 (1244.6–2066.74)	0.23 (0.22–0.23)
Tropical Latin America	178,672 (136,685 to 225,847)	1615.39 (1232.97–2045.4)	570,506 (436,908–722,293)	1747.39 (1337.3–2213.13)	0.27 (0.26–0.28)
Western Europe	1,216,465 (945,061–1,521,688)	1625.55 (1263.82–2031.97)	1,923,982 (1,493,658–2,412,506)	1720.94 (1341.19–2148.73)	0.12 (0.1–0.14)
Western Sub-Saharan Africa	138,937 (106,305–175,781)	1338.07 (1021.69–1696.11)	318,979 (243,888–404,200)	1435.2 (1095.34–1821.77)	0.23 (0.22–0.24)

ASIR = age-standardized incidence rate, CI = confidence interval, EAPC = estimated annual percentage change, SDI = sociodemographic index, UI = uncertainty interval.

**Table 3:** The DALYs and rates of osteoarthritis in the elderly in 1990 and 2021, and the trends from 1990 to 2021.

	DALYs				
Location	No., 1990 (95% UI)	ASDALYR, 1990 per 100,000 people (95% UI)	No., 2021 (95% UI)	ASDALYR, 2021 per 100,000 people (95% UI)	EAPC, 1990–2021 (95% CI)
Global	5,247,091 (2,557,428–10,620,802)	1103.55 (538.12–2233.87)	12,979,946 (6,308,508–26,294,426)	1197.55 (582.19–2425.53)	0.30 (0.27–0.32)
Low SDI	206,390 (100,235–414,827)	838.58 (408.02–1685.54)	515,299 (250,607–1,034,280)	936.86 (455.96–1881.07)	0.36 (0.34–0.38)
Low-middle SDI	586,575 (285,349–1,180,880)	872.83 (424.9–1757.96)	1,700,248 (825,682–3,436,774)	1013.57 (492.65–2048.5)	0.48 (0.45–0.51)
Middle SDI	1,150,423 (557,185–2,318,901)	986.41 (478.39–1988.43)	3,739,877 (1,812,553–7,562,171)	1142.2 (554.03–2308.64)	0.55 (0.52–0.59)
High-middle SDI	1,383,294 (672,416–2,810,211)	1124.05 (546.6–2283.74)	3,096,583 (1,498,922–6,292,220)	1210.01 (585.9–2458.38)	0.3 (0.26–0.34)
High SDI	1,914,388 (937,849–3,870,626)	1323.29 (648.48–2673.45)	3,916,309 (1,913,014–7,929,546)	1412.74 (690.23–2859.79)	0.22 (0.16–0.28)
Andean Latin America	26,796 (13,020–54,669)	1143.77 (555.84–2334.16)	91,680 (44,597–185,864)	1277.8 (621.6–2591.18)	0.36 (0.34–0.38)
Australasia	39,451 (19,256–79,194)	1273.85 (622.25–2555.01)	99,163 (48,727–200,558)	1404.53 (690.16–2838.54)	0.3 (0.28–0.32)
Caribbean	36,113 (17,625 to 73,486)	1131.56 (552.59–2301.19)	83,475 (40,559–169,364)	1241.58 (603.2–2518.84)	0.33 (0.32–0.34)
Central Asia	61,035 (29,596–123,047)	1107.76 (537.96–2235.33)	119,713 (57,840–240,060)	1260.03 (609.95–2531.04)	0.45 (0.4–0.5)
Central Europe	210,604 (102,910–427,556)	1099.24 (537.19–2232.44)	370,077 (180,827–752,581)	1224.1 (598.12–2487.57)	0.38 (0.37–0.4)
Central Latin America	108,358 (52,850–218,934)	1143.79 (558.12–2311.8)	399,328 (194,618–809,155)	1300.09 (633.53–2635.25)	0.43 (0.42–0.44)
Central Sub-Saharan Africa	22,523 (10,912–45,475)	945.6 (458.17–1913.3)	56,175 (27,057–113,750)	1013.73 (489.83–2055.19)	0.19 (0.14–0.24)
East Asia	970,105 (463,863–1,957,748)	966.29 (463.5–1949.7)	3,117,897 (1,491,069–6,309,601)	1128.46 (540.77–2281.89)	0.62 (0.54–0.71)
Eastern Europe	470,874 (228,671–960,318)	1311.62 (637.36–2676.27)	666,960 (324,001–1,352,645)	1391.54 (675.78–2823.22)	0.31 (0.27–0.35)
Eastern Sub-Saharan Africa	70,055 (34,095–141,065)	866.7 (422.42–1746.17)	179,275 (87,344–361,464)	1006.23 (490.32–2030.72)	0.52 (0.5–0.53)
High-income Asia Pacific	376,255 (183,071–762,502)	1496.31 (728.6–3031.23)	965,042 (467,649–1,960,327)	1592.71 (771.91–3232.96)	0.46 (0.25–0.66)
High-income North America	661,908 (324,537–1,341,422)	1417.75 (695.25–2870.04)	1,312,880 (646,512–2,652,307)	1480.78 (729.19–2990.73)	-0.04 (−0.2 to 0.11)
North Africa and Middle East	168,828 (81,841–341,095)	898.43 (435.82–1816.71)	536,534 (262,155–1,089,622)	1048.8 (512.23–2130.83)	0.49 (0.45–0.53)
Oceania	2766 (1340–5610)	893.97 (433.59–1812.49)	7663 (3701–15,417)	993.32 (480.57–1998.82)	0.33 (0.31–0.34)
South Asia	542,329 (263,910–1,092,416)	873.19 (425.22–1759.03)	1,818,003 (883,532–3,664,600)	1041.62 (506.75–2099.42)	0.57 (0.53–0.6)
Southeast Asia	218,802 (106,345–440,950)	778.14 (378.68–1567.71)	725,023 (351,275–1,462,957)	937.05 (454.46–1891.34)	0.63 (0.62–0.64)
Southern Latin America	72,770 (35,198–146,541)	1240.37 (600.32–2498.36)	153,535 (74,876–310,439)	1360.02 (663.42–2748.73)	0.29 (0.26–0.31)
Southern Sub-Saharan Africa	35,660 (17,393–72,303)	1147.57 (559.74–2327.72)	83,674 (41,000–168,640)	1251.36 (613.25–2525)	0.31 (0.3–0.32)
Tropical Latin America	120,410 (58,621–243,889)	1143.85 (557.19–2317.21)	416,080 (203,189–842,398)	1298.82 (634.29–2630.37)	0.43 (0.42–0.44)
Western Europe	939,666 (461,635–1,899,973)	1222.76 (600.88–2469.14)	1,560,798 (763,278–3,154,380)	1287.39 (629.9–2600.67)	0.15 (0.13–0.17)
Western Sub-Saharan Africa	91,781 (44,777–184,381)	930.89 (454.41–1870.75)	216,974 (105,810–437,098)	1038.15 (506.32–2094.07)	0.37 (0.35–0.39)

ASDALYR = age-standardized disability-adjusted life years rate, CI = confidence interval, DALYs = disability-adjusted life years, EAPC = estimated annual percentage change, SDI = sociodemographic index, UI = uncertainty interval.

### 3.2. SDI regional level

**Figure 2.**
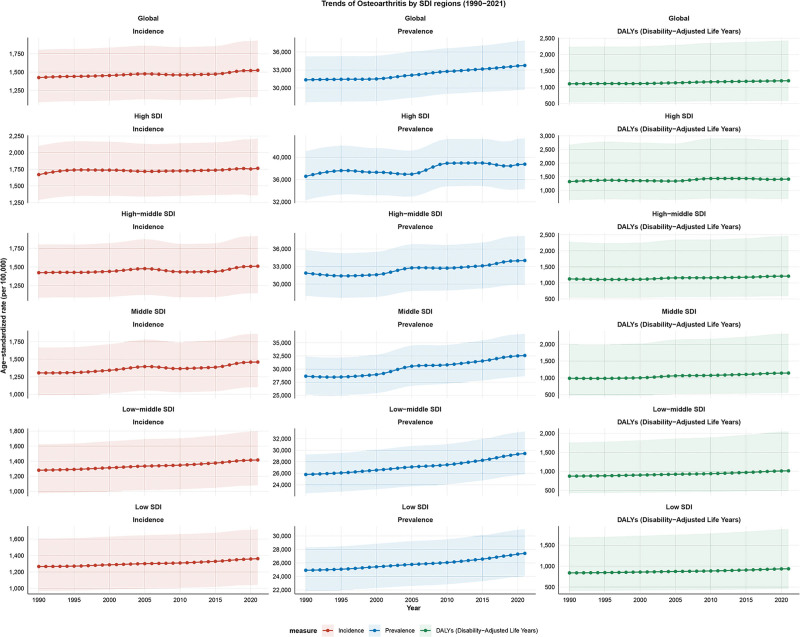
Trends of incidence, prevalence, and DALYs for osteoarthritis in elderly by SDI regions from 1990 to 2021. DALYs = disability-adjusted life years, SDI = sociodemographic index.

In 2021, across SDI regions, the absolute DALYs and prevalent cases of OA among older adults were highest in the high-SDI region, reaching 3,916,309 (95% UI: 1,913,014–7,929,546) and 107,456,129 (95% UI: 95,337,784–120,098,266), respectively. The highest incident cases were recorded in the middle SDI region, totaling 4,899,027 (95% UI: 3,703,755–6,248,275). Furthermore, the ASDALYR, ASPR, and ASIR among the elderly were also highest in the high-SDI region, recorded at 1412.74 (95% UI: 690.23–2859.79), 38,751.60 (95% UI: 34,310.47–43,373.18), and 1764.70 (95% UI: 1360.85–2214.58) per 100,000, respectively. From 1990 to 2021, the burden metrics increased across all SDI regions. The most pronounced increases in DALYs, prevalence, and incidence were observed in the middle SDI region, with EAPCs of 0.55 (95% CI: 0.52–0.59), 0.49 (95% CI: 0.45–0.52), and 0.34 (95% CI: 0.29–0.38), respectively (Tables 1–3; Fig. 2 and Fig. S1).

### 3.3. GBD regional level

The total number of DALYs, prevalence, and incidence of OA among older individuals have increased over time across all 21 global regions. In absolute terms, the primary GBD regions with the highest numbers of DALYs in 2021 were East Asia at 3,117,897 (95% UI: 1,491,069–6,309,601), South Asia at 1,818,003 (95% UI: 883,532–3,664,600), and Western Europe at 1,560,798 (95% UI: 763,278–3,154,380). Similarly, the primary GBD regions with a substantial number of prevalent cases were East Asia with 88,979,959 (95% UI: 77,998,021–100,861,663), South Asia with 52,673,365 (95% UI: 46,039,930–59,534,117), and Western Europe with 43,194,889 (95% UI: 38,392,242–48,251,251). The highest incident cases were also recorded in East Asia at 3,950,116 (95% UI: 2,957,316–5,073,342), South Asia at 2,632,013 (95% UI: 2,004,441–3,343,036), and Western Europe at 1,923,982 (95% UI: 1,493,658–2,412,506).

Regarding age-standardized rates, high-income Asia Pacific, high-income North America, and Australasia recorded the 3 highest ASDALYRs of 1592.71 (95% UI: 771.91–3232.96), 1480.78 (95% UI: 729.19–2990.73), and 1404.53 (95% UI: 690.16–2838.54) per 100,000, respectively. These regions also exhibited the highest ASPRs of 42,652.09 (95% UI: 37,774.3–47,631.73), 40,744 (95% UI: 36,096.15–45,609.44), and 38,621.94 (95% UI: 34,209.07–43,102.81) per 100,000, as well as the highest ASIRs of 1996.02 (95% UI: 1540.87–2505.74), 1793.27 (95% UI: 1373.10–2260.3), and 1775.63 (95% UI: 1366.13–2244.81) per 100,000.

**Figure 3.**
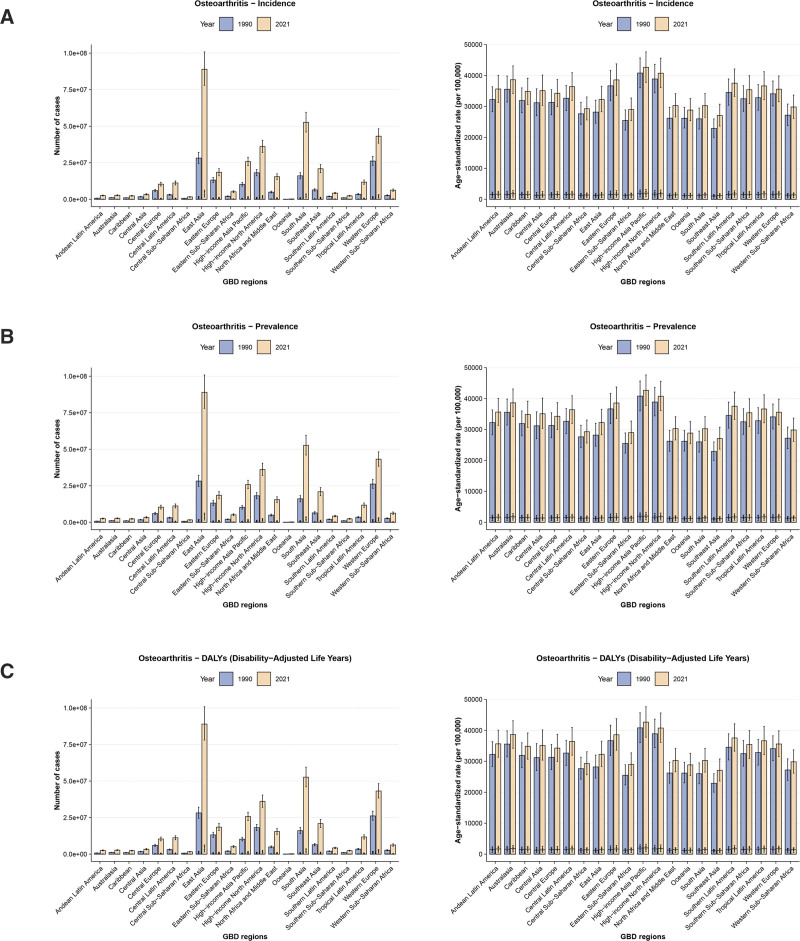
The numbers and rates of incidence (A), prevalence (B), and DALYs (C) for osteoarthritis in elderly by 21 regions in 1990 and 2021. DALYs = disability-adjusted life years, GBD = global burden of disease.

From 1990 to 2021, the burden metrics consistently increased throughout most regions. The most significant increases in DALYs and prevalence were observed in Southeast Asia and East Asia, exhibiting EAPCs of 0.56 (95% CI: 0.55–0.57) and 0.55 (95% CI: 0.47–0.63), respectively. Correspondingly, the most significant increases in incidence were recorded in Southeast Asia and South Asia, with EAPCs of 0.42 (95% CI: 0.41–0.43) and 0.33 (95% CI: 0.31–0.36), respectively (Tables 1–3; Fig. 3).

### 3.4. Countries level

**Figure 4.**
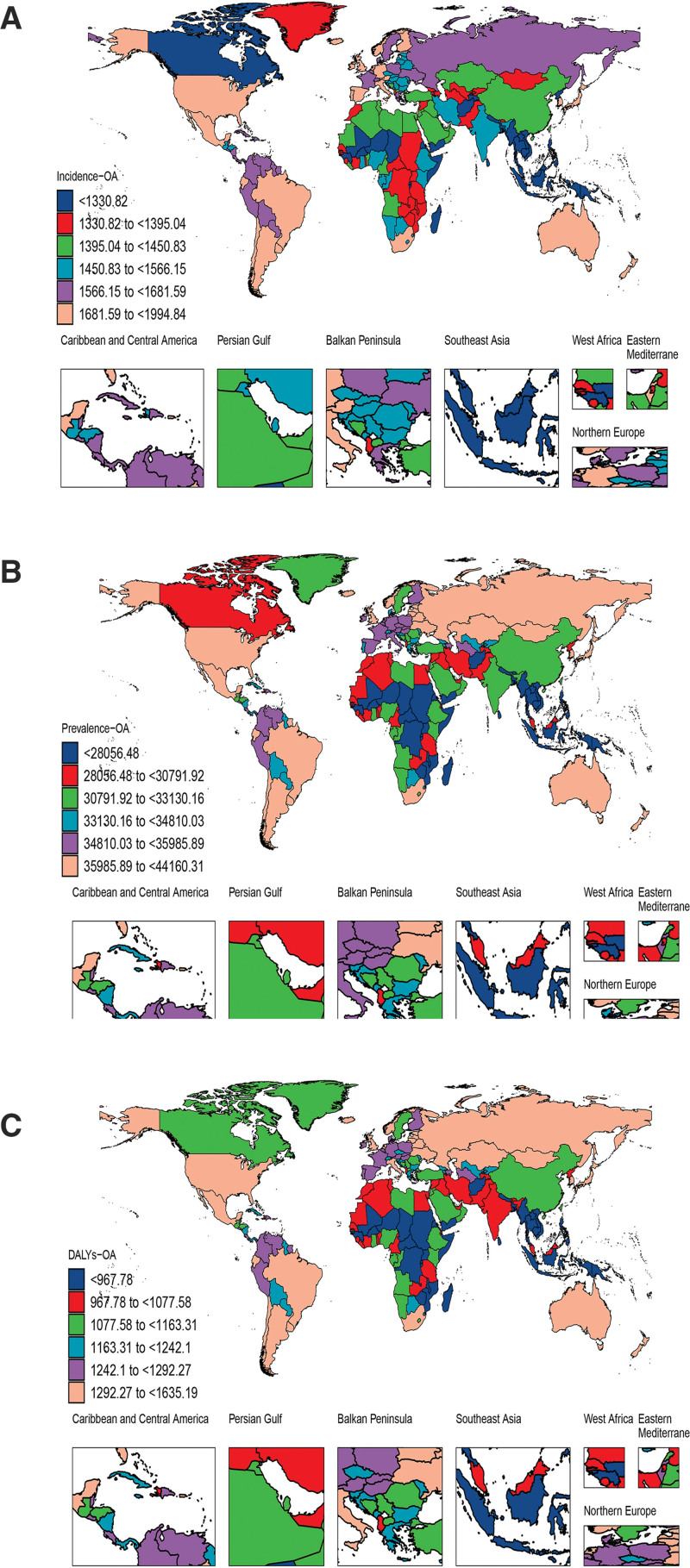
ASIR (A), ASPR (B), and ASDALYR (C) for osteoarthritis in elderly across 204 countries and territories in 2021. ASDALYR = age-standardized disability-adjusted life years rate, ASIR = age-standardized incidence rate, ASPR = age-standardized prevalence rate.

In 2021, at the national level, the countries with the highest absolute numbers of DALYs from OA among the elderly were China with 3,007,414 (95% UI: 1,438,250–6,086,673), India with 1,509,286 (95% UI: 734,304–3,041,514), the United States with 1,207,544 (95% UI: 595,095–2,437,772), and Japan with 743,152 (95% UI: 359,704–1,511,581). Regarding prevalent cases, China ranked first with 85,848,448 (95% UI: 75,251,382–97,323,326), followed by India with 43,708,648 (95% UI: 38,231,455–49,342,542), the United States with 33,160,430 (95% UI: 29,391,907–37,073,918), and Japan with 19,835,710 (95% UI: 17,698,651–22,071,836). The top 5 countries for incident cases were China at 3,818,742 (95% UI: 2,859,541–4,905,030), India at 2,178,794 (95% UI: 1,657,519–2,766,526), the United States at 1,439,798 (95% UI: 1,099,592–1,820,360), Japan at 801,509 (95% UI: 613,159–1,017,112), and the Russian Federation at 559,307 (95% UI: 427,476–711,172). In terms of age-standardized rates in 2021, the top 5 countries with the highest ASDALYRs were the Republic of Korea at 1635.19 (95% UI: 793.20–3316.89), Singapore at 1620.66 (95% UI: 788.54–3288.94), Brunei Darussalam at 1588.50 (95% UI: 768.53–3211.97), Japan at 1577.35 (95% UI: 762.74–3204.92), and the United States at 1529.34 (95% UI: 753.72–3086.66) per 100,000. For ASPR, the leading countries were the Republic of Korea, Brunei Darussalam, Singapore, Japan, and the United States, with rates of 44,160.31 (95% UI: 39,085.46–49,331.85), 43,193.38 (95% UI: 38,276.28–48,203.42), 43,100.11 (95% UI: 38,058.32–48,061.59), 42,153.12 (95% UI: 37,356.94–47,108.57), and 41,995.90 (95% UI: 37,203.28–46,968.84) per 100,000, respectively. For ASIR, the top 5 countries were Japan, the Republic of Korea, Brunei Darussalam, Singapore, and the United States, with rates of 1994.84 (95% UI: 1537.95–2513.41), 1986.50 (95% UI: 1524.32–2502.33), 1949.06 (95% UI: 1494.34–2461.73), 1942.81 (95% UI: 1491.03–2447.78), and 1850.65 (95% UI: 1413.95–2336.75) per 100,000, respectively. From 1990 to 2021, the DALYs, prevalence, and incidence of OA among the elderly increased in most countries. The largest increases in DALYs were observed in Equatorial Guinea and Ethiopia, with EAPCs of 1.27 (95% CI: 1.19–1.34) and 0.89 (95% CI: 0.85–0.93), respectively. For prevalence, the most substantial increases occurred in Equatorial Guinea and Mongolia, with EAPCs of 1.08 (95% CI: 1.02–1.15) and 0.81 (95% CI: 0.76–0.86), respectively. Correspondingly, the largest increases in incidence were recorded in Equatorial Guinea, Myanmar, and the Maldives, with EAPCs of 0.67 (95% CI: 0.63–0.71), 0.49 (95% CI: 0.47–0.51), and 0.49 (95% CI: 0.47–0.50), respectively (Fig. 4 and Tables S1–S3).

### 3.5. Age- and sex-specific subgroup analysis

**Figure 5.**
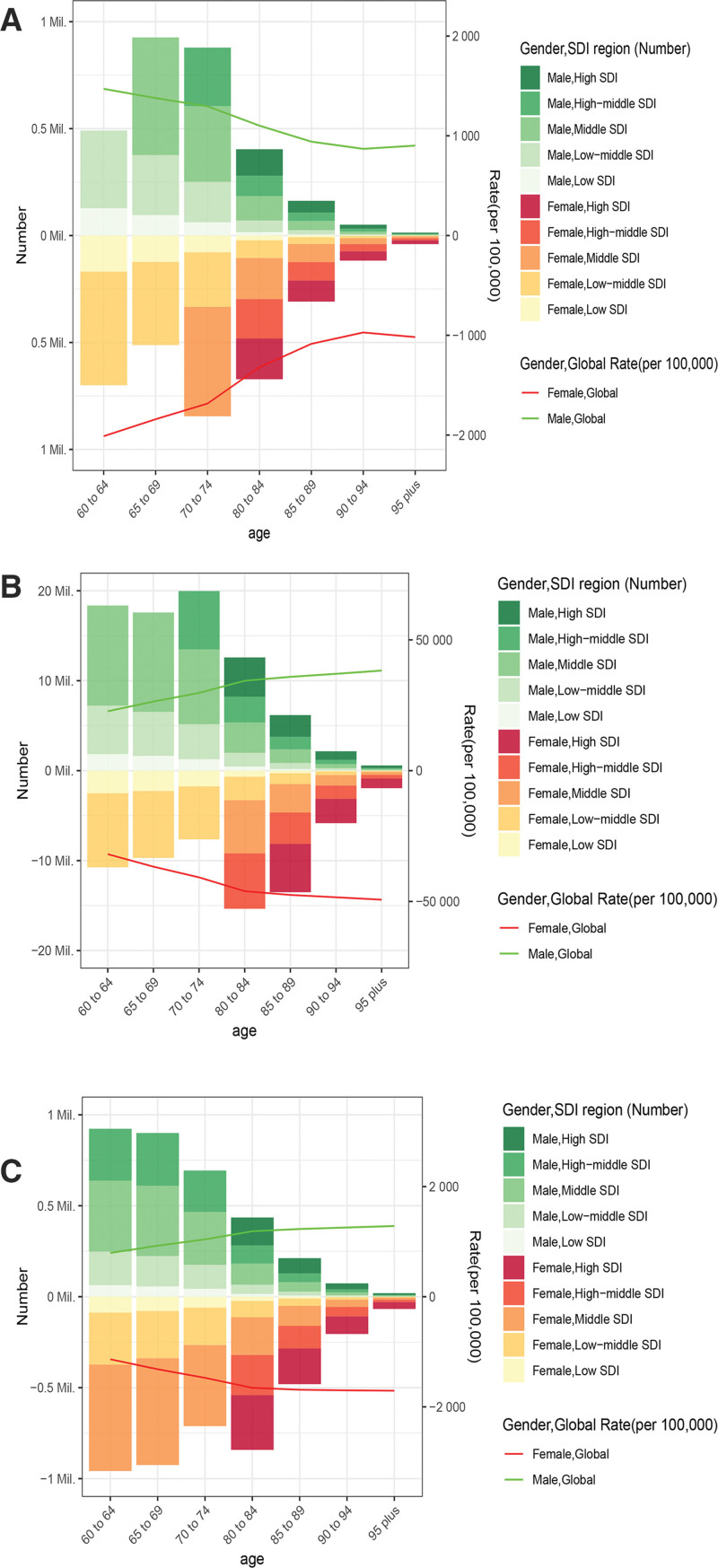
Gender and age-structure analysis of osteoarthritis burden in 2021. (A) Incidence, (B) prevalence, and (C) DALYs of osteoarthritis in the elderly by age group, sex, and SDI region. DALYs = disability-adjusted life years, SDI = sociodemographic Index.

In 2021, the DALY and prevalence rates of OA among the elderly showed similar trends, both increasing with age and peaking in the 95+ age group at 1589.68 (95% UI: 800.98–3153.20) and 46,251.93 (95% UI: 41,750.31–51,266.32) per 100,000, respectively. In contrast, the incidence rate generally decreased with age, reaching its lowest level in the 90 to 94 age group before showing a slight increase in the 95+ age group. Regarding absolute burden, the number of DALYs showed an initial increase followed by a decrease, peaking in the 65 to 69 age group at 3,118,800.93 (95% UI: 1,529,705.91–6,348,047.71). Conversely, the prevalent and incident cases of OA among the elderly both exhibited a declining trend with age. Across all elderly age groups, the absolute numbers of DALYs, prevalent cases, and incident cases were consistently higher in women compared with men. Specifically, the numbers of DALYs and prevalent cases in elderly women both peaked in the 65 to 69 age group, reaching 1,896,428 (95% UI: 929,123.4–3,856,585) and 52,833,382 (95% UI: 46,031,879–59,652,228), respectively. Meanwhile, the number of incident cases was highest in the 60 to 64 age group, reaching 3,309,153 (95% UI: 2,518,618–4,140,816). Similarly, the age-specific rates of DALYs, prevalence, and incidence in 2021 were higher in women than in men across all age brackets. Overall, the global burden of OA among elderly women was consistently higher than that among men (Fig. 5 and Fig. S2).

### 3.6. The association between ASR and SDI

**Figure 6.**
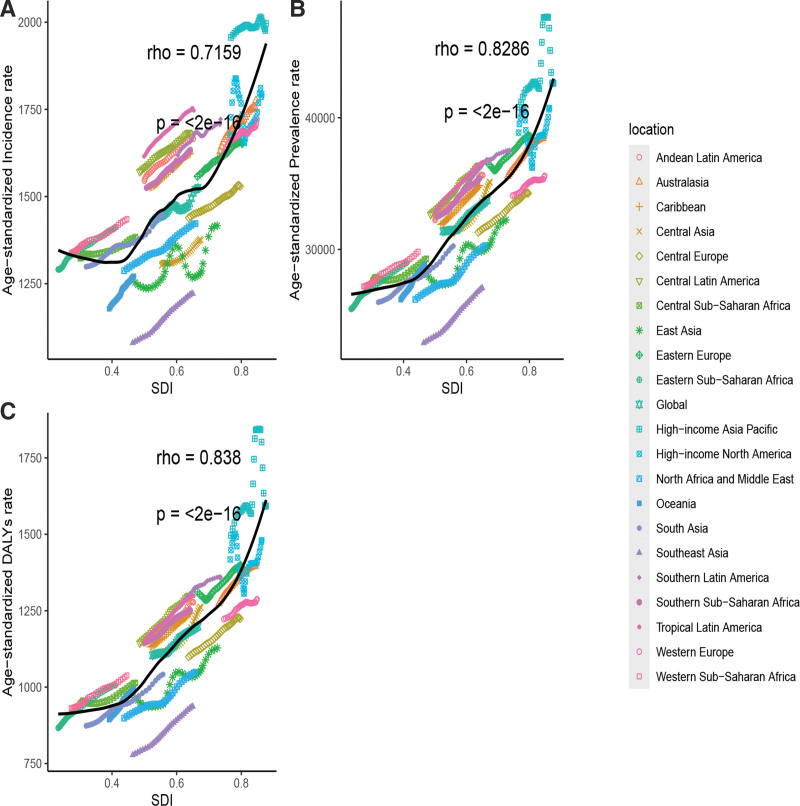
The associations between the SDI and ASIR (A), ASPR (B), and ASDALYR (C) for osteoarthritis in elderly across 21 regions. ASDALYR = age-standardized disability-adjusted life years rate, ASIR = age-standardized incidence rate, ASPR = age-standardized prevalence rate, DALYs = disability-adjusted life years, SDI = sociodemographic index.

From 1990 to 2021, the SDI across the 21 GBD regions exhibited a significant positive correlation with the ASDALYR, ASPR, and ASIR of OA in the elderly. These age-standardized rates generally increased with increasing SDI, yielding correlation coefficients of 0.838, 0.8286, and 0.7159, respectively. High-income Asia Pacific reported higher ASDALYRs, ASPRs, and ASIRs compared with other regions, whereas Southeast Asia exhibited lower rates relative to its SDI. Furthermore, the trends in ASDALYR and ASPR for OA in the elderly were consistent across the 21 GBD regions. In recent years, the ASDALYR, ASPR, and ASIR experienced a slight decline within high-income Asia Pacific (Fig. 6).

### 3.7. Decomposition analysis

From 1990 to 2021, decomposition analysis indicated an overall increase in the OA burden among the elderly across DALYs, prevalence, and incidence. The increase in these OA metrics worldwide and across the 5 SDI regions was primarily driven by population growth, followed by epidemiological shifts. Over the past 3 decades, global population growth accounted for increases of 88.64%, 89.44%, and 93.45% in the DALYs, prevalence, and incidence of OA among the elderly, respectively, with growth rates generally higher in women than in men. In the high-SDI region, population growth contributed to 88.76%, 89.64%, and 95.42% of the increases in DALYs, prevalence, and incidence, respectively. The contribution of population aging to OA incidence was negative across all 5 SDI regions, reaching −4.02% in the high-SDI region (Fig. S3).

### 3.8. Joinpoint regression analysis

Joinpoint regression analysis indicated that the ASDALYR (AAPC = 0.26%, 95% CI: 0.26%–0.27%, *P* < .001), ASPR (AAPC = 0.24%, 95% CI: 0.23%–0.24%, *P* < .001), and ASIR (AAPC = 0.21%, 95% CI: 0.20%–0.22%, *P* < .001) of OA in the elderly demonstrated an upward trajectory, with inflection points identified for all 3 metrics in the year 2000. Furthermore, inflection points were identified for the ASDALYR (APC = 0.26%, 95% CI: 0.26%–0.27%, *P* < .001) and ASPR (APC = 0.25%, 95% CI: 0.21%–0.27%, *P* < .001) in 2009. During specific intervals, reductions were observed: the ASDALYR (APC = −0.02%, 95% CI: −0.04% to 0.00%, *P* < .001) decreased from 1994 to 2000, and the ASIR (APC = −0.24%, 95% CI: −0.32% to −0.18%, *P* < .001) decreased from 2005 to 2010 (Fig. S4).

### 3.9. Prediction analysis

The worldwide epidemiological burden of OA among the elderly is projected to increase through 2046. By 2046, the estimated number of DALYs is projected at 25,152,804 (females: 15,114,797; males: 10,038,007). The global prevalent cases are anticipated to reach 710,698,727 (females: 425,341,523; males: 285,357,204), and the incident cases are projected at 31,891,889 (females: 18,701,918; males: 13,189,971).

Regarding age-standardized rates, the global ASDALYR for OA among the elderly is estimated to be 1241.12 (females: 1380.32; males: 1080.84) per 100,000 by 2046. The ASPR is projected at 35,051.24 (females: 38,835.75; males: 30,708.95) per 100,000, and the ASIR is anticipated to be 1647.57 (females: 1827.66; males: 1451.66) per 100,000.

In comparison with 2021, the global ASDALYR, ASPR, and ASIR in 2046 are projected to increase, with higher projected growth in males than in females. Furthermore, the projections indicate a decelerating rate of increase for these epidemiological metrics over the forecast period (Fig. S5).

## 4. Discussion

Our analysis delineates a consistently escalating global burden of OA among the elderly from 1990 to 2021. Specifically, ASDALYR, ASPR, and ASIR all maintained an upward trajectory. This burden exhibited pronounced regional heterogeneity, demonstrating a strong positive correlation with regional SDI levels. Demographically, while the incidence of OA generally declined with advancing age, the rates of DALYs and prevalence steadily increased. Furthermore, elderly women bore a substantially heavier disease burden across all evaluated metrics compared with men. Projection models suggest this global expansion will continue through 2046, characterized by rising ASDALYR, ASPR, and ASIR, alongside a steeper projected growth trajectory among males.

As a prominent cause of functional impairment, OA ranks 18th among the 369 conditions evaluated in the GBD study.^[39]^ The widespread prevalence and clinical complexity of the disease impose substantial demands on global healthcare systems.^[40]^ While several recent epidemiological assessments have utilized GBD 2021 data to explore OA trends,^[10,23,25]^ these investigations have typically encompassed broader age groups or pursued different research objectives. By focusing exclusively on the elderly – the demographic disproportionately affected by OA – our analysis provides a targeted evaluation of historical burden and future epidemiological trajectories. The pronounced burden observed in elderly women aligns with established literature, a disparity frequently attributed to postmenopausal estrogen depletion and distinct lifestyle variables.^[10,41]^

These findings indicate a positive correlation between the global burden of OA in the elderly and the SDI. In regions with elevated SDI levels, the prevalence of OA among the elderly is notably significant, likely due to the aging population and increasing obesity rates in these areas.^[42]^ Related studies indicate that the ASIR of OA correlates with the SDI and geographical location, presumably due to variations in socioeconomic situations, regional characteristics, and healthcare service availability.^[43]^ The significant prevalence of OA among the elderly is evident not only in wealthy nations such as Singapore and the United States but also in developing countries like Brunei Darussalam. In 2021, China and India recorded the greatest new cases of OA among the aged worldwide; however, their ASDALYR, ASPR, and ASIR did not attain the highest levels. This gap may be attributed to the substantial population base of these 2 nations. In the last 32 years, the prevalence of OA among the elderly has markedly risen in less developed nations, including Equatorial Guinea and Ethiopia, a trend potentially attributable to inadequate healthcare resources, and a deficiency of effective prevention and management strategies for OA in these areas.^[44]^ In affluent Asia Pacific nations (e.g., Japan, Republic of Korea), the ASDALYR, ASPR, and ASIR of OA among the elderly persist at elevated levels. This phenomenon may be linked to accelerated population aging,^[45]^ enhanced healthcare systems resulting in increased disease detection rates, and conventional lifestyle factors^[46,47]^ (e.g., sitting postures that elevate mechanical stress on the knees). In recent years, high-income Asia Pacific countries, which face a considerable burden of OA, have exhibited a downward trend in ASDALYR, ASPR, and ASIR, largely attributable to enhancements in healthcare resources and the advocacy of preventative measures.^[48]^

Prior research indicates that the elevated incidence of OA in the elderly is influenced by factors including age, obesity, joint traumas, metabolic disorders, osteoporosis, and chronic inflammation.^[48–51]^ Aging, elevated body mass index, and joint traumas are frequently regarded as substantial factors in the development of OA. The aging process results in diminished chondrocyte functionality and extracellular matrix formation, hence impairing cartilage healing capabilities and heightening vulnerability to mechanical stress.^[49]^ Simultaneously, persistent low-grade inflammation intensifies cartilage deterioration via the activation of pro-inflammatory cytokines.^[52]^ Obesity, a prevalent condition in the elderly, not only heightens mechanical stress on weight-bearing joints but also induces systemic inflammation via adipokine release, hence exacerbating the course of OA.^[53]^ Furthermore, previous joint injuries or surgeries, prevalent among the elderly, impair joint biomechanics and may result in secondary OA.^[54]^ Metabolic disorders, including diabetes and dyslipidemia, contribute to the etiology of OA by producing oxidative stress and disrupting cartilage homeostasis.^[55]^ The significant prevalence of OA in the elderly is attributable to both age-related degenerative processes and external risk factors, highlighting the necessity for increased focus on its impact.

This study also found a greater burden of OA in women among older OA patients. This difference may be due to the drop in estrogen levels in postmenopausal women^[41]^ and the stronger muscle and ligament tissue in men, resulting in stronger joint support. Joinpoint regression analysis revealed that the ASDALYR, ASPR, and ASIR of OA among the elderly exhibited an overall upward trend, with inflection points observed for all 3 metrics in the year 2000. This may be associated with the accelerated global aging process and updates to the diagnostic criteria for OA.^[44,56,57]^ In addition, inflection points for ASDALYR and ASPR were also observed in 2009, which may be linked to the reduction in healthcare resources following the global economic crisis of 2008.^[58]^

Currently, there is no definitive cure for OA.^[11]^ Consequently, early prevention of OA is critically important. Regular physical activity is acknowledged as a fundamental strategy for the clinical treatment and management of OA patients. Research indicates that moderate exercise can significantly enhance the synthesis of cartilage glycosaminoglycans, an effect that is especially pronounced in populations at elevated risk for OA.^[59,60]^ Moreover, efforts ought to address modifiable factors such as tobacco use, obesity, and joint problems.^[42,61]^ Research examining risk factors linked to OA has found obesity as the predominant risk factor.^[62,63]^ Consequently, the most endorsed preventative strategies involve decreasing body mass index and preventing prolonged chronic joint stress, which should be prioritized in worldwide treatment, management, and prevention initiatives.^[23]^ Moreover, health education for the elderly, especially older women, must be enhanced, and both national and global intervention plans should be formulated to effectively mitigate the disease burden of OA.

Recognizing the escalating burden of OA, several high-prevalence nations and international organizations have established targeted public health frameworks. National action plans from the United States and Australia, alongside updated European guidelines, consistently emphasize preventive lifestyle modifications such as weight management and structured exercise. These initiatives also highlight the necessity of expanding healthcare coverage for surgical and pharmaceutical interventions.^[64–67]^ Translating these multifaceted strategies to a global scale will be critical to counteracting the projected surge in OA-related DALYs and prevalence among the aging demographic.

This research has several limitations. The GBD 2021 study has limited data, particularly from low- and middle-income countries, which may be attributed to underdeveloped healthcare systems, weak research infrastructure, and insufficient data collection capabilities in these regions. This may result in possible biases in assessing the burden of OA in the aged population. This study also failed to consider potential contributing factors, including lifestyle and illness awareness. Moreover, this study did not examine the prevalence of OA at various anatomical locations individually. This research focused solely on the nonfatal burden of OA in the elderly and did not explore its association with excess mortality. This research did not examine the potential correlation between increased mortality in elderly OA patients and factors such as limited mobility, comorbidities, and chronic pain, nor did it consider shared risk factors and confounding effects with other fatal diseases.^[68]^ Obesity and metabolic syndrome are not just risk factors for OA but also substantial contributors to cardiovascular diseases and diabetes, potentially affecting health outcomes in the aged through intricate pathways.

Subsequent research ought to focus on bridging these gaps by integrating additional data from low- and middle-income nations, assessing the influence of lifestyle and socioeconomic determinants on OA in the elderly, and investigating the correlation between OA and mortality within this demographic. These findings will inform early therapies for OA in the elderly, thereby alleviating the global burden of this condition in this group.

## 5. Conclusion

In conclusion, this study showed the increasing trends in ASDALYR, ASPR, and ASIR and their underlying causes in older people with OA worldwide from 1990 to 2021, highlighting the importance of geographical, sex, and age factors. We also projected that ASDALYR, ASPR, and ASIR will continue to increase in older adults with OA worldwide over the next 25 years, and that the burden will continue to be greater in older women than in men. There is an urgent need for governments to develop targeted interventions to further reduce the burden of OA in the elderly in all regions and countries worldwide.

## Acknowledgments

We extend our appreciation to the Institute for Health Metrics and Evaluation for granting access to the essential GBD data.

## Author contributions

**Conceptualization:** Tianchen Zhang, Yinghong Li, Mingjie Tang, Zilei Xia, Yanhao Ge, Tianwei Xia, Jirong Shen.

**Data curation:** Yinghong Li, Mingjie Tang, Yanhao Ge, Xinyi Hou, Yuyang Zhai, Shiwei Li, Tian Xiao, Jirong Shen.

**Software:** Yinghong Li, Xinyi Hou.

**Methodology:** Shiwei Li, Jirong Shen.

**Writing – original draft:** Tianchen Zhang, Mingjie Tang, Tianwei Xia, Jirong Shen.

**Writing – review & editing:** Tianchen Zhang, Mingjie Tang, Jirong Shen.







**Figure:**
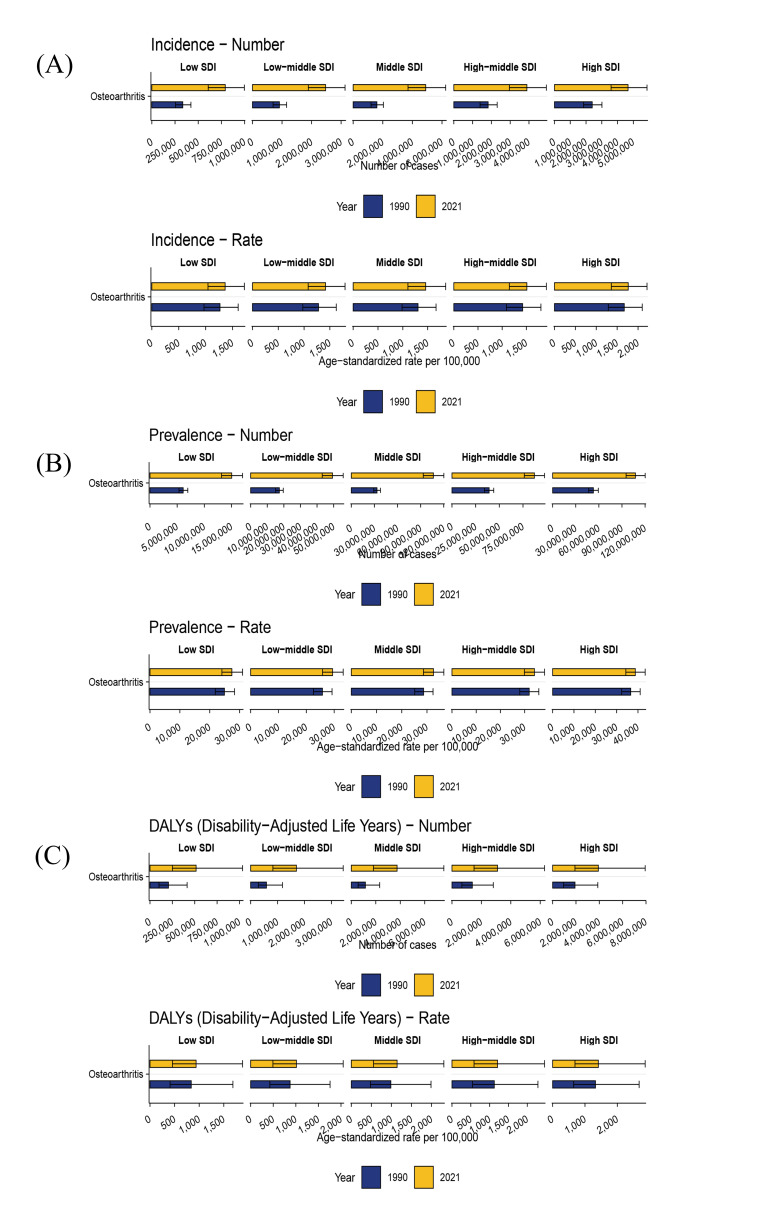


**Figure:**
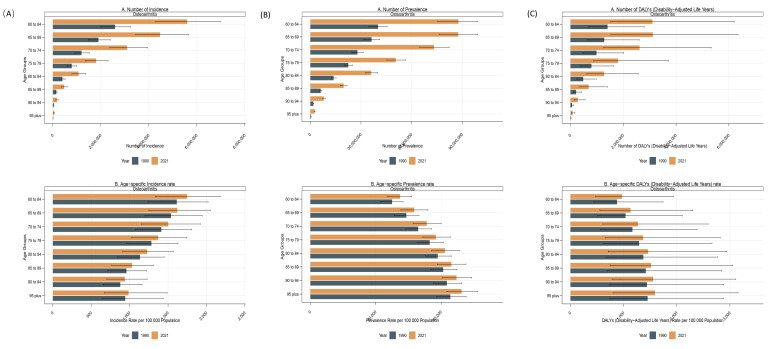


**Figure:**
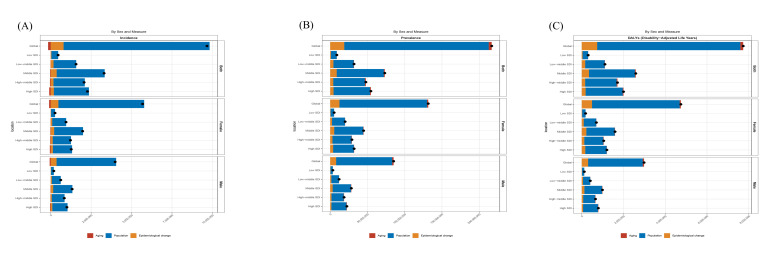


**Figure:**
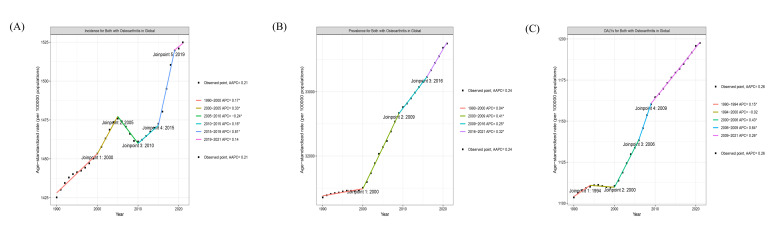


**Figure:**