# Supplementary material for: Global, regional, and national burden of osteoarthritis in elderly from 1990 to 2021: Insights from the global burden of disease study 2021
Source: Medicine (Baltimore). 2026 May 15;105(20):e48799. doi: 10.1097/MD.0000000000048799 (PMC13183078; doi:10.1097/MD.0000000000048799)
Supplement: Supplementary file 1 [file medi-105-e48799-s002.doc]

**Supplementary Table S1** The prevalence of osteoarthritis cases and rates in the elderly population across 204 countries and territories in 1990 and 2021, and the trends from 1990 to 2021.

| Country | incidence | | | | |
| --- | --- | --- | --- | --- | --- |
|  | No.,1990 (95% UI) | ASIR, 1990 per 100,000 people (95% UI) | No.,2021, (95% UI) | ASIR, 2021 per 100,000 people (95% UI) | EAPC,1990-2021, (95% CI) |
| Afghanistan | 180753 (157358 to 207251) | 22325.61 (19518.92 to 25516.54) | 202907 (177086 to 230665) | 25034.93 (21855.35 to 28452.31) | 0.43 (0.38 to 0.48) |
| Albania | 59363 (52118 to 67234) | 25250.98 (22220.93 to 28552.1) | 170122 (148827 to 192287) | 28674.71 (25106.31 to 32389.55) | 0.49 (0.46 to 0.51) |
| Algeria | 377480 (328500 to 430116) | 26061.15 (22741.28 to 29621.57) | 1281346 (1118922 to 1449766) | 30689.93 (26863.06 to 34660.71) | 0.54 (0.52 to 0.55) |
| American Samoa | 793 (692 to 895) | 33020.08 (28956.88 to 37150.82) | 1966 (1721 to 2214) | 35361.52 (31059.33 to 39754.78) | 0.18 (0.14 to 0.22) |
| Andorra | 2412 (2126 to 2713) | 32969.36 (29096.29 to 37044.33) | 6798 (6021 to 7641) | 35040.05 (31017.54 to 39400.08) | 0.2 (0.18 to 0.22) |
| Angola | 115826 (100292 to 132850) | 30037.57 (26185.93 to 34293.24) | 387684 (335610 to 440116) | 32838.46 (28585.13 to 37121.96) | 0.29 (0.29 to 0.3) |
| Antigua and Barbuda | 2368 (2079 to 2663) | 33770.22 (29593.3 to 38032.99) | 4713 (4132 to 5330) | 35822.26 (31477.29 to 40450.63) | 0.18 (0.17 to 0.19) |
| Argentina | 1433440 (1256898 to 1613999) | 34707.07 (30465.14 to 39047.26) | 2717731 (2408648 to 3055922) | 37511.56 (33222.11 to 42207.84) | 0.24 (0.21 to 0.27) |
| Armenia | 91578 (79238 to 105144) | 28695.31 (24936.06 to 32855.38) | 203002 (175066 to 233341) | 34254.56 (29595.84 to 39319.83) | 0.68 (0.63 to 0.73) |
| Australia | 913458 (807553 to 1022074) | 35452.32 (31341.48 to 39665.97) | 2294089 (2034171 to 2559261) | 38577.52 (34149.09 to 43088.5) | 0.27 (0.25 to 0.28) |
| Austria | 533126 (472570 to 596536) | 33736.7 (29861.2 to 37791.48) | 821599 (730326 to 920531) | 35102.04 (31128.45 to 39413.82) | 0.11 (0.1 to 0.12) |
| Azerbaijan | 179757 (154360 to 207368) | 32359.54 (27865.12 to 37271.92) | 416267 (356904 to 480329) | 35025.36 (30215.37 to 40179.03) | 0.34 (0.25 to 0.43) |
| Bahamas | 6113 (5358 to 6907) | 35038.15 (30736.83 to 39564.73) | 17742 (15446 to 20010) | 36804.08 (32154.91 to 41433.18) | 0.16 (0.14 to 0.17) |
| Bahrain | 5073 (4402 to 5737) | 30255.35 (26451.47 to 34119) | 29883 (25949 to 33891) | 32452.54 (28451.78 to 36578.45) | 0.22 (0.21 to 0.23) |
| Bangladesh | 1254921 (1092205 to 1430203) | 24045.23 (20973.16 to 27365.79) | 4535123 (3961005 to 5159881) | 27226.56 (23811.75 to 30941.91) | 0.43 (0.4 to 0.46) |
| Barbados | 13574 (11939 to 15325) | 34645.92 (30369.77 to 39198.25) | 25562 (22364 to 28766) | 36817.88 (32228.57 to 41419.67) | 0.2 (0.18 to 0.22) |
| Belarus | 559631 (486691 to 635345) | 33471.92 (29163.47 to 37950.1) | 808578 (704639 to 916818) | 37118.29 (32390.3 to 42056.22) | 0.38 (0.37 to 0.4) |
| Belgium | 688842 (608333 to 775007) | 33574.7 (29624.32 to 37789.74) | 1069387 (946968 to 1195318) | 35037.68 (30958.68 to 39227.52) | 0.12 (0.11 to 0.13) |
| Belize | 3581 (3138 to 4046) | 31502.43 (27609.53 to 35583.6) | 11987 (10521 to 13507) | 35445.85 (31185.38 to 39883.52) | 0.35 (0.31 to 0.4) |
| Benin | 60129 (52516 to 68191) | 25994.59 (22739.78 to 29444.18) | 168050 (147510 to 190432) | 30403.83 (26748.82 to 34397.81) | 0.51 (0.48 to 0.53) |
| Bermuda | 2824 (2489 to 3178) | 36480.13 (32175.46 to 41020.65) | 6913 (6077 to 7750) | 37860.61 (33236.01 to 42476.59) | 0.11 (0.1 to 0.12) |
| Bhutan | 6156 (5360 to 7032) | 24690.21 (21587.36 to 28108.22) | 20097 (17627 to 22768) | 28004.38 (24586.8 to 31701.15) | 0.42 (0.4 to 0.43) |
| Bolivia (Plurinational State of) | 110306 (96438 to 125395) | 30438.79 (26682.85 to 34521.63) | 374880 (328976 to 421654) | 34321.58 (30190.62 to 38547.13) | 0.39 (0.37 to 0.4) |
| Bosnia and Herzegovina | 128355 (111487 to 145523) | 27646.69 (24119.13 to 31269.07) | 271158 (236306 to 307707) | 32091.59 (27972.4 to 36404.53) | 0.55 (0.48 to 0.61) |
| Botswana | 17944 (15660 to 20418) | 28663.72 (25129.94 to 32507.43) | 53997 (47307 to 61174) | 33782.59 (29703.76 to 38155.78) | 0.5 (0.48 to 0.52) |
| Brazil | 3365763 (2942419 to 3805939) | 32832.85 (28780.15 to 37064.56) | 11493884 (10082509 to 12944205) | 36684.14 (32213.35 to 41287.55) | 0.37 (0.37 to 0.38) |
| Brunei Darussalam | 4253 (3748 to 4773) | 40426.94 (35690.78 to 45304.54) | 16561 (14600 to 18558) | 43193.38 (38276.28 to 48203.42) | 0.21 (0.2 to 0.22) |
| Bulgaria | 514665 (448290 to 584576) | 31362.18 (27378.1 to 35541.45) | 672107 (587200 to 762758) | 34297.45 (29918.73 to 38961.17) | 0.3 (0.29 to 0.31) |
| Burkina Faso | 119046 (104036 to 136700) | 24138.93 (21166.7 to 27627.58) | 267559 (232876 to 305611) | 26313.77 (22984.09 to 30003.14) | 0.27 (0.27 to 0.28) |
| Burundi | 64186 (55910 to 73080) | 24674.88 (21554.47 to 28027.75) | 128943 (112570 to 147067) | 25237.16 (22148.99 to 28670.45) | 0.09 (0.08 to 0.1) |
| Cabo Verde | 7770 (6800 to 8846) | 26165.29 (22870.5 to 29818.06) | 16316 (14262 to 18503) | 31174.7 (27329.61 to 35320.98) | 0.6 (0.59 to 0.61) |
| Cambodia | 100319 (87211 to 114962) | 20688.47 (18053.88 to 23627.78) | 338640 (296904 to 385664) | 24035.02 (21141.32 to 27313.57) | 0.53 (0.51 to 0.55) |
| Cameroon | 132355 (115704 to 150604) | 27255.17 (23933.79 to 30920.72) | 404581 (354850 to 458937) | 30517.28 (26869.89 to 34526.34) | 0.33 (0.31 to 0.36) |
| Canada | 1201896 (1042352 to 1369919) | 28329.74 (24567.26 to 32289.73) | 2964064 (2590946 to 3371595) | 30528.61 (26658.34 to 34749.13) | 0.13 (0.07 to 0.19) |
| Central African Republic | 29762 (25836 to 33736) | 25951 (22678.15 to 29313.27) | 55798 (48527 to 63638) | 26952.95 (23596.51 to 30607.22) | 0.11 (0.1 to 0.11) |
| Chad | 79799 (69608 to 90946) | 24209.05 (21148.46 to 27552.85) | 155098 (135555 to 177482) | 25427.2 (22297.55 to 29009.09) | 0.15 (0.14 to 0.15) |
| Chile | 412222 (363680 to 464917) | 33963.12 (29994.32 to 38272.5) | 1246841 (1104086 to 1397543) | 37579.79 (33280.54 to 42121.97) | 0.31 (0.28 to 0.34) |
| China | 27163861 (23614632 to 30955661) | 28160.11 (24574.02 to 31999.75) | 85848448 (75251382 to 97323326) | 32233.69 (28273.68 to 36491.33) | 0.55 (0.47 to 0.63) |
| Colombia | 622737 (544299 to 705931) | 31319.42 (27444.98 to 35436.42) | 2431570 (2135499 to 2733912) | 34957.97 (30712.97 to 39297.01) | 0.38 (0.37 to 0.39) |
| Comoros | 5445 (4746 to 6233) | 25276.84 (22126.82 to 28832.87) | 15398 (13458 to 17449) | 28268.77 (24770.35 to 31980.87) | 0.39 (0.37 to 0.41) |
| Congo | 34683 (30150 to 39359) | 29540.54 (25781.17 to 33423.41) | 86597 (75484 to 98272) | 32163.39 (28151.79 to 36396.84) | 0.26 (0.24 to 0.28) |
| Cook Islands | 452 (397 to 509) | 31454.84 (27729.65 to 35388.18) | 1204 (1061 to 1356) | 35540.42 (31353.76 to 40032.76) | 0.37 (0.34 to 0.41) |
| Costa Rica | 65881 (57931 to 74545) | 31753.73 (27941.18 to 35912.06) | 245651 (215319 to 277499) | 35281.65 (30948.53 to 39835.16) | 0.34 (0.33 to 0.35) |
| Coted'Ivoire | 103579 (90199 to 118125) | 26288.56 (23025.88 to 29849.14) | 331862 (289878 to 377780) | 29192.59 (25612.11 to 33130.47) | 0.31 (0.29 to 0.32) |
| Croatia | 241146 (209701 to 273452) | 31545.03 (27523.73 to 35706.97) | 408375 (357169 to 461815) | 33678.27 (29407.29 to 38129.92) | 0.26 (0.24 to 0.28) |
| Cuba | 406166 (355924 to 459428) | 31546.69 (27632.7 to 35687.79) | 852249 (750231 to 957064) | 34674.58 (30484.86 to 38967.17) | 0.33 (0.32 to 0.34) |
| Cyprus | 33780 (29804 to 38073) | 31605.03 (27918.71 to 35591.25) | 93916 (83251 to 105557) | 34213.22 (30313.54 to 38463.16) | 0.26 (0.24 to 0.29) |
| Czechia | 603840 (523677 to 687713) | 33067.61 (28690.02 to 37644.4) | 1001845 (873503 to 1132511) | 34827.43 (30284.39 to 39412.41) | 0.16 (0.14 to 0.18) |
| Democratic People's Republic of Korea | 482711 (421695 to 550281) | 27563.61 (24156.15 to 31332.05) | 1168246 (1020586 to 1322050) | 29710.51 (25980.38 to 33594.67) | 0.24 (0.23 to 0.25) |
| Democratic Republic of the Congo | 450436 (393133 to 513594) | 27026.3 (23727.66 to 30674.34) | 1034669 (900820 to 1173510) | 27919.84 (24436.1 to 31576.94) | 0.03 (-0.02 to 0.09) |
| Denmark | 383691 (340013 to 431090) | 35747.54 (31607.4 to 40223.64) | 541299 (479394 to 606300) | 34460.32 (30432.73 to 38679.64) | -0.06 (-0.09 to -0.03) |
| Djibouti | 3230 (2809 to 3693) | 25361 (22184.29 to 28864.3) | 18853 (16429 to 21416) | 29790.47 (26105.36 to 33730.87) | 0.61 (0.57 to 0.64) |
| Dominica | 2486 (2178 to 2809) | 31998.38 (28028.31 to 36162.1) | 3692 (3236 to 4152) | 34801.26 (30564.39 to 39101.25) | 0.26 (0.22 to 0.3) |
| Dominican Republic | 135574 (118751 to 153016) | 31795.71 (27901.42 to 35849.54) | 425365 (372614 to 480139) | 35231.73 (30877.69 to 39757.46) | 0.35 (0.34 to 0.36) |
| Ecuador | 200779 (175795 to 227095) | 32958.95 (28895.77 to 37250.48) | 731836 (643498 to 825176) | 36278.49 (31910.08 to 40892.04) | 0.33 (0.31 to 0.34) |
| Egypt | 749990 (649692 to 858944) | 26165.95 (22802.17 to 29819.03) | 2080533 (1825877 to 2363160) | 29657.36 (26170.63 to 33550.78) | 0.32 (0.28 to 0.35) |
| El Salvador | 109908 (96153 to 124375) | 31214.08 (27328.81 to 35309.18) | 270964 (238621 to 305453) | 34986.17 (30770.68 to 39470) | 0.38 (0.36 to 0.41) |
| Equatorial Guinea | 5099 (4440 to 5823) | 25030.52 (21909.98 to 28483.43) | 16863 (14705 to 19048) | 33127.29 (29004.5 to 37334.57) | 1.08 (1.02 to 1.15) |
| Eritrea | 23427 (20361 to 26725) | 24134.8 (21123.14 to 27417.46) | 71224 (61928 to 80979) | 26634.05 (23282.45 to 30191.6) | 0.32 (0.31 to 0.33) |
| Estonia | 92430 (80616 to 105000) | 34865.83 (30437.63 to 39584.59) | 138930 (121308 to 156911) | 38341.51 (33382.62 to 43403.84) | 0.32 (0.29 to 0.35) |
| Eswatini | 8734 (7643 to 9929) | 29112.09 (25567.39 to 32999.99) | 20178 (17621 to 22893) | 33432.41 (29334.31 to 37798.13) | 0.4 (0.36 to 0.45) |
| Ethiopia | 537703 (470065 to 611932) | 25960.65 (22797.34 to 29430.69) | 1487569 (1293811 to 1693719) | 31707.69 (27638.4 to 36034.47) | 0.74 (0.71 to 0.78) |
| Fiji | 10434 (9125 to 11874) | 29238.8 (25661.09 to 33165.77) | 30020 (26232 to 34082) | 33887.24 (29743.89 to 38342.76) | 0.46 (0.43 to 0.5) |
| Finland | 317500 (281258 to 355001) | 33682.96 (29817.42 to 37672.71) | 599019 (532192 to 672256) | 35222.74 (31200.2 to 39624.33) | 0.14 (0.12 to 0.15) |
| France | 3672995 (3253454 to 4120572) | 33448.28 (29575.71 to 37566.91) | 6435926 (5724833 to 7195337) | 35051.91 (31086.53 to 39285.75) | 0.14 (0.12 to 0.17) |
| Gabon | 19152 (16705 to 21788) | 28231.83 (24691.68 to 32052.13) | 37592 (32781 to 42389) | 32704.31 (28655.01 to 36793.6) | 0.45 (0.42 to 0.48) |
| Gambia | 9852 (8571 to 11214) | 26292.25 (22954.51 to 29848.49) | 32398 (28254 to 36593) | 30494.17 (26658.41 to 34398.59) | 0.48 (0.47 to 0.49) |
| Georgia | 243597 (210138 to 279411) | 30879.54 (26702.55 to 35372.02) | 270047 (234138 to 308898) | 33133.02 (28726.56 to 37906.86) | 0.19 (0.12 to 0.25) |
| Germany | 5679485 (5018702 to 6350468) | 34378.53 (30323.25 to 38466.65) | 8968015 (7962221 to 10062078) | 35415.1 (31337.21 to 39837.46) | 0.05 (0.04 to 0.07) |
| Ghana | 204018 (177537 to 232047) | 30509.99 (26658.2 to 34590.55) | 580835 (506940 to 656159) | 31970.43 (28027.17 to 36028.69) | 0.2 (0.1 to 0.3) |
| Greece | 632872 (558968 to 715307) | 31609.6 (27921.07 to 35727.68) | 1059469 (943108 to 1185275) | 34205.28 (30334.88 to 38382.05) | 0.55 (0.43 to 0.67) |
| Greenland | 974 (844 to 1118) | 28670.01 (24968.03 to 32735.15) | 2616 (2279 to 2976) | 30865.45 (27022.16 to 34973.63) | 0.23 (0.21 to 0.25) |
| Grenada | 3045 (2672 to 3437) | 31626.66 (27697.6 to 35739.32) | 4753 (4169 to 5368) | 34955.12 (30724.48 to 39417.82) | 0.32 (0.28 to 0.36) |
| Guam | 2679 (2334 to 3031) | 31784.01 (27849.53 to 35848.9) | 9677 (8489 to 10899) | 35124.34 (30831.65 to 39538.7) | 0.33 (0.31 to 0.35) |
| Guatemala | 111449 (97097 to 126659) | 29119.37 (25476.1 to 33010.96) | 426664 (373757 to 481401) | 32262.76 (28292.77 to 36374.55) | 0.33 (0.32 to 0.33) |
| Guinea | 98128 (85673 to 112372) | 24649.03 (21569.66 to 28163.14) | 167721 (146155 to 191638) | 26685.54 (23321.63 to 30414.46) | 0.23 (0.22 to 0.24) |
| Guinea-Bissau | 10796 (9395 to 12325) | 25194.58 (22028.39 to 28652.96) | 19910 (17370 to 22574) | 27546.7 (24169.07 to 31150.91) | 0.27 (0.26 to 0.28) |
| Guyana | 13379 (11685 to 15123) | 31122.47 (27251.25 to 35128.02) | 26293 (22880 to 29705) | 34672.29 (30304.27 to 39069.95) | 0.35 (0.33 to 0.37) |
| Haiti | 96843 (84411 to 111149) | 27117.93 (23734.52 to 30997.09) | 228197 (198933 to 259409) | 29521.94 (25843.56 to 33465.81) | 0.3 (0.29 to 0.31) |
| Honduras | 68639 (59846 to 77994) | 29756.74 (26004.46 to 33762.5) | 242396 (212345 to 275058) | 32779.41 (28777.9 to 37132.76) | 0.32 (0.31 to 0.32) |
| Hungary | 641551 (560835 to 727603) | 32931.18 (28809.63 to 37327.79) | 936428 (817868 to 1062204) | 35216.87 (30710.01 to 40004.2) | 0.19 (0.15 to 0.22) |
| Iceland | 13324 (11815 to 14977) | 35758.71 (31676.55 to 40219.04) | 27637 (24599 to 30912) | 36087.04 (32094.35 to 40397.1) | -0.01 (-0.07 to 0.05) |
| India | 13031821 (11346600 to 14813301) | 26535.32 (23214.63 to 30082.08) | 43708648 (38231455 to 49342542) | 30852.98 (27045.98 to 34783.6) | 0.47 (0.43 to 0.51) |
| Indonesia | 2447174 (2132183 to 2786958) | 23579.42 (20618.11 to 26786.63) | 7447170 (6467999 to 8494395) | 27738.61 (24195.63 to 31536.99) | 0.53 (0.51 to 0.55) |
| Iran (Islamic Republic of) | 786997 (684951 to 894740) | 27121.75 (23738.43 to 30713.9) | 2764152 (2410854 to 3126523) | 30708.97 (26849.5 to 34690.49) | 0.42 (0.35 to 0.49) |
| Iraq | 249257 (216921 to 282689) | 27825.53 (24245.03 to 31540.44) | 765232 (668599 to 868353) | 30017.75 (26316.8 to 33962.17) | 0.2 (0.18 to 0.22) |
| Ireland | 179429 (158775 to 201950) | 33034.23 (29214.47 to 37190.84) | 360688 (320675 to 405045) | 35034.57 (31119.93 to 39387.35) | 0.18 (0.16 to 0.2) |
| Israel | 211588 (186626 to 238023) | 33055.22 (29142.9 to 37193.09) | 566588 (503442 to 634692) | 35142.75 (31184.3 to 39396.14) | -0.28 (-0.47 to -0.09) |
| Italy | 4078507 (3602598 to 4581196) | 34365.84 (30337.5 to 38610.16) | 6731228 (5982563 to 7521868) | 35875.75 (31758.47 to 40190.99) | 0.22 (0.15 to 0.28) |
| Jamaica | 73815 (64800 to 83218) | 31781.83 (27869.1 to 35851.6) | 135122 (118883 to 152207) | 34577.44 (30426.66 to 38951.05) | 0.28 (0.26 to 0.31) |
| Japan | 8752250 (7725498 to 9811188) | 40441.99 (35726.51 to 45313.02) | 19835710 (17698651 to 22071836) | 42153.12 (37356.94 to 47108.57) | 0.36 (0.18 to 0.54) |
| Jordan | 38189 (33284 to 43405) | 28328.62 (24798.73 to 32100.77) | 251675 (220393 to 284799) | 31908.67 (28051.52 to 36024.77) | 0.39 (0.38 to 0.41) |
| Kazakhstan | 494512 (423984 to 568542) | 33157.75 (28524.59 to 38031.44) | 827468 (712440 to 952033) | 38639.04 (33396.32 to 44322.88) | 0.53 (0.49 to 0.58) |
| Kenya | 254457 (222148 to 288364) | 28030.03 (24547.41 to 31704.81) | 813054 (707785 to 922817) | 32725.56 (28593.15 to 37033.24) | 0.52 (0.5 to 0.54) |
| Kiribati | 1198 (1046 to 1357) | 30222.59 (26495.16 to 34108.18) | 2555 (2228 to 2889) | 32787.55 (28779.67 to 36935.77) | 0.21 (0.17 to 0.25) |
| Kuwait | 16654 (14577 to 18891) | 29892.6 (26273.83 to 33796.55) | 89740 (78681 to 101574) | 32587.36 (28688.19 to 36801.19) | 0.34 (0.32 to 0.36) |
| Kyrgyzstan | 112049 (96415 to 129196) | 31228.44 (26939.39 to 35933.66) | 186739 (160179 to 216150) | 34397.29 (29639.4 to 39650.54) | 0.4 (0.32 to 0.48) |
| Lao People's Democratic Republic | 48027 (41983 to 54811) | 21069.44 (18472.57 to 23980.74) | 122274 (106440 to 138856) | 24321.95 (21249.5 to 27569.91) | 0.5 (0.48 to 0.53) |
| Latvia | 158426 (138038 to 179253) | 34120.79 (29744.45 to 38594.61) | 202444 (177104 to 228079) | 37731.67 (32923.22 to 42591.4) | 0.37 (0.34 to 0.39) |
| Lebanon | 69129 (60361 to 78927) | 27156.25 (23785.24 to 30912.95) | 236237 (207055 to 266318) | 31628.38 (27665.25 to 35697.88) | 0.49 (0.45 to 0.53) |
| Lesotho | 26478 (23176 to 30036) | 26911.63 (23597.2 to 30488.53) | 39299 (34199 to 44468) | 31806.34 (27792.29 to 35912.27) | 0.59 (0.56 to 0.62) |
| Liberia | 36055 (31578 to 40998) | 26312.23 (23106.52 to 29853.02) | 62203 (54617 to 70415) | 29703.65 (26154.28 to 33551.64) | 0.48 (0.44 to 0.53) |
| Libya | 60186 (52545 to 68178) | 28449.14 (24902.98 to 32185.73) | 168669 (147587 to 190256) | 31329.2 (27484.46 to 35287.7) | 0.31 (0.29 to 0.34) |
| Lithuania | 193809 (168807 to 219659) | 33459.46 (29167.41 to 37914.47) | 288737 (252758 to 325939) | 37323.65 (32604.2 to 42214.11) | 0.41 (0.38 to 0.44) |
| Luxembourg | 24352 (21593 to 27439) | 34017.66 (30142.04 to 38336.38) | 47312 (42073 to 52920) | 35182.16 (31247.79 to 39383.69) | 0.09 (0.08 to 0.11) |
| Madagascar | 132967 (116192 to 152309) | 23679.77 (20766.22 to 27044.83) | 278105 (242448 to 316690) | 25036.89 (21962.23 to 28385.58) | 0.19 (0.18 to 0.2) |
| Malawi | 104777 (91261 to 119651) | 24744.51 (21678.72 to 28144.36) | 217394 (189911 to 246596) | 27407.75 (24032.22 to 31025.75) | 0.36 (0.35 to 0.37) |
| Malaysia | 260678 (226067 to 296894) | 25303.14 (22003.19 to 28776.87) | 1006647 (878502 to 1140997) | 29222.61 (25567.61 to 33070.4) | 0.46 (0.44 to 0.49) |
| Maldives | 2241 (1928 to 2562) | 24141.29 (20913.69 to 27515.14) | 10071 (8720 to 11470) | 28855.94 (25060.45 to 32826.25) | 0.58 (0.56 to 0.6) |
| Mali | 107470 (93124 to 122734) | 24572.44 (21398.07 to 27976.86) | 259443 (227378 to 294346) | 27125.55 (23846.7 to 30687.54) | 0.33 (0.32 to 0.33) |
| Malta | 18182 (16021 to 20359) | 33315.26 (29374.82 to 37296.83) | 47111 (41886 to 52779) | 35225.48 (31276.99 to 39515.53) | 0.15 (0.12 to 0.19) |
| Marshall Islands | 529 (462 to 601) | 29586.56 (25912.39 to 33541.28) | 1200 (1045 to 1358) | 32569.77 (28539.58 to 36705.47) | 0.28 (0.25 to 0.3) |
| Mauritania | 30974 (27140 to 35244) | 27088.66 (23771.13 to 30771.34) | 76086 (66557 to 86122) | 30518.02 (26775.18 to 34477.9) | 0.35 (0.34 to 0.37) |
| Mauritius | 22747 (19748 to 25858) | 26526.49 (23098.98 to 30095.95) | 72259 (62771 to 82316) | 30080.83 (26181.24 to 34202.57) | 0.43 (0.41 to 0.44) |
| Mexico | 1646323 (1443966 to 1855437) | 33965.7 (29840.46 to 38240.58) | 5858046 (5140098 to 6598451) | 38185 (33548.45 to 42974.37) | 0.42 (0.4 to 0.44) |
| Micronesia (Federated States of) | 1690 (1476 to 1914) | 29395.83 (25747.03 to 33221.85) | 2766 (2413 to 3138) | 33209.06 (29173.33 to 37515.15) | 0.38 (0.33 to 0.42) |
| Monaco | 3308 (2931 to 3716) | 34730.81 (30673.51 to 39115.44) | 4644 (4128 to 5193) | 36010.38 (31910.54 to 40354.76) | 0.11 (0.1 to 0.12) |
| Mongolia | 34439 (29732 to 39562) | 29057.66 (25120.74 to 33318.33) | 87244 (74321 to 101415) | 36652.3 (31423.11 to 42352.9) | 0.81 (0.76 to 0.86) |
| Montenegro | 24365 (21170 to 27652) | 32124.39 (27965.89 to 36422.4) | 44728 (38995 to 50818) | 34013.02 (29684.56 to 38593.98) | 0.23 (0.21 to 0.24) |
| Morocco | 444884 (388491 to 506007) | 26702.71 (23359.69 to 30324.24) | 1203285 (1054040 to 1366630) | 29225.56 (25675.05 to 33124.91) | 0.25 (0.22 to 0.28) |
| Mozambique | 155061 (135265 to 176812) | 24176.83 (21186.17 to 27494.76) | 310079 (269811 to 352925) | 26813.62 (23443.25 to 30432.31) | 0.35 (0.33 to 0.36) |
| Myanmar | 560214 (489311 to 637838) | 21582.87 (18917.72 to 24510.96) | 1480333 (1286373 to 1683341) | 25968.6 (22646.55 to 29472.74) | 0.67 (0.64 to 0.71) |
| Namibia | 20150 (17565 to 22827) | 27120.62 (23754.59 to 30638.35) | 46588 (40662 to 52745) | 30833.39 (27007.49 to 34826.79) | 0.38 (0.35 to 0.4) |
| Nauru | 143 (124 to 162) | 29781.67 (26095 to 33705.45) | 210 (184 to 237) | 34096.88 (29959.95 to 38492.52) | 0.42 (0.4 to 0.45) |
| Nepal | 229778 (200893 to 263694) | 22980.77 (20163.55 to 26273.12) | 733347 (642669 to 835203) | 26397.33 (23173.33 to 29992.16) | 0.47 (0.44 to 0.49) |
| Netherlands | 917549 (827405 to 1017786) | 35128.2 (31651.88 to 38992.39) | 1672730 (1485050 to 1871519) | 35722.98 (31663.64 to 40014.34) | -0.03 (-0.12 to 0.05) |
| New Zealand | 186747 (164591 to 209751) | 36075.75 (31781.98 to 40527.2) | 433940 (384864 to 486545) | 38875.6 (34449.67 to 43618.14) | 0.24 (0.22 to 0.25) |
| Nicaragua | 50698 (44371 to 57490) | 29556.05 (25913.99 to 33479.07) | 190764 (167535 to 215817) | 33358.39 (29333.21 to 37713.01) | 0.39 (0.37 to 0.41) |
| Niger | 71125 (61745 to 80911) | 24326.61 (21240.63 to 27582.11) | 228588 (199643 to 261619) | 25619.29 (22458.62 to 29221.8) | 0.18 (0.17 to 0.18) |
| Nigeria | 1412061 (1232700 to 1601645) | 28180.15 (24668.03 to 31905.17) | 2967024 (2589303 to 3358081) | 31015.46 (27160.63 to 35032.75) | 0.34 (0.32 to 0.36) |
| Niue | 89 (78 to 101) | 31453.08 (27560.96 to 35526.69) | 98 (86 to 110) | 35193.86 (30992.69 to 39455.23) | 0.37 (0.34 to 0.4) |
| North Macedonia | 65616 (57332 to 74632) | 29333.21 (25709.79 to 33278.62) | 140355 (122472 to 158784) | 32310.82 (28241.24 to 36485.95) | 0.36 (0.34 to 0.38) |
| Northern Mariana Islands | 450 (393 to 510) | 32094.9 (28176.52 to 36256.47) | 2011 (1757 to 2269) | 34169.74 (30032.68 to 38461.42) | 0.17 (0.14 to 0.21) |
| Norway | 319199 (282780 to 357833) | 34603.7 (30583.15 to 38859.49) | 474373 (421376 to 530540) | 36165.48 (32057.05 to 40507.24) | 0.21 (0.11 to 0.32) |
| Oman | 18671 (16168 to 21145) | 27254.85 (23680.09 to 30810.64) | 60447 (52892 to 68344) | 32092.78 (28187.24 to 36195.99) | 0.54 (0.53 to 0.56) |
| Pakistan | 1552141 (1343024 to 1775914) | 24112.42 (20904.02 to 27544.72) | 3676149 (3178222 to 4197864) | 28416.3 (24664.13 to 32363.32) | 0.56 (0.54 to 0.58) |
| Palau | 365 (320 to 412) | 31461.21 (27678.08 to 35431.46) | 926 (810 to 1045) | 34776.03 (30585.88 to 39123.2) | 0.29 (0.25 to 0.33) |
| Palestine | 26988 (23522 to 30799) | 26644 (23282.22 to 30348.93) | 81655 (71134 to 92545) | 30205.96 (26418.93 to 34147.76) | 0.38 (0.37 to 0.4) |
| Panama | 53316 (46661 to 60225) | 30292.88 (26529.84 to 34202.89) | 188925 (165864 to 212556) | 34415.35 (30206.74 to 38727.08) | 0.39 (0.38 to 0.4) |
| Papua New Guinea | 46173 (40290 to 52774) | 24493.5 (21483.71 to 27892.11) | 134262 (117024 to 152708) | 26835.21 (23521.03 to 30416.28) | 0.27 (0.26 to 0.28) |
| Paraguay | 85380 (74677 to 96228) | 32680.07 (28617.79 to 36805.07) | 240494 (210283 to 271132) | 34230.74 (29977.11 to 38560.43) | 0.14 (0.09 to 0.19) |
| Peru | 443737 (388749 to 501906) | 32386.28 (28414.97 to 36601.41) | 1449878 (1271626 to 1631417) | 35673.63 (31294.15 to 40138.55) | 0.29 (0.27 to 0.32) |
| Philippines | 763979 (662490 to 873450) | 24089.84 (20939.91 to 27483.27) | 2591056 (2238498 to 2957835) | 27829.88 (24127.48 to 31703.6) | 0.42 (0.39 to 0.46) |
| Poland | 1808361 (1572911 to 2059043) | 32132.69 (27986.45 to 36547.75) | 3544627 (3091612 to 4013397) | 35980.99 (31368.43 to 40744.16) | 0.39 (0.37 to 0.41) |
| Portugal | 602574 (533450 to 677425) | 32367.65 (28660.55 to 36377.65) | 1125766 (1001092 to 1260137) | 34801.32 (30845.58 to 39061.47) | 0.34 (0.25 to 0.43) |
| Puerto Rico | 166766 (146450 to 187618) | 35831.01 (31467.01 to 40311.25) | 371263 (327993 to 415415) | 38488.09 (33880.43 to 43158.69) | 0.26 (0.24 to 0.27) |
| Qatar | 2421 (2093 to 2744) | 30180.44 (26321.4 to 34038.4) | 23897 (20820 to 27017) | 32976.42 (29031.06 to 37097.25) | 0.26 (0.23 to 0.28) |
| Republic of Korea | 1395876 (1227793 to 1571289) | 43190.67 (38152.64 to 48453.7) | 5493494 (4856921 to 6142498) | 44160.31 (39085.46 to 49331.85) | 0.17 (0.07 to 0.28) |
| Republic of Moldova | 164005 (143472 to 185261) | 30219.49 (26502.41 to 34058.51) | 273810 (239799 to 308947) | 34191.32 (29971.84 to 38554.43) | 0.5 (0.43 to 0.57) |
| Romania | 1037387 (903372 to 1177922) | 29343.26 (25610.87 to 33261.26) | 1625936 (1425168 to 1836623) | 32021.97 (28035.98 to 36193.9) | 0.33 (0.31 to 0.35) |
| Russian Federation | 8768664 (7581779 to 10000622) | 38136.86 (33067.18 to 43413.49) | 12854468 (11166759 to 14616647) | 39308.02 (34186.94 to 44642.44) | 0.22 (0.18 to 0.26) |
| Rwanda | 74158 (64443 to 84613) | 24058.67 (21027.85 to 27361.52) | 182611 (158675 to 208365) | 26291.74 (22957.36 to 29907.15) | 0.32 (0.3 to 0.33) |
| Saint Kitts and Nevis | 1796 (1575 to 2026) | 33832.23 (29658.55 to 38163.45) | 3135 (2738 to 3538) | 36682.99 (32218.54 to 41250.89) | 0.26 (0.24 to 0.27) |
| Saint Lucia | 3535 (3098 to 3992) | 31471.21 (27603.8 to 35511.19) | 10523 (9207 to 11852) | 35119.99 (30754.95 to 39540.25) | 0.32 (0.29 to 0.36) |
| Saint Vincent and the Grenadines | 2901 (2546 to 3283) | 31435.92 (27601.01 to 35564.89) | 6306 (5514 to 7104) | 34397.04 (30130.33 to 38720.81) | 0.31 (0.29 to 0.33) |
| Samoa | 2951 (2589 to 3329) | 29919.96 (26334.17 to 33681.25) | 5552 (4849 to 6274) | 33290.59 (29156.33 to 37561.33) | 0.36 (0.35 to 0.37) |
| San Marino | 1580 (1395 to 1772) | 33965.13 (29967.13 to 38146.35) | 3355 (2988 to 3764) | 35361.55 (31392.11 to 39778.26) | 0.12 (0.1 to 0.13) |
| Sao Tome and Principe | 2202 (1919 to 2509) | 27812.49 (24304.99 to 31636.45) | 3925 (3426 to 4446) | 32824.77 (28742.74 to 37106.13) | 0.57 (0.55 to 0.59) |
| Saudi Arabia | 165576 (144975 to 188159) | 27585.34 (24219.26 to 31276.96) | 544334 (475477 to 615367) | 32145.95 (28285.86 to 36207.78) | 0.25 (0.15 to 0.35) |
| Senegal | 96839 (84480 to 110352) | 26415.04 (23110.74 to 30024.7) | 254755 (222548 to 289000) | 29172.53 (25562.59 to 33036.36) | 0.29 (0.27 to 0.3) |
| Serbia | 416548 (361847 to 472801) | 29424.66 (25691.48 to 33281.77) | 735432 (642625 to 830558) | 32556.73 (28428.08 to 36777.27) | 0.37 (0.36 to 0.39) |
| Seychelles | 1904 (1661 to 2168) | 26336.79 (22980.5 to 29985.1) | 4212 (3654 to 4768) | 30278.81 (26355.78 to 34226.11) | 0.44 (0.39 to 0.48) |
| Sierra Leone | 59889 (52194 to 68145) | 24927.57 (21765.16 to 28326.34) | 114767 (100608 to 130310) | 28070.98 (24653.29 to 31825.68) | 0.38 (0.36 to 0.41) |
| Singapore | 104858 (92387 to 117566) | 41978.7 (37079.98 to 46968.95) | 476094 (419610 to 531584) | 43100.11 (38058.32 to 48061.59) | 0.08 (0.06 to 0.1) |
| Slovakia | 253585 (220208 to 288106) | 32542.38 (28282.5 to 36929.57) | 449671 (393630 to 509610) | 34969.92 (30612.42 to 39613.58) | 0.22 (0.2 to 0.24) |
| Slovenia | 102459 (89159 to 116661) | 32704.93 (28470.54 to 37216.12) | 203992 (178447 to 230653) | 34411 (30042.7 to 38967.44) | 0.16 (0.13 to 0.18) |
| Solomon Islands | 3730 (3248 to 4265) | 26186.25 (22937.04 to 29810.1) | 10514 (9204 to 11903) | 30121.98 (26461.92 to 34034.72) | 0.44 (0.42 to 0.46) |
| Somalia | 51631 (45013 to 59155) | 24530.9 (21501.43 to 27990.42) | 154819 (134169 to 176999) | 25661.17 (22423.96 to 29185.23) | 0.17 (0.16 to 0.18) |
| South Africa | 814362 (710626 to 920595) | 34133.86 (29841.13 to 38540.73) | 2001432 (1744856 to 2261451) | 36693.6 (32069.65 to 41400.94) | 0.26 (0.25 to 0.28) |
| South Sudan | 70884 (61766 to 80820) | 23940.65 (20931.09 to 27220.49) | 97097 (84533 to 111116) | 25533.09 (22352.24 to 29119.72) | 0.24 (0.23 to 0.26) |
| Spain | 2429852 (2143602 to 2741735) | 33198.35 (29284.72 to 37463.7) | 4419720 (3932968 to 4943790) | 35315.3 (31330.32 to 39587.98) | 0.16 (0.13 to 0.19) |
| Sri Lanka | 278836 (243771 to 317342) | 23069.58 (20221.42 to 26205.21) | 947047 (820880 to 1080086) | 27001.96 (23449.73 to 30751.88) | 0.53 (0.51 to 0.54) |
| Sudan | 240117 (209398 to 275326) | 22835.86 (19957.25 to 26120.57) | 566914 (496764 to 643156) | 27493.11 (24145.48 to 31145.12) | 0.63 (0.59 to 0.67) |
| Suriname | 9912 (8701 to 11197) | 33516.95 (29489.46 to 37817.1) | 27937 (24453 to 31408) | 36227.28 (31766.51 to 40691.42) | 0.28 (0.27 to 0.29) |
| Sweden | 598391 (518797 to 683661) | 29526.3 (25534.58 to 33820.83) | 891551 (778181 to 1014449) | 31580.4 (27449.63 to 36039.23) | 0.34 (0.14 to 0.54) |
| Switzerland | 453619 (401079 to 509044) | 33510.2 (29550.37 to 37665.36) | 799014 (709710 to 896616) | 34316.87 (30393.17 to 38590.88) | 0.07 (0.07 to 0.08) |
| Syrian Arab Republic | 149380 (130484 to 169959) | 25849.44 (22656.39 to 29357.33) | 458813 (399553 to 518731) | 29666.7 (25942.26 to 33466.7) | 0.47 (0.46 to 0.49) |
| Taiwan (Province of China) | 577537 (504451 to 657041) | 29798.8 (26119.9 to 33795.13) | 1963265 (1725263 to 2214684) | 34534.16 (30370.55 to 38939.6) | 0.53 (0.51 to 0.55) |
| Tajikistan | 86591 (74899 to 99604) | 27489.26 (23837.7 to 31581.92) | 184381 (157362 to 212726) | 29574.39 (25449.59 to 33941.32) | 0.25 (0.19 to 0.32) |
| Thailand | 889571 (775604 to 1010988) | 22977.57 (20099.72 to 26075.08) | 3934892 (3444570 to 4461828) | 27872.46 (24422 to 31596.76) | 0.66 (0.65 to 0.67) |
| Timor-Leste | 5379 (4673 to 6125) | 20838.47 (18197.92 to 23642.48) | 25808 (22393 to 29479) | 24559.23 (21359.22 to 27983.28) | 0.63 (0.59 to 0.67) |
| Togo | 33061 (28754 to 37746) | 25657.24 (22403.03 to 29222.42) | 113747 (99526 to 129422) | 29101.14 (25569.31 to 32998.12) | 0.4 (0.38 to 0.42) |
| Tokelau | 53 (46 to 60) | 28696.25 (25191.05 to 32420.46) | 65 (57 to 73) | 33706.1 (29672.1 to 37975.35) | 0.54 (0.51 to 0.57) |
| Tonga | 1883 (1649 to 2131) | 29176.11 (25620.85 to 32968.74) | 3128 (2762 to 3539) | 32602.06 (28802.91 to 36875.42) | 0.31 (0.26 to 0.36) |
| Trinidad and Tobago | 34835 (30506 to 39442) | 33793.2 (29614.54 to 38237.39) | 92540 (81181 to 104426) | 36221.65 (31829.41 to 40830.3) | 0.26 (0.24 to 0.28) |
| Tunisia | 156947 (136876 to 178648) | 26590.12 (23267.64 to 30212.33) | 509654 (445324 to 577871) | 30634.7 (26825.85 to 34680.06) | 0.46 (0.45 to 0.48) |
| Turkey | 1044855 (910308 to 1193787) | 26652.26 (23302.75 to 30379.72) | 3646606 (3197094 to 4114623) | 31274.3 (27459.1 to 35241.67) | 0.52 (0.45 to 0.59) |
| Turkmenistan | 64907 (55877 to 74469) | 30037.88 (25968.58 to 34376.45) | 159513 (136651 to 184058) | 35162.34 (30273.95 to 40399.69) | 0.56 (0.5 to 0.62) |
| Tuvalu | 230 (200 to 261) | 28756.65 (25127.16 to 32519.48) | 427 (373 to 483) | 33149.15 (29106.73 to 37452.16) | 0.44 (0.41 to 0.48) |
| Uganda | 172158 (150305 to 196908) | 24198.74 (21200.19 to 27611.08) | 411748 (360246 to 467667) | 26804.16 (23527.92 to 30358.82) | 0.34 (0.33 to 0.34) |
| Ukraine | 3236189 (2808887 to 3681581) | 34453.96 (29962.79 to 39132.32) | 3927722 (3415803 to 4454670) | 37123.97 (32295.07 to 42096.42) | 0.27 (0.25 to 0.29) |
| United Arab Emirates | 9457 (8240 to 10750) | 28690.21 (25114.29 to 32514.36) | 94694 (82724 to 107163) | 30471.5 (26856.97 to 34319.83) | 0.2 (0.16 to 0.23) |
| United Kingdom | 4359762 (3865492 to 4878810) | 35892.95 (31767.07 to 40209.59) | 6389711 (5685407 to 7119285) | 37853.48 (33592.04 to 42245.95) | 0.07 (-0.05 to 0.18) |
| United Republic of Tanzania | 321401 (280420 to 364656) | 26388.36 (23109.16 to 29865.35) | 768489 (673042 to 866801) | 28066.9 (24644.83 to 31607.81) | 0.1 (0.05 to 0.14) |
| United States of America | 16935670 (14995873 to 18973548) | 39939.15 (35319.55 to 44777.08) | 33160430 (29391907 to 37073918) | 41995.9 (37203.28 to 46968.84) | 0 (-0.14 to 0.13) |
| United States Virgin Islands | 3387 (2982 to 3813) | 35320.09 (31169.16 to 39709.71) | 9608 (8460 to 10773) | 37785.97 (33250.02 to 42398.66) | 0.22 (0.2 to 0.24) |
| Uruguay | 178914 (157649 to 202199) | 34518.67 (30416.02 to 39007.99) | 271720 (241002 to 304587) | 37496.07 (33177.95 to 42108.64) | 0.27 (0.25 to 0.28) |
| Uzbekistan | 412057 (353570 to 474046) | 30559.64 (26271.21 to 35130.04) | 1003777 (860931 to 1155422) | 34382.19 (29639.84 to 39384.3) | 0.36 (0.32 to 0.41) |
| Vanuatu | 1660 (1450 to 1897) | 25024.34 (21958.07 to 28487.29) | 5229 (4580 to 5938) | 28099.89 (24699.87 to 31815.08) | 0.36 (0.36 to 0.37) |
| Venezuela (Bolivarian Republic of) | 360053 (315295 to 407099) | 32638 (28626.57 to 36862.82) | 1323581 (1154809 to 1492621) | 35218.78 (30802.11 to 39661.86) | 0.13 (0.07 to 0.19) |
| Viet Nam | 1025689 (895723 to 1166714) | 21066.74 (18433.29 to 23939.85) | 2886942 (2507950 to 3281642) | 24600.04 (21445.59 to 27928.79) | 0.55 (0.53 to 0.57) |
| Yemen | 117533 (101659 to 134535) | 22409.8 (19478.86 to 25567.56) | 393066 (342181 to 446093) | 25997.77 (22706.45 to 29444.51) | 0.57 (0.53 to 0.6) |
| Zambia | 79073 (68923 to 89909) | 26548.83 (23234.98 to 30097.51) | 196276 (170797 to 222368) | 28304.75 (24748.6 to 31989.22) | 0.23 (0.18 to 0.28) |
| Zimbabwe | 120647 (105994 to 137067) | 26509.99 (23358.19 to 30056.59) | 204496 (178070 to 233311) | 27615.93 (24164.78 to 31365.89) | 0.04 (0.01 to 0.08) |
