# Supplementary material for: Global, regional, and national burden of osteoarthritis in elderly from 1990 to 2021: Insights from the global burden of disease study 2021
Source: Medicine (Baltimore). 2026 May 15;105(20):e48799. doi: 10.1097/MD.0000000000048799 (PMC13183078; doi:10.1097/MD.0000000000048799)
Supplement: Supplementary file 2 [file medi-105-e48799-s007.doc]

**Supplementary Table S2** The incidence of osteoarthritis cases and rates in the elderly population across 204 countries and territories in 1990 and 2021, and the trends from 1990 to 2021.

| Country | Incidence | | | | |
| --- | --- | --- | --- | --- | --- |
|  | No.,1990 (95% UI) | ASIR, 1990 per 100,000 people (95% UI) | No.,2021, (95% UI) | ASIR, 2021 per 100,000 people (95% UI) | EAPC,1990-2021, (95% CI) |
| Afghanistan | 10015 (7634 to 12744) | 1158.73 (881.61 to 1476.16) | 10270 (7850 to 13064) | 1273.79 (973.13 to 1619.17) | 0.35 (0.32 to 0.38) |
| Albania | 3126 (2388 to 3931) | 1275.84 (972.72 to 1607.02) | 8331 (6439 to 10470) | 1384.66 (1068.84 to 1742.74) | 0.3 (0.29 to 0.31) |
| Algeria | 19483 (14800 to 24731) | 1277.78 (969.71 to 1623.98) | 61544 (46878 to 78265) | 1414.62 (1075.95 to 1801.16) | 0.33 (0.33 to 0.34) |
| American Samoa | 35 (26 to 45) | 1360.27 (1009.94 to 1753.83) | 84 (63 to 109) | 1444.95 (1082.15 to 1867.4) | 0.18 (0.15 to 0.2) |
| Andorra | 119 (92 to 149) | 1569.19 (1207.71 to 1967.73) | 321 (248 to 402) | 1682.91 (1303.8 to 2106.66) | 0.18 (0.15 to 0.2) |
| Angola | 5745 (4329 to 7342) | 1341.78 (1009.83 to 1716.71) | 18903 (14427 to 24016) | 1449.51 (1103.9 to 1844.66) | 0.25 (0.24 to 0.26) |
| Antigua and Barbuda | 105 (80 to 133) | 1563.58 (1190.51 to 1976.24) | 224 (172 to 285) | 1635.08 (1250.98 to 2076.19) | 0.14 (0.13 to 0.15) |
| Argentina | 66816 (51273 to 84101) | 1572.85 (1205.4 to 1983.07) | 121980 (93613 to 153955) | 1717.08 (1318.9 to 2164.94) | 0.2 (0.17 to 0.24) |
| Armenia | 4320 (3305 to 5475) | 1259.3 (964.44 to 1600.69) | 8375 (6345 to 10722) | 1369.41 (1038.02 to 1751.28) | 0.32 (0.3 to 0.34) |
| Australia | 41435 (31943 to 52214) | 1601.16 (1234.23 to 2018.44) | 100229 (76722 to 127178) | 1764.16 (1353.46 to 2233.84) | 0.24 (0.22 to 0.27) |
| Austria | 24463 (19016 to 30820) | 1601.23 (1245.97 to 2014.37) | 36805 (28487 to 46156) | 1691.19 (1314.3 to 2110.97) | 0.1 (0.08 to 0.12) |
| Azerbaijan | 7615 (5807 to 9737) | 1299.41 (990.56 to 1660.02) | 18178 (13795 to 23352) | 1385.04 (1051.12 to 1775.53) | 0.24 (0.21 to 0.27) |
| Bahamas | 286 (218 to 365) | 1612.23 (1225.61 to 2053.53) | 853 (656 to 1078) | 1674.49 (1283.53 to 2122.97) | 0.13 (0.12 to 0.14) |
| Bahrain | 255 (192 to 327) | 1369.49 (1030.11 to 1758.63) | 1532 (1163 to 1958) | 1451.07 (1098.51 to 1857.69) | 0.18 (0.17 to 0.19) |
| Bangladesh | 64932 (49393 to 82272) | 1200.75 (912.41 to 1524.11) | 224964 (171031 to 287198) | 1309.95 (994.97 to 1674.69) | 0.3 (0.28 to 0.32) |
| Barbados | 598 (450 to 763) | 1610.43 (1216.46 to 2051.49) | 1174 (902 to 1490) | 1676.3 (1287.63 to 2128.79) | 0.13 (0.12 to 0.14) |
| Belarus | 24867 (18960 to 31555) | 1443.6 (1099.54 to 1834.73) | 33901 (25950 to 42754) | 1526.68 (1168.31 to 1926.81) | 0.2 (0.19 to 0.21) |
| Belgium | 31862 (24767 to 40167) | 1579.77 (1229.16 to 1990.58) | 47903 (36937 to 60146) | 1681.53 (1300.36 to 2103.82) | 0.12 (0.1 to 0.14) |
| Belize | 174 (132 to 220) | 1525.86 (1157.69 to 1923.46) | 585 (447 to 742) | 1652.64 (1260.39 to 2098.26) | 0.24 (0.22 to 0.27) |
| Benin | 3056 (2331 to 3887) | 1285.54 (979.79 to 1636.61) | 8295 (6287 to 10487) | 1426.13 (1079.42 to 1806.85) | 0.33 (0.32 to 0.35) |
| Bermuda | 131 (100 to 166) | 1658.39 (1259.19 to 2100.9) | 305 (232 to 386) | 1711.29 (1301.57 to 2162.92) | 0.09 (0.08 to 0.1) |
| Bhutan | 333 (253 to 422) | 1231.18 (931.22 to 1562.56) | 990 (750 to 1264) | 1354.55 (1025.17 to 1729.65) | 0.33 (0.32 to 0.33) |
| Bolivia (Plurinational State of) | 5747 (4402 to 7271) | 1499.86 (1146.96 to 1901.25) | 18737 (14462 to 23570) | 1634.28 (1259.19 to 2059.81) | 0.27 (0.27 to 0.28) |
| Bosnia and Herzegovina | 6614 (5101 to 8358) | 1335.75 (1029.05 to 1696.34) | 12257 (9403 to 15593) | 1445.67 (1108.35 to 1840.59) | 0.28 (0.25 to 0.31) |
| Botswana | 932 (716 to 1178) | 1368.97 (1047.62 to 1734.68) | 2641 (2023 to 3330) | 1528.04 (1167.43 to 1932.05) | 0.34 (0.32 to 0.35) |
| Brazil | 174562 (133562 to 220633) | 1617.58 (1234.74 to 2048.13) | 558917 (427868 to 707590) | 1750.72 (1339.36 to 2217.27) | 0.27 (0.26 to 0.28) |
| Brunei Darussalam | 202 (155 to 256) | 1823.58 (1401.98 to 2314.99) | 840 (646 to 1055) | 1949.06 (1494.34 to 2461.73) | 0.16 (0.13 to 0.18) |
| Bulgaria | 24340 (18612 to 30899) | 1422.79 (1083.81 to 1811.49) | 28268 (21487 to 35933) | 1488.32 (1129.23 to 1893.93) | 0.14 (0.13 to 0.15) |
| Burkina Faso | 6442 (4911 to 8208) | 1229.55 (935.88 to 1568.13) | 14058 (10677 to 17823) | 1316.6 (999.44 to 1671.84) | 0.22 (0.21 to 0.22) |
| Burundi | 3407 (2622 to 4286) | 1252.94 (962.77 to 1578.84) | 7094 (5421 to 8945) | 1275.29 (972.32 to 1614.15) | 0.06 (0.06 to 0.07) |
| Cabo Verde | 378 (288 to 483) | 1296.76 (987.1 to 1654.15) | 792 (597 to 1015) | 1450.56 (1093.11 to 1858.94) | 0.38 (0.37 to 0.39) |
| Cambodia | 5095 (3842 to 6499) | 1000.9 (754.74 to 1279.03) | 16612 (12585 to 21255) | 1121.34 (847.29 to 1435.63) | 0.41 (0.39 to 0.42) |
| Cameroon | 6952 (5294 to 8820) | 1332.5 (1014.34 to 1693.72) | 20481 (15507 to 25955) | 1438.31 (1087.22 to 1826.14) | 0.23 (0.21 to 0.24) |
| Canada | 50651 (39236 to 64129) | 1196.43 (926.11 to 1515.79) | 124800 (95754 to 158210) | 1318.12 (1012.17 to 1670.03) | 0.09 (-0.03 to 0.22) |
| Central African Republic | 1644 (1256 to 2079) | 1280.44 (975.21 to 1624.36) | 3107 (2381 to 3915) | 1331.04 (1016.43 to 1683.55) | 0.11 (0.11 to 0.12) |
| Chad | 4144 (3139 to 5242) | 1227.09 (928.43 to 1553.2) | 8250 (6313 to 10422) | 1274.17 (973.99 to 1612.44) | 0.11 (0.11 to 0.12) |
| Chile | 19750 (15103 to 24908) | 1578.26 (1205.9 to 1993.39) | 57512 (44303 to 72791) | 1731.93 (1334.04 to 2192.8) | 0.22 (0.19 to 0.25) |
| China | 1261988 (939532 to 1638864) | 1255.03 (933.9 to 1629.05) | 3818742 (2859541 to 4905030) | 1418.97 (1060.71 to 1824.43) | 0.33 (0.21 to 0.44) |
| Colombia | 31062 (23771 to 39370) | 1495.55 (1143.94 to 1898.1) | 112497 (85977 to 142917) | 1608.46 (1229.71 to 2042.03) | 0.26 (0.25 to 0.26) |
| Comoros | 297 (226 to 376) | 1276.9 (970.26 to 1621.83) | 786 (601 to 995) | 1378.46 (1051.6 to 1746.03) | 0.27 (0.26 to 0.28) |
| Congo | 1759 (1345 to 2228) | 1374.54 (1047.97 to 1745.62) | 4289 (3284 to 5477) | 1459.83 (1114.1 to 1865.71) | 0.19 (0.18 to 0.19) |
| Cook Islands | 20 (15 to 25) | 1315.72 (988.33 to 1696.48) | 50 (37 to 64) | 1446.43 (1088.31 to 1856.66) | 0.31 (0.28 to 0.33) |
| Costa Rica | 3190 (2433 to 4054) | 1516.16 (1156.04 to 1927.37) | 11477 (8746 to 14562) | 1627.67 (1240.14 to 2064.71) | 0.23 (0.22 to 0.24) |
| Coted'Ivoire | 5607 (4286 to 7097) | 1292.55 (986.5 to 1639.84) | 17058 (12977 to 21590) | 1387.3 (1053.5 to 1761.23) | 0.22 (0.21 to 0.23) |
| Croatia | 11419 (8768 to 14494) | 1417.71 (1085.57 to 1802.75) | 17403 (13205 to 22031) | 1479.55 (1122 to 1872.32) | 0.17 (0.16 to 0.18) |
| Cuba | 19538 (14959 to 24780) | 1523.85 (1166.61 to 1933.91) | 38892 (29600 to 49290) | 1625.62 (1238.99 to 2057.62) | 0.24 (0.23 to 0.25) |
| Cyprus | 1688 (1306 to 2111) | 1531.49 (1183.87 to 1916.11) | 4490 (3489 to 5608) | 1651.3 (1283.96 to 2062.29) | 0.17 (0.15 to 0.19) |
| Czechia | 26474 (20096 to 33817) | 1436.61 (1089.07 to 1836.82) | 41230 (31421 to 52399) | 1493.38 (1138.49 to 1897.61) | 0.13 (0.12 to 0.14) |
| Democratic People's Republic of Korea | 22059 (16347 to 28682) | 1210.63 (898.52 to 1571.11) | 52007 (39014 to 66904) | 1293.39 (970.79 to 1664.63) | 0.21 (0.2 to 0.22) |
| Democratic Republic of the Congo | 24283 (18550 to 30634) | 1317.2 (1002.65 to 1665.31) | 55159 (42540 to 69619) | 1355.2 (1040.11 to 1715.09) | 0.06 (0.03 to 0.09) |
| Denmark | 16248 (12533 to 20399) | 1595.33 (1233.54 to 1999.37) | 24425 (18757 to 30699) | 1660.98 (1280.44 to 2080.08) | 0.06 (0.04 to 0.08) |
| Djibouti | 179 (138 to 228) | 1268.11 (969.05 to 1612.94) | 981 (741 to 1260) | 1401.27 (1057.08 to 1802.66) | 0.37 (0.35 to 0.39) |
| Dominica | 119 (91 to 152) | 1548.43 (1182.73 to 1966.47) | 178 (136 to 226) | 1618.28 (1231.45 to 2052.21) | 0.12 (0.08 to 0.15) |
| Dominican Republic | 6700 (5131 to 8504) | 1511.93 (1156.47 to 1921.4) | 19862 (15139 to 25159) | 1631.73 (1243.44 to 2067.7) | 0.27 (0.26 to 0.27) |
| Ecuador | 9891 (7545 to 12541) | 1582.92 (1206.9 to 2009.16) | 34523 (26272 to 43932) | 1695.89 (1290.35 to 2158.98) | 0.27 (0.25 to 0.28) |
| Egypt | 40230 (30791 to 51356) | 1287.5 (982.94 to 1645.92) | 108826 (82186 to 138118) | 1407.04 (1060.91 to 1789.04) | 0.22 (0.2 to 0.25) |
| El Salvador | 5364 (4088 to 6826) | 1502.25 (1144.53 to 1912.33) | 12285 (9396 to 15720) | 1627.57 (1246.26 to 2079.91) | 0.27 (0.25 to 0.28) |
| Equatorial Guinea | 280 (215 to 356) | 1265.53 (968.34 to 1610.68) | 820 (626 to 1038) | 1504.41 (1144.35 to 1906.33) | 0.67 (0.63 to 0.71) |
| Eritrea | 1366 (1049 to 1721) | 1237.39 (947.24 to 1566.11) | 3898 (2984 to 4921) | 1326.23 (1011.8 to 1678) | 0.23 (0.22 to 0.23) |
| Estonia | 3935 (3006 to 4973) | 1465.74 (1119.36 to 1855.84) | 5340 (4052 to 6806) | 1545.84 (1174.96 to 1969.17) | 0.21 (0.19 to 0.22) |
| Eswatini | 449 (344 to 569) | 1403.91 (1071.93 to 1781.47) | 1008 (776 to 1261) | 1537.28 (1179.67 to 1927.88) | 0.26 (0.23 to 0.29) |
| Ethiopia | 30067 (23053 to 37909) | 1327.32 (1013.51 to 1680.19) | 73667 (56208 to 93497) | 1507.13 (1147.96 to 1915.57) | 0.46 (0.44 to 0.48) |
| Fiji | 480 (361 to 617) | 1267.6 (953.64 to 1630.88) | 1340 (1000 to 1721) | 1410.81 (1053.39 to 1815.46) | 0.35 (0.33 to 0.37) |
| Finland | 14886 (11458 to 18633) | 1602.55 (1234.59 to 2004.33) | 26599 (20571 to 33357) | 1694.73 (1316.77 to 2113.69) | 0.12 (0.1 to 0.14) |
| France | 167529 (129543 to 211323) | 1580.14 (1223.61 to 1992.11) | 283614 (219041 to 355287) | 1681.28 (1302.35 to 2097.12) | 0.14 (0.11 to 0.16) |
| Gabon | 967 (732 to 1227) | 1355.69 (1025.23 to 1723.9) | 1867 (1409 to 2374) | 1494.97 (1126.88 to 1904.09) | 0.29 (0.27 to 0.32) |
| Gambia | 510 (392 to 646) | 1284.73 (984.85 to 1631.28) | 1571 (1203 to 1988) | 1417.74 (1084.53 to 1795.95) | 0.31 (0.31 to 0.32) |
| Georgia | 10892 (8343 to 13840) | 1321.48 (1011.67 to 1679.38) | 11144 (8482 to 14263) | 1367.3 (1041.94 to 1747.3) | 0.12 (0.1 to 0.15) |
| Germany | 257689 (197772 to 322454) | 1617.16 (1242.54 to 2021.6) | 395069 (305288 to 496607) | 1699.03 (1319.46 to 2127.72) | 0.08 (0.06 to 0.1) |
| Ghana | 9798 (7410 to 12520) | 1362.9 (1030.3 to 1744.29) | 28560 (21754 to 36040) | 1465.32 (1112.89 to 1853.51) | 0.24 (0.2 to 0.28) |
| Greece | 31214 (24204 to 39260) | 1554.13 (1205.16 to 1955.5) | 46351 (35673 to 58568) | 1674.93 (1295.4 to 2106.05) | 0.31 (0.26 to 0.36) |
| Greenland | 47 (36 to 59) | 1240.98 (953.11 to 1559.37) | 128 (99 to 160) | 1349.18 (1037.71 to 1698.66) | 0.21 (0.18 to 0.23) |
| Grenada | 141 (107 to 177) | 1521.52 (1163.8 to 1916.12) | 230 (176 to 291) | 1611.71 (1232.92 to 2041.39) | 0.19 (0.16 to 0.21) |
| Guam | 121 (90 to 155) | 1322.83 (989.29 to 1697.22) | 399 (300 to 517) | 1439.93 (1082.48 to 1861.76) | 0.28 (0.27 to 0.3) |
| Guatemala | 5911 (4572 to 7441) | 1432.79 (1103.83 to 1809.85) | 20884 (15992 to 26451) | 1543.19 (1180.19 to 1957.08) | 0.24 (0.24 to 0.25) |
| Guinea | 5170 (3950 to 6559) | 1245.1 (949.65 to 1582.3) | 8685 (6663 to 11057) | 1319.68 (1010.81 to 1681.61) | 0.16 (0.15 to 0.17) |
| Guinea-Bissau | 580 (442 to 735) | 1258.91 (957.35 to 1597.52) | 1063 (815 to 1339) | 1344.73 (1028.65 to 1699.86) | 0.2 (0.19 to 0.21) |
| Guyana | 681 (523 to 865) | 1508.31 (1156.17 to 1919.65) | 1315 (1009 to 1662) | 1615.59 (1235.38 to 2045.15) | 0.23 (0.22 to 0.24) |
| Haiti | 5408 (4169 to 6848) | 1389.39 (1067.19 to 1764.34) | 12342 (9534 to 15450) | 1471.04 (1133.3 to 1847.77) | 0.21 (0.2 to 0.21) |
| Honduras | 3502 (2698 to 4435) | 1454.89 (1119.43 to 1845.8) | 12064 (9240 to 15212) | 1559 (1192.8 to 1969.01) | 0.23 (0.22 to 0.23) |
| Hungary | 28702 (21805 to 36437) | 1452.65 (1101.58 to 1846.77) | 39042 (29812 to 49467) | 1514.94 (1156.14 to 1917.23) | 0.12 (0.11 to 0.13) |
| Iceland | 591 (458 to 739) | 1625.93 (1260.61 to 2030.13) | 1271 (983 to 1590) | 1707.41 (1321.86 to 2131.99) | 0.06 (-0.01 to 0.13) |
| India | 701306 (534923 to 888911) | 1322.6 (1005.85 to 1681.09) | 2178794 (1657519 to 2766526) | 1471.43 (1117.07 to 1870.64) | 0.32 (0.29 to 0.36) |
| Indonesia | 123775 (93656 to 158144) | 1127.73 (851.95 to 1442.93) | 364470 (275915 to 466216) | 1271.74 (960.68 to 1629.27) | 0.4 (0.39 to 0.41) |
| Iran (Islamic Republic of) | 42979 (32927 to 54349) | 1342.55 (1023.56 to 1702.39) | 138500 (105829 to 176032) | 1476.72 (1126.31 to 1878.19) | 0.32 (0.32 to 0.33) |
| Iraq | 12126 (9212 to 15465) | 1332.16 (1011.36 to 1699.32) | 38134 (28913 to 48608) | 1404.31 (1062.96 to 1792.47) | 0.15 (0.14 to 0.16) |
| Ireland | 8465 (6549 to 10668) | 1577.46 (1220.11 to 1987.42) | 16906 (12956 to 21246) | 1693.44 (1299.91 to 2124.22) | 0.15 (0.13 to 0.18) |
| Israel | 10091 (7833 to 12683) | 1582.14 (1227.7 to 1987.58) | 26272 (20392 to 32792) | 1691.88 (1315.06 to 2105.89) | 0.04 (-0.02 to 0.1) |
| Italy | 195385 (151559 to 244326) | 1659.5 (1286.97 to 2076.42) | 297528 (229451 to 373626) | 1756.5 (1363.46 to 2193.45) | 0.18 (0.15 to 0.2) |
| Jamaica | 3487 (2668 to 4417) | 1532.44 (1173.48 to 1939.65) | 6312 (4844 to 7968) | 1618.65 (1243.13 to 2040.44) | 0.2 (0.18 to 0.21) |
| Japan | 432922 (334190 to 544316) | 1958.04 (1510.33 to 2465.33) | 801509 (613159 to 1017112) | 1994.84 (1537.95 to 2513.41) | 0.02 (0.01 to 0.04) |
| Jordan | 1920 (1455 to 2446) | 1329.18 (1005.6 to 1695.7) | 12169 (9229 to 15522) | 1447.79 (1097.15 to 1850.57) | 0.3 (0.28 to 0.32) |
| Kazakhstan | 21206 (15965 to 27280) | 1347.07 (1014.67 to 1733.14) | 32693 (24670 to 42063) | 1429.02 (1078.43 to 1839.16) | 0.2 (0.18 to 0.21) |
| Kenya | 13369 (10237 to 16910) | 1399.83 (1069.3 to 1774.31) | 41582 (31842 to 52616) | 1551.88 (1184.34 to 1969.36) | 0.35 (0.33 to 0.37) |
| Kiribati | 55 (41 to 70) | 1295.79 (975.2 to 1669.36) | 119 (89 to 153) | 1394.9 (1049.85 to 1795.34) | 0.21 (0.19 to 0.24) |
| Kuwait | 815 (622 to 1037) | 1351.54 (1028.94 to 1724.32) | 4332 (3265 to 5534) | 1469.95 (1106.04 to 1884.58) | 0.3 (0.29 to 0.31) |
| Kyrgyzstan | 4845 (3691 to 6225) | 1287.45 (981.28 to 1653.24) | 7895 (5998 to 10153) | 1345.63 (1023.56 to 1725.26) | 0.15 (0.12 to 0.17) |
| Lao People's Democratic Republic | 2413 (1822 to 3095) | 1006.21 (759.03 to 1291.4) | 5909 (4443 to 7528) | 1118.06 (840.91 to 1426.37) | 0.38 (0.36 to 0.4) |
| Latvia | 6900 (5272 to 8737) | 1469.77 (1122.33 to 1863.46) | 7921 (6023 to 10069) | 1541.89 (1173.93 to 1958.97) | 0.18 (0.17 to 0.18) |
| Lebanon | 3506 (2656 to 4465) | 1295.88 (980.53 to 1654.2) | 10397 (7838 to 13236) | 1441.53 (1087.78 to 1833.48) | 0.33 (0.29 to 0.37) |
| Lesotho | 1355 (1048 to 1727) | 1326.99 (1024.52 to 1692.98) | 1969 (1510 to 2487) | 1479.43 (1131.15 to 1873.65) | 0.36 (0.34 to 0.37) |
| Liberia | 1846 (1413 to 2348) | 1292.37 (986.85 to 1644.99) | 3136 (2379 to 3973) | 1407.87 (1066.45 to 1786.97) | 0.34 (0.31 to 0.36) |
| Libya | 2941 (2224 to 3745) | 1336.07 (1009.9 to 1701.37) | 8078 (6148 to 10264) | 1433.77 (1090.1 to 1823.06) | 0.24 (0.22 to 0.25) |
| Lithuania | 8525 (6520 to 10738) | 1455.5 (1113.09 to 1836.76) | 11382 (8704 to 14445) | 1531.41 (1172.31 to 1942.47) | 0.19 (0.17 to 0.2) |
| Luxembourg | 1138 (884 to 1429) | 1603.29 (1245.92 to 2014.4) | 2191 (1699 to 2755) | 1683.94 (1307.57 to 2115.23) | 0.08 (0.06 to 0.11) |
| Madagascar | 7332 (5648 to 9328) | 1222.63 (939.05 to 1557.59) | 15738 (12155 to 19856) | 1279.36 (984.02 to 1618.42) | 0.15 (0.15 to 0.16) |
| Malawi | 5786 (4401 to 7296) | 1263.35 (958.26 to 1596.51) | 11485 (8751 to 14513) | 1354.97 (1029.92 to 1715.12) | 0.27 (0.26 to 0.28) |
| Malaysia | 11850 (8917 to 15271) | 1116.82 (840.76 to 1438.43) | 44165 (33158 to 56744) | 1235.86 (927.25 to 1588.78) | 0.33 (0.32 to 0.34) |
| Maldives | 106 (80 to 136) | 1055.25 (794.42 to 1350.88) | 442 (329 to 566) | 1213.54 (905.49 to 1557.96) | 0.49 (0.47 to 0.5) |
| Mali | 5845 (4456 to 7446) | 1237.99 (942.12 to 1580.26) | 13566 (10340 to 17253) | 1326.62 (1010.07 to 1690.48) | 0.23 (0.23 to 0.24) |
| Malta | 875 (677 to 1089) | 1575.74 (1219.45 to 1963.43) | 2137 (1656 to 2684) | 1687.14 (1311.17 to 2112.25) | 0.12 (0.09 to 0.16) |
| Marshall Islands | 24 (18 to 30) | 1261.13 (952.57 to 1620.37) | 55 (41 to 70) | 1357.76 (1026.58 to 1735.52) | 0.22 (0.2 to 0.23) |
| Mauritania | 1556 (1183 to 1981) | 1323.34 (1004.62 to 1685.88) | 3753 (2856 to 4768) | 1436.94 (1092.45 to 1828.55) | 0.24 (0.23 to 0.25) |
| Mauritius | 1034 (775 to 1335) | 1149.06 (860.7 to 1483.97) | 3117 (2349 to 4037) | 1259.64 (948.22 to 1630.47) | 0.32 (0.31 to 0.33) |
| Mexico | 82627 (63293 to 104421) | 1643.78 (1257.87 to 2080.25) | 276854 (212627 to 349051) | 1758.73 (1349.42 to 2219.37) | 0.23 (0.22 to 0.24) |
| Micronesia (Federated States of) | 76 (58 to 98) | 1275.21 (959.62 to 1641.3) | 128 (97 to 165) | 1396.06 (1053.8 to 1800.75) | 0.29 (0.26 to 0.33) |
| Monaco | 144 (111 to 182) | 1632.51 (1264.27 to 2054.04) | 205 (157 to 259) | 1727.04 (1327.35 to 2174.12) | 0.12 (0.1 to 0.14) |
| Mongolia | 1524 (1167 to 1940) | 1252.67 (956.97 to 1596.3) | 3523 (2671 to 4544) | 1362.21 (1033.96 to 1754.36) | 0.25 (0.22 to 0.27) |
| Montenegro | 1121 (854 to 1428) | 1434.9 (1094.07 to 1829.8) | 1991 (1513 to 2533) | 1481.99 (1123.75 to 1889.48) | 0.13 (0.12 to 0.14) |
| Morocco | 22035 (16733 to 28072) | 1277.15 (969.44 to 1628.99) | 59565 (45361 to 75646) | 1379.22 (1049.33 to 1753.36) | 0.23 (0.22 to 0.24) |
| Mozambique | 8553 (6585 to 10858) | 1240.27 (952.44 to 1577.26) | 16722 (12879 to 21049) | 1336.84 (1026.42 to 1688.72) | 0.26 (0.25 to 0.27) |
| Myanmar | 27905 (21088 to 35638) | 1020.15 (769.71 to 1305.33) | 69126 (51897 to 88934) | 1163.43 (872.89 to 1496.91) | 0.49 (0.47 to 0.51) |
| Namibia | 1077 (830 to 1354) | 1331.45 (1022.57 to 1679.68) | 2338 (1795 to 2945) | 1452.2 (1112.36 to 1833.25) | 0.25 (0.24 to 0.27) |
| Nauru | 7 (5 to 8) | 1265.61 (946.02 to 1636.03) | 9 (7 to 12) | 1419.69 (1066.28 to 1839.11) | 0.37 (0.36 to 0.38) |
| Nepal | 12653 (9689 to 16188) | 1176.59 (897.82 to 1507.49) | 38080 (28980 to 48530) | 1308.25 (994.13 to 1669.89) | 0.36 (0.34 to 0.38) |
| Netherlands | 41062 (31689 to 51612) | 1611.42 (1245.96 to 2023.1) | 76846 (59212 to 96838) | 1717.75 (1326.75 to 2159.71) | 0.11 (0.07 to 0.15) |
| New Zealand | 8719 (6665 to 11047) | 1688.82 (1290.5 to 2141.04) | 19983 (15338 to 25171) | 1837.08 (1412.56 to 2310.54) | 0.21 (0.18 to 0.23) |
| Nicaragua | 2596 (1987 to 3276) | 1458.06 (1114.49 to 1842.71) | 9298 (7163 to 11768) | 1580.45 (1215.98 to 2002.69) | 0.27 (0.26 to 0.28) |
| Niger | 3915 (2986 to 4983) | 1228.27 (934.5 to 1565.66) | 12373 (9459 to 15725) | 1286.91 (982.38 to 1636.25) | 0.15 (0.15 to 0.16) |
| Nigeria | 73200 (55878 to 92586) | 1392.37 (1060.26 to 1765) | 152713 (116603 to 193650) | 1500.41 (1143.5 to 1906.23) | 0.25 (0.24 to 0.26) |
| Niue | 4 (3 to 5) | 1326.08 (995.86 to 1703.01) | 4 (3 to 5) | 1446.41 (1081.87 to 1864.37) | 0.29 (0.27 to 0.31) |
| North Macedonia | 3223 (2459 to 4044) | 1373.48 (1046.54 to 1726.49) | 6519 (5016 to 8202) | 1448.67 (1111.28 to 1827.38) | 0.19 (0.18 to 0.19) |
| Northern Mariana Islands | 20 (15 to 26) | 1318.94 (989.89 to 1705.11) | 90 (67 to 115) | 1407.01 (1054.62 to 1808.22) | 0.2 (0.18 to 0.23) |
| Norway | 14367 (11094 to 17996) | 1648.83 (1278.08 to 2059.05) | 21848 (16935 to 27472) | 1756.37 (1365.46 to 2200.55) | 0.16 (0.13 to 0.19) |
| Oman | 921 (694 to 1173) | 1274.13 (959.65 to 1627.08) | 2963 (2239 to 3778) | 1445.84 (1093.36 to 1846.2) | 0.44 (0.42 to 0.45) |
| Pakistan | 80800 (61725 to 102779) | 1214.24 (925.55 to 1547.07) | 189184 (143923 to 241798) | 1375.14 (1043.41 to 1761.56) | 0.44 (0.42 to 0.46) |
| Palau | 16 (12 to 21) | 1318.91 (994.79 to 1695.89) | 41 (31 to 53) | 1424.84 (1065.03 to 1835.9) | 0.25 (0.22 to 0.27) |
| Palestine | 1369 (1048 to 1737) | 1295.75 (990.32 to 1646.55) | 4029 (3064 to 5098) | 1393.14 (1057.21 to 1767.93) | 0.21 (0.19 to 0.22) |
| Panama | 2605 (1990 to 3300) | 1456.83 (1112.65 to 1846.32) | 8702 (6687 to 11077) | 1595.61 (1226.84 to 2029.7) | 0.28 (0.27 to 0.29) |
| Papua New Guinea | 2311 (1750 to 2963) | 1134.72 (859.24 to 1456.62) | 6535 (4942 to 8342) | 1218.99 (921.02 to 1559.07) | 0.21 (0.2 to 0.22) |
| Paraguay | 4109 (3116 to 5220) | 1536.26 (1164.33 to 1952.37) | 11589 (8861 to 14732) | 1599.87 (1221.88 to 2035.27) | 0.14 (0.12 to 0.15) |
| Peru | 21670 (16622 to 27543) | 1541.69 (1181.54 to 1960.29) | 67632 (51841 to 85544) | 1660.02 (1272.39 to 2099.5) | 0.24 (0.23 to 0.25) |
| Philippines | 36932 (27988 to 47322) | 1115.72 (844.14 to 1431.38) | 123375 (93327 to 158494) | 1261.5 (952.94 to 1621.06) | 0.38 (0.36 to 0.41) |
| Poland | 87479 (66872 to 110770) | 1518.33 (1159.02 to 1926.11) | 159662 (122234 to 202924) | 1631.63 (1248.08 to 2074.62) | 0.24 (0.23 to 0.24) |
| Portugal | 29503 (22892 to 36831) | 1559.17 (1209.13 to 1947.87) | 49828 (38389 to 62918) | 1685.48 (1303.79 to 2117.12) | 0.15 (0.12 to 0.19) |
| Puerto Rico | 7644 (5833 to 9764) | 1645.69 (1255.6 to 2102.38) | 15637 (11849 to 19859) | 1734.9 (1318.47 to 2195.25) | 0.19 (0.18 to 0.21) |
| Qatar | 123 (92 to 155) | 1362.59 (1025.15 to 1733.73) | 1246 (952 to 1579) | 1470.14 (1116.83 to 1877.8) | 0.21 (0.19 to 0.22) |
| Republic of Korea | 68867 (52897 to 86757) | 1942.29 (1487.15 to 2456.25) | 252957 (194259 to 318192) | 1986.5 (1524.32 to 2502.33) | 0.11 (0.02 to 0.2) |
| Republic of Moldova | 7949 (6088 to 10069) | 1391.18 (1062.13 to 1766.06) | 12259 (9363 to 15561) | 1503.72 (1148.05 to 1908.86) | 0.3 (0.27 to 0.34) |
| Romania | 50491 (38712 to 63721) | 1374.71 (1051.45 to 1739.49) | 72435 (55221 to 92201) | 1463.36 (1115.91 to 1861.66) | 0.23 (0.22 to 0.23) |
| Russian Federation | 375393 (286294 to 477004) | 1568.47 (1196.04 to 1993.83) | 559307 (427476 to 711172) | 1674.89 (1278.97 to 2130.96) | 0.22 (0.21 to 0.23) |
| Rwanda | 4164 (3210 to 5281) | 1242.06 (954.7 to 1577.74) | 10023 (7695 to 12619) | 1329.72 (1017.46 to 1680.95) | 0.24 (0.23 to 0.25) |
| Saint Kitts and Nevis | 83 (64 to 107) | 1578.2 (1206.22 to 2015.95) | 159 (122 to 201) | 1671.07 (1278.52 to 2120.16) | 0.18 (0.17 to 0.19) |
| Saint Lucia | 175 (133 to 221) | 1526.2 (1164.69 to 1931.7) | 496 (381 to 626) | 1630.52 (1251.59 to 2059.55) | 0.2 (0.18 to 0.22) |
| Saint Vincent and the Grenadines | 142 (109 to 180) | 1519.81 (1164.68 to 1928.18) | 304 (232 to 385) | 1606.75 (1228.32 to 2037.54) | 0.2 (0.19 to 0.22) |
| Samoa | 133 (100 to 172) | 1289.23 (967.82 to 1667.04) | 242 (181 to 311) | 1395.11 (1045.1 to 1793.71) | 0.27 (0.26 to 0.28) |
| San Marino | 72 (56 to 92) | 1605.15 (1235.84 to 2026) | 147 (113 to 184) | 1703.64 (1317.48 to 2131.6) | 0.12 (0.09 to 0.14) |
| Sao Tome and Principe | 110 (84 to 140) | 1332.15 (1017.99 to 1694.14) | 187 (142 to 239) | 1470.81 (1116.15 to 1881.62) | 0.33 (0.32 to 0.33) |
| Saudi Arabia | 8212 (6229 to 10431) | 1300 (985.04 to 1654.75) | 27428 (20777 to 35066) | 1449.49 (1094.66 to 1860.81) | 0.34 (0.32 to 0.37) |
| Senegal | 4995 (3812 to 6341) | 1296.49 (988 to 1648.5) | 12766 (9651 to 16239) | 1388.34 (1049.35 to 1767.51) | 0.2 (0.19 to 0.22) |
| Serbia | 21146 (16308 to 26709) | 1383.48 (1061.33 to 1753.88) | 32862 (25150 to 41649) | 1469.22 (1125.03 to 1861.69) | 0.22 (0.21 to 0.22) |
| Seychelles | 84 (62 to 108) | 1154.39 (861.67 to 1487.01) | 183 (137 to 235) | 1260.16 (943.6 to 1620.83) | 0.28 (0.26 to 0.31) |
| Sierra Leone | 3088 (2356 to 3928) | 1250.59 (953.5 to 1592.07) | 5797 (4407 to 7339) | 1355.05 (1029.53 to 1717.69) | 0.25 (0.24 to 0.27) |
| Singapore | 4967 (3809 to 6248) | 1866.34 (1428.99 to 2352.9) | 22212 (17067 to 27962) | 1942.81 (1491.03 to 2447.78) | 0.06 (0.04 to 0.09) |
| Slovakia | 11352 (8651 to 14404) | 1432.13 (1090.55 to 1819.04) | 19315 (14770 to 24435) | 1498.94 (1144.03 to 1897.11) | 0.13 (0.12 to 0.14) |
| Slovenia | 4551 (3457 to 5768) | 1438.24 (1092.06 to 1825.31) | 8509 (6494 to 10840) | 1488.18 (1136.82 to 1892.82) | 0.11 (0.1 to 0.12) |
| Solomon Islands | 180 (135 to 231) | 1173.47 (881.74 to 1505.26) | 484 (364 to 620) | 1309.49 (985.42 to 1678.77) | 0.36 (0.34 to 0.38) |
| Somalia | 2896 (2217 to 3663) | 1250.47 (953.25 to 1585.88) | 8774 (6711 to 11053) | 1300.66 (991.29 to 1642.74) | 0.15 (0.14 to 0.15) |
| South Africa | 39320 (30107 to 49948) | 1585.92 (1212.36 to 2017.23) | 96664 (73887 to 122110) | 1681.86 (1282.83 to 2130.55) | 0.2 (0.2 to 0.21) |
| South Sudan | 3804 (2908 to 4837) | 1225.41 (935.55 to 1560.04) | 5336 (4090 to 6765) | 1299.51 (993.38 to 1651.29) | 0.22 (0.21 to 0.23) |
| Spain | 115918 (89949 to 144898) | 1584.09 (1228.91 to 1980.31) | 193915 (149863 to 243284) | 1694.96 (1314.48 to 2116.41) | 0.11 (0.07 to 0.15) |
| Sri Lanka | 13264 (10030 to 16962) | 1055.54 (797.22 to 1351.21) | 42776 (32237 to 55327) | 1187.74 (894.4 to 1536.77) | 0.4 (0.4 to 0.41) |
| Sudan | 12827 (9712 to 16433) | 1173.27 (888.02 to 1503.95) | 28709 (21799 to 36426) | 1332.18 (1010.42 to 1692.56) | 0.42 (0.4 to 0.44) |
| Suriname | 480 (366 to 608) | 1554.79 (1185.24 to 1972.61) | 1317 (1006 to 1678) | 1653.27 (1263.11 to 2106.37) | 0.22 (0.22 to 0.23) |
| Sweden | 28235 (21710 to 35380) | 1483.98 (1143.63 to 1855.74) | 40937 (31384 to 51894) | 1584.38 (1220.13 to 1999.78) | 0.2 (0.07 to 0.33) |
| Switzerland | 20274 (15607 to 25516) | 1580.93 (1219.28 to 1985.76) | 35756 (27432 to 44759) | 1656.94 (1274.32 to 2065.84) | 0.08 (0.06 to 0.1) |
| Syrian Arab Republic | 7778 (5949 to 9942) | 1272.41 (971.83 to 1625.68) | 22931 (17484 to 29226) | 1391.01 (1058.39 to 1776.91) | 0.29 (0.28 to 0.3) |
| Taiwan (Province of China) | 25076 (18487 to 32604) | 1235.1 (911.64 to 1604.7) | 79367 (58634 to 103469) | 1393.89 (1029.92 to 1816.38) | 0.44 (0.42 to 0.45) |
| Tajikistan | 3994 (3050 to 5090) | 1217.07 (929.43 to 1551.45) | 8628 (6653 to 11028) | 1259.36 (969.63 to 1608.75) | 0.11 (0.08 to 0.15) |
| Thailand | 42995 (32253 to 55156) | 1064.95 (798.75 to 1367.95) | 175290 (130727 to 227577) | 1225.76 (913.77 to 1590.5) | 0.48 (0.47 to 0.49) |
| Timor-Leste | 273 (207 to 345) | 992.51 (753.77 to 1258.8) | 1207 (912 to 1543) | 1112.12 (839.56 to 1421.68) | 0.43 (0.41 to 0.46) |
| Togo | 1743 (1325 to 2211) | 1272.44 (966.77 to 1617.41) | 5873 (4497 to 7490) | 1386.82 (1059.94 to 1771.79) | 0.28 (0.27 to 0.29) |
| Tokelau | 2 (2 to 3) | 1247.9 (938.46 to 1596.71) | 3 (2 to 3) | 1406.3 (1056.61 to 1812.2) | 0.4 (0.37 to 0.43) |
| Tonga | 85 (64 to 109) | 1268 (951.2 to 1625.42) | 134 (101 to 173) | 1384.73 (1041.05 to 1785.18) | 0.26 (0.23 to 0.3) |
| Trinidad and Tobago | 1656 (1262 to 2100) | 1576.53 (1200.09 to 2000.93) | 4372 (3328 to 5529) | 1661.27 (1263.86 to 2101.92) | 0.2 (0.18 to 0.21) |
| Tunisia | 8011 (6097 to 10293) | 1282.04 (973.88 to 1648.39) | 24351 (18524 to 30870) | 1408.99 (1071.02 to 1787.69) | 0.3 (0.29 to 0.31) |
| Turkey | 54593 (41393 to 69232) | 1314.19 (994.66 to 1669.57) | 173766 (131491 to 220422) | 1450.59 (1096.38 to 1840.88) | 0.33 (0.3 to 0.37) |
| Turkmenistan | 2960 (2270 to 3754) | 1289.56 (987.38 to 1636.68) | 6780 (5148 to 8704) | 1385.26 (1050.97 to 1773.59) | 0.26 (0.24 to 0.29) |
| Tuvalu | 11 (8 to 14) | 1264.14 (950.31 to 1623.32) | 19 (14 to 24) | 1392.15 (1047.14 to 1790.04) | 0.32 (0.3 to 0.34) |
| Uganda | 9312 (7127 to 11763) | 1238.8 (946.94 to 1568.24) | 21871 (16762 to 27539) | 1338.48 (1024.38 to 1689.56) | 0.27 (0.26 to 0.27) |
| Ukraine | 152634 (116580 to 194599) | 1574.65 (1201.04 to 2009.7) | 173659 (132670 to 221133) | 1633.15 (1247.44 to 2080.93) | 0.15 (0.13 to 0.17) |
| United Arab Emirates | 470 (354 to 599) | 1328.37 (1001.27 to 1694.08) | 5175 (3919 to 6601) | 1398.46 (1057.29 to 1787.23) | 0.17 (0.15 to 0.19) |
| United Kingdom | 203648 (158422 to 254651) | 1744.21 (1359.69 to 2177.19) | 290923 (225843 to 364527) | 1844.45 (1437.34 to 2302.44) | 0.08 (0.02 to 0.14) |
| United Republic of Tanzania | 16904 (12915 to 21361) | 1300.75 (991.89 to 1647.96) | 39585 (30486 to 49714) | 1373.85 (1056.33 to 1729.78) | 0.14 (0.12 to 0.16) |
| United States of America | 710854 (539085 to 903383) | 1727.49 (1310.74 to 2193.38) | 1439798 (1099592 to 1820360) | 1850.65 (1413.95 to 2336.75) | -0.06 (-0.18 to 0.07) |
| United States Virgin Islands | 165 (126 to 209) | 1629.67 (1245.7 to 2073.66) | 430 (326 to 543) | 1715.18 (1302.55 to 2168.49) | 0.16 (0.15 to 0.18) |
| Uruguay | 8154 (6297 to 10250) | 1571.51 (1213.51 to 1975.82) | 11771 (9025 to 14915) | 1721.01 (1323.56 to 2175.85) | 0.22 (0.19 to 0.25) |
| Uzbekistan | 17845 (13500 to 22744) | 1281.36 (970.31 to 1632.59) | 43322 (33037 to 55154) | 1362.57 (1038.92 to 1735.26) | 0.2 (0.18 to 0.23) |
| Vanuatu | 81 (61 to 103) | 1147.18 (867.57 to 1459.19) | 250 (189 to 318) | 1265.42 (954.21 to 1611.98) | 0.32 (0.31 to 0.32) |
| Venezuela (Bolivarian Republic of) | 17528 (13436 to 22182) | 1531.25 (1172.42 to 1939.72) | 63139 (48059 to 79920) | 1619.79 (1231.36 to 2050.72) | 0.12 (0.09 to 0.16) |
| Viet Nam | 50454 (38142 to 64708) | 1009.17 (762.41 to 1294.79) | 138159 (104095 to 177258) | 1128.43 (850.69 to 1448.89) | 0.4 (0.39 to 0.41) |
| Yemen | 6561 (5033 to 8291) | 1159.41 (887.47 to 1468.5) | 20489 (15759 to 25795) | 1278.24 (981.02 to 1613.36) | 0.36 (0.34 to 0.38) |
| Zambia | 4149 (3147 to 5283) | 1303.12 (987.89 to 1661.23) | 10250 (7829 to 12933) | 1379.54 (1051.29 to 1744.85) | 0.21 (0.18 to 0.23) |
| Zimbabwe | 6389 (4885 to 8098) | 1315.7 (1003.43 to 1670.87) | 11056 (8498 to 13861) | 1372.86 (1052.43 to 1725.01) | 0.08 (0.05 to 0.1) |
