# Supplementary material for: Global, regional, and national burden of osteoarthritis in elderly from 1990 to 2021: Insights from the global burden of disease study 2021
Source: Medicine (Baltimore). 2026 May 15;105(20):e48799. doi: 10.1097/MD.0000000000048799 (PMC13183078; doi:10.1097/MD.0000000000048799)
Supplement: Supplementary file 3 [file medi-105-e48799-s008.doc]

**Supplementary Table S3** The DALYs and rates of osteoarthritis in the elderly population across 204 countries and territories in 1990 and 2021, and the trends from 1990 to 2021.

| Country | DALYs | | | | |
| --- | --- | --- | --- | --- | --- |
|  | No.,1990 (95% UI) | ASDALYR, 1990 per 100,000 people (95% UI) | No.,2021, (95% UI) | ASDALYR, 2021 per 100,000 people (95% UI) | EAPC,1990-2021, (95% CI) |
| Afghanistan | 6038 (2935 to 12038) | 743 (361.63 to 1483.39) | 6706 (3271 to 13552) | 826.09 (403.61 to 1666.15) | 0.42 (0.36 to 0.47) |
| Albania | 2054 (1016 to 4115) | 873.62 (431.57 to 1750.44) | 5986 (2917 to 12116) | 1008.78 (491.96 to 2042.17) | 0.56 (0.53 to 0.59) |
| Algeria | 12990 (6255 to 26148) | 893.55 (432.17 to 1798.01) | 44649 (21893 to 90655) | 1067.35 (523.4 to 2169.62) | 0.58 (0.56 to 0.6) |
| American Samoa | 28 (14 to 57) | 1165.09 (563.53 to 2378.18) | 69 (33 to 141) | 1243 (601.98 to 2525.1) | 0.17 (0.12 to 0.21) |
| Andorra | 87 (42 to 175) | 1184.84 (577.4 to 2384.13) | 245 (120 to 493) | 1265.71 (619.51 to 2547.04) | 0.22 (0.2 to 0.25) |
| Angola | 4050 (1960 to 8209) | 1046.35 (506.89 to 2124.84) | 13670 (6615 to 27644) | 1155.35 (561.06 to 2339.59) | 0.34 (0.33 to 0.35) |
| Antigua and Barbuda | 85 (41 to 173) | 1208.07 (584.03 to 2461.96) | 168 (81 to 340) | 1278.62 (618.65 to 2583.91) | 0.17 (0.16 to 0.18) |
| Argentina | 51637 (24890 to 104050) | 1249.22 (602.59 to 2518.04) | 98730 (48154 to 200099) | 1362.28 (664.73 to 2759.5) | 0.27 (0.24 to 0.3) |
| Armenia | 3214 (1558 to 6467) | 1007.58 (489.5 to 2031.1) | 7263 (3531 to 14516) | 1226.11 (596.02 to 2454.57) | 0.76 (0.71 to 0.82) |
| Australia | 32760 (15956 to 65648) | 1270.32 (619.25 to 2543.71) | 83324 (41008 to 167843) | 1401.96 (689.97 to 2820.93) | 0.3 (0.28 to 0.32) |
| Austria | 19061 (9347 to 38429) | 1205.53 (591.64 to 2426.45) | 29624 (14521 to 59850) | 1267.56 (620.34 to 2557.84) | 0.14 (0.13 to 0.15) |
| Azerbaijan | 6431 (3093 to 12860) | 1158.87 (558.75 to 2321.1) | 14984 (7212 to 30119) | 1262.6 (608.5 to 2545.35) | 0.38 (0.28 to 0.48) |
| Bahamas | 221 (107 to 449) | 1263.98 (610.92 to 2571.44) | 639 (314 to 1305) | 1325.74 (650.74 to 2708.26) | 0.15 (0.13 to 0.17) |
| Bahrain | 177 (86 to 358) | 1051.57 (510.31 to 2130.59) | 1036 (505 to 2070) | 1121.84 (547.44 to 2249.84) | 0.21 (0.2 to 0.22) |
| Bangladesh | 42518 (20469 to 85597) | 813.22 (392.11 to 1637.55) | 156443 (75211 to 314529) | 937.94 (451.52 to 1885.75) | 0.49 (0.45 to 0.53) |
| Barbados | 489 (234 to 992) | 1246.76 (599.48 to 2526.93) | 920 (445 to 1888) | 1325.6 (641.37 to 2717.96) | 0.2 (0.18 to 0.22) |
| Belarus | 19913 (9758 to 40398) | 1191.72 (583.73 to 2417.88) | 29176 (14149 to 58699) | 1339.94 (649.94 to 2697.41) | 0.44 (0.42 to 0.47) |
| Belgium | 24682 (12084 to 49425) | 1202.48 (589.03 to 2405.89) | 38388 (18697 to 78040) | 1260.05 (613.09 to 2561.45) | 0.13 (0.11 to 0.14) |
| Belize | 127 (62 to 254) | 1119.03 (542.57 to 2236.44) | 429 (209 to 874) | 1269.24 (616.94 to 2587.56) | 0.37 (0.32 to 0.42) |
| Benin | 2044 (990 to 4105) | 882.56 (427.96 to 1772.68) | 5861 (2858 to 11764) | 1059.24 (516.2 to 2126.65) | 0.59 (0.57 to 0.61) |
| Bermuda | 103 (50 to 208) | 1326.17 (650.7 to 2682.04) | 251 (123 to 510) | 1375 (672.3 to 2794.7) | 0.11 (0.1 to 0.12) |
| Bhutan | 210 (102 to 425) | 841.45 (408.48 to 1701.07) | 695 (339 to 1396) | 968.1 (471.66 to 1941.98) | 0.47 (0.45 to 0.48) |
| Bolivia (Plurinational State of) | 3856 (1875 to 7812) | 1062.73 (517.44 to 2152.27) | 13255 (6425 to 26734) | 1212.47 (588.31 to 2447.26) | 0.43 (0.41 to 0.45) |
| Bosnia and Herzegovina | 4456 (2172 to 8999) | 959.82 (467.6 to 1940.45) | 9551 (4630 to 19296) | 1130.25 (547.68 to 2283.45) | 0.61 (0.53 to 0.68) |
| Botswana | 628 (303 to 1272) | 998.67 (483.36 to 2026.17) | 1903 (931 to 3871) | 1189.69 (581.25 to 2424.09) | 0.54 (0.52 to 0.56) |
| Brazil | 117388 (57129 to 237790) | 1143.58 (556.89 to 2316.71) | 407581 (199071 to 825036) | 1300.81 (635.38 to 2633.89) | 0.44 (0.42 to 0.45) |
| Brunei Darussalam | 155 (75 to 315) | 1474.37 (713.15 to 2992.02) | 609 (294 to 1230) | 1588.5 (768.53 to 3211.97) | 0.25 (0.24 to 0.26) |
| Bulgaria | 18217 (8772 to 36788) | 1109.84 (535.65 to 2242.75) | 24058 (11710 to 48755) | 1226.52 (596.9 to 2482.82) | 0.35 (0.33 to 0.36) |
| Burkina Faso | 4024 (1961 to 8103) | 813.34 (396.34 to 1638.18) | 9170 (4460 to 18414) | 900.28 (438.25 to 1809.94) | 0.33 (0.33 to 0.34) |
| Burundi | 2197 (1071 to 4431) | 842.17 (411.41 to 1698.54) | 4445 (2169 to 8853) | 867.37 (423.15 to 1733.76) | 0.12 (0.11 to 0.14) |
| Cabo Verde | 268 (129 to 538) | 903.28 (433.93 to 1812.63) | 572 (279 to 1159) | 1093.56 (532.27 to 2219.72) | 0.66 (0.65 to 0.67) |
| Cambodia | 3365 (1619 to 6784) | 690.23 (332.89 to 1388.83) | 11531 (5477 to 23447) | 815.11 (388.66 to 1655.47) | 0.6 (0.57 to 0.62) |
| Cameroon | 4530 (2208 to 9067) | 930.8 (453.41 to 1866.93) | 14104 (6881 to 28850) | 1062.34 (518.01 to 2173.65) | 0.4 (0.37 to 0.43) |
| Canada | 42451 (20814 to 86425) | 999.93 (490.35 to 2034.79) | 105222 (51576 to 212668) | 1084.56 (531.51 to 2191.03) | 0.14 (0.07 to 0.2) |
| Central African Republic | 1010 (493 to 2051) | 876.69 (427.95 to 1781.42) | 1904 (929 to 3854) | 916.41 (448.25 to 1849.43) | 0.13 (0.12 to 0.14) |
| Chad | 2702 (1304 to 5413) | 818.28 (395.76 to 1638.86) | 5287 (2577 to 10627) | 864.84 (422.1 to 1739.75) | 0.17 (0.15 to 0.18) |
| Chile | 14689 (7160 to 29688) | 1209.36 (589.71 to 2443.72) | 44951 (21954 to 90947) | 1354.99 (661.74 to 2741.61) | 0.35 (0.31 to 0.38) |
| China | 933169 (446146 to 1883344) | 965.06 (462.89 to 1947.32) | 3007414 (1438250 to 6086673) | 1127.77 (540.49 to 2280.65) | 0.63 (0.54 to 0.72) |
| Colombia | 21861 (10620 to 44343) | 1097.95 (533.69 to 2227.74) | 86785 (42017 to 177516) | 1248.3 (604.05 to 2553.76) | 0.45 (0.44 to 0.46) |
| Comoros | 187 (91 to 377) | 862.26 (422.2 to 1742.85) | 534 (259 to 1082) | 978.26 (474.39 to 1981.77) | 0.45 (0.43 to 0.48) |
| Congo | 1211 (587 to 2467) | 1027.95 (498.81 to 2094.1) | 3049 (1472 to 6155) | 1130.36 (546.87 to 2286.65) | 0.3 (0.28 to 0.32) |
| Cook Islands | 16 (8 to 32) | 1104.47 (538.78 to 2204.57) | 43 (21 to 86) | 1258.18 (610.57 to 2547) | 0.4 (0.37 to 0.44) |
| Costa Rica | 2319 (1123 to 4697) | 1116.89 (540.99 to 2263.03) | 8716 (4237 to 17592) | 1252.24 (608.42 to 2529.83) | 0.37 (0.36 to 0.39) |
| Coted'Ivoire | 3502 (1692 to 7046) | 884.81 (428.28 to 1785.06) | 11529 (5629 to 23253) | 1012.49 (494.57 to 2041.86) | 0.39 (0.38 to 0.41) |
| Croatia | 8504 (4134 to 17299) | 1111.82 (540.62 to 2263.02) | 14533 (7059 to 29628) | 1198.75 (582.2 to 2439.47) | 0.3 (0.28 to 0.32) |
| Cuba | 14416 (6993 to 29314) | 1118.65 (543.06 to 2271.92) | 30367 (14769 to 62023) | 1236.23 (601.2 to 2523.27) | 0.36 (0.34 to 0.37) |
| Cyprus | 1200 (582 to 2433) | 1118.51 (543.75 to 2263.25) | 3388 (1660 to 6845) | 1232.71 (604.86 to 2488.56) | 0.32 (0.29 to 0.35) |
| Czechia | 21256 (10373 to 42911) | 1163.06 (567.7 to 2346.79) | 35675 (17494 to 72699) | 1239.78 (608.73 to 2521.09) | 0.19 (0.17 to 0.21) |
| Democratic People's Republic of Korea | 16656 (7938 to 33394) | 949.32 (452.51 to 1905.09) | 40646 (19451 to 82395) | 1033.16 (495.12 to 2093.58) | 0.28 (0.26 to 0.29) |
| Democratic Republic of the Congo | 15417 (7443 to 31108) | 919.89 (444.43 to 1859.4) | 35637 (16995 to 72252) | 959.8 (459.04 to 1947.17) | 0.07 (0 to 0.14) |
| Denmark | 13981 (6873 to 28506) | 1301.61 (640.67 to 2650.31) | 19652 (9595 to 40077) | 1250.23 (611.13 to 2545.81) | -0.05 (-0.09 to -0.01) |
| Djibouti | 112 (54 to 224) | 871.92 (421.64 to 1749.67) | 664 (322 to 1343) | 1045.38 (507.67 to 2120.13) | 0.69 (0.64 to 0.74) |
| Dominica | 88 (43 to 179) | 1132.79 (550.37 to 2299.21) | 131 (64 to 266) | 1237.65 (601.43 to 2507.4) | 0.28 (0.23 to 0.32) |
| Dominican Republic | 4829 (2349 to 9853) | 1131.35 (550.4 to 2309.39) | 15199 (7364 to 30546) | 1258.94 (609.96 to 2530.69) | 0.37 (0.36 to 0.38) |
| Ecuador | 7151 (3487 to 14386) | 1173.27 (572.27 to 2360.98) | 26220 (12724 to 53336) | 1299.37 (630.83 to 2642.67) | 0.36 (0.34 to 0.37) |
| Egypt | 25825 (12444 to 52306) | 897.8 (433.79 to 1818.79) | 72114 (35255 to 147256) | 1024.31 (500.07 to 2087.83) | 0.33 (0.29 to 0.37) |
| El Salvador | 3849 (1867 to 7734) | 1092.99 (530.17 to 2196.7) | 9614 (4663 to 19409) | 1242.35 (602.35 to 2508.1) | 0.43 (0.41 to 0.46) |
| Equatorial Guinea | 172 (84 to 344) | 838.58 (408.61 to 1678.24) | 593 (290 to 1196) | 1162.42 (569.15 to 2343.64) | 1.27 (1.19 to 1.34) |
| Eritrea | 797 (382 to 1601) | 815.91 (390.81 to 1642.55) | 2443 (1180 to 4875) | 911.11 (440.9 to 1818.7) | 0.37 (0.36 to 0.38) |
| Estonia | 3306 (1611 to 6655) | 1247.23 (607.95 to 2509.86) | 5052 (2470 to 10214) | 1393.15 (681.18 to 2811.13) | 0.39 (0.35 to 0.42) |
| Eswatini | 305 (147 to 614) | 1014.92 (488.53 to 2043.02) | 707 (347 to 1434) | 1170.58 (574.4 to 2372.33) | 0.41 (0.37 to 0.46) |
| Ethiopia | 18294 (8915 to 36776) | 878.27 (429.31 to 1764.32) | 52239 (25472 to 105259) | 1111.58 (542.15 to 2239.88) | 0.89 (0.85 to 0.93) |
| Fiji | 362 (173 to 730) | 1010.95 (485.54 to 2036.81) | 1050 (513 to 2113) | 1182.79 (578.44 to 2382.34) | 0.5 (0.47 to 0.54) |
| Finland | 11314 (5553 to 23041) | 1199.54 (588.95 to 2441.52) | 21537 (10523 to 43506) | 1267.43 (619.32 to 2556.45) | 0.17 (0.16 to 0.18) |
| France | 131071 (64522 to 265195) | 1193.99 (587.17 to 2415.22) | 231628 (113796 to 468921) | 1264.05 (619.95 to 2557.94) | 0.17 (0.14 to 0.21) |
| Gabon | 664 (321 to 1347) | 976.14 (472.21 to 1981.29) | 1322 (644 to 2686) | 1148.36 (559.76 to 2337.86) | 0.5 (0.47 to 0.53) |
| Gambia | 339 (166 to 687) | 903.17 (441.71 to 1825.96) | 1131 (554 to 2292) | 1063.53 (520.86 to 2154.82) | 0.53 (0.52 to 0.54) |
| Georgia | 8671 (4194 to 17482) | 1099.83 (532.97 to 2221.33) | 9609 (4673 to 19240) | 1179.2 (573.16 to 2362.43) | 0.18 (0.11 to 0.26) |
| Germany | 203891 (100130 to 410809) | 1233.51 (605.56 to 2481.14) | 322849 (157049 to 650728) | 1276.87 (621.04 to 2574.49) | 0.06 (0.04 to 0.08) |
| Ghana | 7188 (3486 to 14635) | 1072.97 (520.46 to 2183.54) | 20550 (9966 to 41449) | 1130.21 (548.05 to 2284.27) | 0.22 (0.11 to 0.34) |
| Greece | 22474 (10883 to 45413) | 1121.54 (543.55 to 2264.62) | 38074 (18530 to 77514) | 1230.78 (598.56 to 2505) | 0.67 (0.52 to 0.81) |
| Greenland | 34 (17 to 69) | 1000.66 (488.13 to 2037.06) | 93 (45 to 187) | 1095.26 (531.72 to 2213.42) | 0.29 (0.26 to 0.32) |
| Grenada | 108 (52 to 218) | 1118.2 (539.35 to 2260.8) | 169 (82 to 342) | 1241.98 (600.96 to 2516.57) | 0.34 (0.29 to 0.38) |
| Guam | 96 (46 to 194) | 1131.82 (544.6 to 2298.88) | 348 (169 to 705) | 1264.04 (611.69 to 2563.85) | 0.37 (0.34 to 0.39) |
| Guatemala | 3856 (1868 to 7768) | 1004.19 (486.49 to 2025.78) | 14837 (7231 to 30167) | 1120.96 (546.65 to 2278.4) | 0.35 (0.34 to 0.36) |
| Guinea | 3338 (1612 to 6757) | 836.7 (404.72 to 1694.01) | 5762 (2789 to 11546) | 915.62 (443.42 to 1837.09) | 0.27 (0.26 to 0.28) |
| Guinea-Bissau | 367 (178 to 738) | 854.81 (415.51 to 1717.39) | 686 (331 to 1386) | 948.03 (458 to 1917.13) | 0.31 (0.3 to 0.32) |
| Guyana | 465 (227 to 942) | 1080.57 (527.21 to 2192.13) | 922 (446 to 1871) | 1215.96 (587.95 to 2469.36) | 0.39 (0.36 to 0.41) |
| Haiti | 3316 (1611 to 6651) | 927.37 (450.21 to 1862.57) | 7855 (3799 to 15839) | 1015.69 (491.47 to 2048.26) | 0.33 (0.32 to 0.34) |
| Honduras | 2395 (1163 to 4804) | 1036.48 (503.38 to 2078.11) | 8482 (4144 to 17031) | 1145.18 (560.04 to 2299.08) | 0.33 (0.33 to 0.34) |
| Hungary | 22551 (11007 to 45670) | 1156.18 (565.11 to 2340.85) | 33540 (16378 to 67643) | 1260.88 (615.47 to 2539.05) | 0.26 (0.22 to 0.3) |
| Iceland | 487 (238 to 988) | 1306.3 (637.59 to 2651.08) | 1011 (497 to 2046) | 1321.79 (650.12 to 2672.89) | 0 (-0.07 to 0.06) |
| India | 439151 (214269 to 884147) | 889.82 (434.4 to 1791.93) | 1509286 (734304 to 3041514) | 1062.72 (517.62 to 2141.27) | 0.56 (0.52 to 0.61) |
| Indonesia | 83711 (40621 to 169549) | 803.36 (390.33 to 1625.45) | 259540 (125918 to 523584) | 963.06 (467.74 to 1943.99) | 0.59 (0.57 to 0.61) |
| Iran (Islamic Republic of) | 27143 (13259 to 54848) | 932.79 (455.4 to 1886.54) | 96083 (46859 to 194247) | 1066.62 (519.86 to 2158.24) | 0.46 (0.38 to 0.54) |
| Iraq | 8577 (4175 to 17491) | 957.24 (465.74 to 1953.26) | 26216 (12836 to 52828) | 1027.66 (502.88 to 2072.33) | 0.19 (0.17 to 0.21) |
| Ireland | 6433 (3129 to 12954) | 1182.48 (575.73 to 2377.59) | 13070 (6398 to 26472) | 1269.3 (621.97 to 2569.93) | 0.21 (0.19 to 0.23) |
| Israel | 7606 (3689 to 15357) | 1186.52 (575.91 to 2391.17) | 20508 (10009 to 41486) | 1272.16 (621.05 to 2570.7) | -0.34 (-0.57 to -0.12) |
| Italy | 145949 (71730 to 295074) | 1228.67 (603.88 to 2482) | 243737 (119387 to 495118) | 1300.71 (637.12 to 2641.81) | 0.29 (0.21 to 0.37) |
| Jamaica | 2627 (1265 to 5346) | 1130.89 (545.11 to 2297.87) | 4823 (2348 to 9765) | 1235.22 (600.79 to 2502.01) | 0.3 (0.28 to 0.32) |
| Japan | 321235 (155888 to 652263) | 1483.71 (720.57 to 3011.42) | 743152 (359704 to 1511581) | 1577.35 (762.74 to 3204.92) | 0.5 (0.26 to 0.74) |
| Jordan | 1325 (643 to 2666) | 981.05 (475.79 to 1977.78) | 8770 (4246 to 17688) | 1110.17 (537.5 to 2239.94) | 0.41 (0.4 to 0.43) |
| Kazakhstan | 17575 (8491 to 35523) | 1179.26 (569.69 to 2386.78) | 29825 (14530 to 60116) | 1393.84 (680.37 to 2811.94) | 0.59 (0.54 to 0.64) |
| Kenya | 8800 (4280 to 17757) | 966.95 (470.93 to 1951.44) | 28712 (13941 to 58235) | 1153.21 (560.45 to 2340.03) | 0.61 (0.58 to 0.63) |
| Kiribati | 42 (20 to 84) | 1043.7 (503.65 to 2116.41) | 89 (43 to 181) | 1143.53 (551.41 to 2317.03) | 0.23 (0.19 to 0.28) |
| Kuwait | 584 (283 to 1193) | 1047.46 (506.73 to 2140.26) | 3135 (1521 to 6290) | 1137.32 (551.74 to 2289.64) | 0.33 (0.31 to 0.35) |
| Kyrgyzstan | 3973 (1914 to 7981) | 1108.01 (535.19 to 2230.18) | 6700 (3221 to 13482) | 1235.55 (594.74 to 2487.71) | 0.45 (0.37 to 0.54) |
| Lao People's Democratic Republic | 1631 (780 to 3258) | 712.93 (342.01 to 1425.42) | 4209 (2022 to 8520) | 834.96 (402 to 1688.13) | 0.56 (0.53 to 0.59) |
| Latvia | 5633 (2716 to 11438) | 1213.37 (585.48 to 2464.51) | 7321 (3584 to 14919) | 1363.65 (668.24 to 2776.02) | 0.44 (0.4 to 0.47) |
| Lebanon | 2378 (1151 to 4813) | 931.73 (450.91 to 1885.78) | 8169 (3970 to 16497) | 1094.57 (532.24 to 2209.56) | 0.52 (0.48 to 0.56) |
| Lesotho | 918 (449 to 1846) | 930.78 (455.63 to 1872.33) | 1366 (669 to 2787) | 1103.76 (540.96 to 2248.63) | 0.61 (0.57 to 0.64) |
| Liberia | 1221 (592 to 2445) | 888.99 (431.87 to 1780.11) | 2136 (1045 to 4317) | 1019.1 (498.4 to 2059.38) | 0.55 (0.5 to 0.6) |
| Libya | 2095 (1022 to 4240) | 989.74 (482.73 to 2004.2) | 5850 (2853 to 11849) | 1085.51 (529.52 to 2198.61) | 0.3 (0.27 to 0.33) |
| Lithuania | 6890 (3358 to 14036) | 1189.73 (580.09 to 2424.74) | 10418 (5066 to 21411) | 1346.19 (654.91 to 2766.82) | 0.46 (0.43 to 0.49) |
| Luxembourg | 875 (426 to 1787) | 1221.64 (595.53 to 2493.49) | 1713 (845 to 3441) | 1274.63 (628.65 to 2562.4) | 0.12 (0.1 to 0.14) |
| Madagascar | 4524 (2185 to 9150) | 803.16 (388.69 to 1621.94) | 9540 (4594 to 19316) | 856.19 (413.27 to 1741.34) | 0.23 (0.22 to 0.24) |
| Malawi | 3572 (1737 to 7209) | 839.66 (409.16 to 1695.83) | 7514 (3647 to 15211) | 944.56 (458.88 to 1912.59) | 0.43 (0.42 to 0.45) |
| Malaysia | 8959 (4313 to 18122) | 868.17 (418.37 to 1757.13) | 35126 (16860 to 71284) | 1016.87 (488.53 to 2062.07) | 0.52 (0.5 to 0.54) |
| Maldives | 78 (38 to 157) | 831.18 (403.07 to 1679.85) | 354 (172 to 713) | 1012.83 (490.66 to 2042.26) | 0.65 (0.63 to 0.68) |
| Mali | 3640 (1747 to 7286) | 829.44 (399.27 to 1661.74) | 8894 (4294 to 17805) | 927.89 (448.87 to 1859.97) | 0.38 (0.37 to 0.39) |
| Malta | 656 (320 to 1322) | 1200.09 (586.29 to 2418.85) | 1712 (837 to 3444) | 1279.52 (625.97 to 2573.06) | 0.18 (0.14 to 0.22) |
| Marshall Islands | 18 (9 to 37) | 1027.06 (494.43 to 2082.18) | 42 (20 to 86) | 1134.52 (543.63 to 2313.19) | 0.29 (0.26 to 0.31) |
| Mauritania | 1068 (517 to 2166) | 932.52 (452.68 to 1891.44) | 2677 (1298 to 5439) | 1072.85 (520.39 to 2181.93) | 0.42 (0.4 to 0.44) |
| Mauritius | 786 (382 to 1605) | 914.23 (444.35 to 1866.02) | 2508 (1225 to 5051) | 1042.53 (509 to 2102.81) | 0.44 (0.43 to 0.46) |
| Mexico | 57716 (28218 to 116686) | 1189.01 (581.72 to 2404.27) | 210355 (102870 to 424988) | 1370.74 (670.25 to 2770.79) | 0.51 (0.48 to 0.53) |
| Micronesia (Federated States of) | 59 (29 to 119) | 1020.23 (495.22 to 2058.4) | 97 (47 to 195) | 1164.2 (567.6 to 2332.96) | 0.41 (0.36 to 0.46) |
| Monaco | 121 (59 to 245) | 1264.88 (618.47 to 2569.63) | 170 (82 to 344) | 1315.14 (636.67 to 2669.31) | 0.12 (0.11 to 0.13) |
| Mongolia | 1215 (584 to 2447) | 1024.39 (492.92 to 2063.31) | 3133 (1505 to 6306) | 1317.32 (634.12 to 2654.48) | 0.88 (0.83 to 0.93) |
| Montenegro | 868 (421 to 1756) | 1144.65 (555.25 to 2316.33) | 1597 (775 to 3267) | 1214.31 (589.69 to 2483.26) | 0.25 (0.23 to 0.27) |
| Morocco | 15377 (7498 to 31473) | 921.81 (449.47 to 1886.38) | 41296 (20157 to 83432) | 1001.89 (488.91 to 2025.85) | 0.23 (0.19 to 0.26) |
| Mozambique | 5224 (2517 to 10569) | 811.12 (391.7 to 1641.41) | 10577 (5130 to 21324) | 911.91 (443.26 to 1836.13) | 0.41 (0.39 to 0.43) |
| Myanmar | 18972 (9179 to 37917) | 726.83 (353.07 to 1450.57) | 50889 (24676 to 102033) | 889.82 (432.24 to 1783.16) | 0.75 (0.71 to 0.79) |
| Namibia | 701 (343 to 1428) | 940 (459.47 to 1912.82) | 1634 (790 to 3310) | 1079.94 (522.96 to 2187.44) | 0.41 (0.39 to 0.44) |
| Nauru | 5 (2 to 10) | 1038.48 (503.7 to 2094.16) | 7 (4 to 15) | 1195.3 (577.07 to 2396.32) | 0.44 (0.41 to 0.47) |
| Nepal | 7657 (3637 to 15364) | 763.08 (364.35 to 1535.94) | 24857 (12012 to 49846) | 893.49 (431.84 to 1792.4) | 0.53 (0.5 to 0.56) |
| Netherlands | 33307 (16279 to 66029) | 1274.44 (622.34 to 2526.52) | 60565 (29504 to 123717) | 1293.57 (630.22 to 2642.46) | -0.06 (-0.16 to 0.04) |
| New Zealand | 6691 (3273 to 13527) | 1291.16 (632.14 to 2607.15) | 15839 (7703 to 32272) | 1418.97 (690.29 to 2890.2) | 0.29 (0.27 to 0.32) |
| Nicaragua | 1757 (852 to 3521) | 1023.33 (496.72 to 2053.06) | 6691 (3274 to 13558) | 1169.26 (572.08 to 2371.29) | 0.43 (0.41 to 0.45) |
| Niger | 2419 (1162 to 4897) | 824.02 (397.16 to 1669.15) | 7827 (3809 to 15570) | 874.97 (426.24 to 1742.83) | 0.21 (0.2 to 0.22) |
| Nigeria | 48569 (23702 to 97642) | 967.78 (472.68 to 1945.9) | 103899 (50599 to 209814) | 1084.81 (528.77 to 2194.06) | 0.41 (0.39 to 0.44) |
| Niue | 3 (2 to 6) | 1104.05 (534.15 to 2237.25) | 3 (2 to 7) | 1243.24 (609.17 to 2508.09) | 0.39 (0.36 to 0.43) |
| North Macedonia | 2296 (1120 to 4689) | 1026.28 (500.72 to 2097.54) | 4955 (2410 to 10093) | 1139.9 (555.09 to 2321.86) | 0.4 (0.38 to 0.42) |
| Northern Mariana Islands | 16 (8 to 32) | 1139.04 (554.9 to 2304.97) | 72 (35 to 145) | 1215.86 (590.73 to 2458.01) | 0.18 (0.14 to 0.22) |
| Norway | 11461 (5596 to 23239) | 1241.61 (606.34 to 2512.17) | 17258 (8455 to 34946) | 1316.58 (645.29 to 2663.58) | 0.29 (0.16 to 0.41) |
| Oman | 641 (310 to 1302) | 933.08 (452.81 to 1895.82) | 2112 (1036 to 4271) | 1118.82 (548.64 to 2264.91) | 0.6 (0.59 to 0.62) |
| Pakistan | 52793 (25494 to 106661) | 818.17 (395.72 to 1651.55) | 126722 (61499 to 254794) | 977.15 (474.51 to 1964.74) | 0.61 (0.59 to 0.62) |
| Palau | 13 (6 to 26) | 1102.79 (533.58 to 2251.45) | 33 (16 to 66) | 1222.4 (592.3 to 2480.65) | 0.3 (0.26 to 0.34) |
| Palestine | 928 (452 to 1883) | 914.87 (445.64 to 1857.82) | 2823 (1375 to 5776) | 1043.4 (508.09 to 2134.99) | 0.41 (0.39 to 0.43) |
| Panama | 1869 (896 to 3809) | 1061.32 (508.96 to 2161.18) | 6706 (3249 to 13573) | 1222.11 (591.97 to 2473.58) | 0.43 (0.41 to 0.44) |
| Papua New Guinea | 1567 (761 to 3189) | 826.09 (400.84 to 1680.61) | 4596 (2218 to 9235) | 914.42 (442.12 to 1835.82) | 0.3 (0.29 to 0.32) |
| Paraguay | 3022 (1473 to 6121) | 1156.18 (563.7 to 2343.3) | 8499 (4104 to 17204) | 1209.6 (584.27 to 2450.83) | 0.14 (0.08 to 0.2) |
| Peru | 15789 (7649 to 32349) | 1152.27 (558.26 to 2362.06) | 52205 (25357 to 105776) | 1284.8 (623.86 to 2604.12) | 0.33 (0.3 to 0.36) |
| Philippines | 26108 (12684 to 52722) | 820.22 (399.17 to 1654.28) | 89985 (43528 to 182061) | 964.27 (466.84 to 1951.75) | 0.48 (0.44 to 0.52) |
| Poland | 63416 (30973 to 128334) | 1126.71 (550.37 to 2281.02) | 127234 (62088 to 258152) | 1291.4 (629.93 to 2619.96) | 0.48 (0.45 to 0.5) |
| Portugal | 21436 (10444 to 43110) | 1150.07 (560.74 to 2311.57) | 40569 (19680 to 82026) | 1254.07 (608.47 to 2533.29) | 0.42 (0.31 to 0.54) |
| Puerto Rico | 6014 (2928 to 12178) | 1291.09 (629.08 to 2612.53) | 13427 (6544 to 27383) | 1392.19 (678.95 to 2835.42) | 0.27 (0.26 to 0.29) |
| Qatar | 84 (41 to 172) | 1046.94 (512.32 to 2132.68) | 830 (404 to 1664) | 1140.88 (557.49 to 2298.39) | 0.25 (0.23 to 0.27) |
| Republic of Korea | 50964 (25065 to 101937) | 1575.24 (775.62 to 3150.82) | 203393 (98675 to 412471) | 1635.19 (793.2 to 3316.89) | 0.23 (0.1 to 0.35) |
| Republic of Moldova | 5754 (2802 to 11727) | 1060.42 (516.84 to 2165.03) | 9782 (4748 to 19744) | 1221.91 (592.77 to 2469.56) | 0.58 (0.51 to 0.66) |
| Romania | 36420 (17761 to 74462) | 1030.24 (502.35 to 2106.62) | 58072 (28239 to 118199) | 1143.24 (555.76 to 2325.01) | 0.39 (0.37 to 0.41) |
| Russian Federation | 314487 (152578 to 641043) | 1368.98 (664.97 to 2792.54) | 464132 (225538 to 941729) | 1420.01 (689.74 to 2883.27) | 0.27 (0.22 to 0.32) |
| Rwanda | 2526 (1222 to 5081) | 816.16 (394.95 to 1642.07) | 6280 (3067 to 12676) | 901.79 (440.26 to 1820.34) | 0.37 (0.35 to 0.4) |
| Saint Kitts and Nevis | 64 (31 to 129) | 1203.52 (588.17 to 2427.83) | 112 (55 to 226) | 1313.24 (638.16 to 2661.7) | 0.28 (0.26 to 0.29) |
| Saint Lucia | 124 (61 to 252) | 1101.55 (540.95 to 2236.68) | 373 (182 to 761) | 1246.27 (607.11 to 2541.44) | 0.36 (0.32 to 0.4) |
| Saint Vincent and the Grenadines | 102 (49 to 207) | 1107.26 (534.33 to 2237.67) | 223 (109 to 456) | 1217.77 (595.98 to 2487.39) | 0.33 (0.31 to 0.35) |
| Samoa | 103 (49 to 207) | 1040.3 (501.48 to 2093) | 195 (95 to 395) | 1167.98 (570.5 to 2367.69) | 0.4 (0.38 to 0.41) |
| San Marino | 57 (28 to 116) | 1225.78 (595.47 to 2489.46) | 122 (59 to 246) | 1283.58 (626.12 to 2603.89) | 0.13 (0.11 to 0.15) |
| Sao Tome and Principe | 77 (37 to 155) | 965.19 (467.93 to 1963.12) | 139 (67 to 282) | 1162.31 (563.78 to 2354.74) | 0.64 (0.62 to 0.66) |
| Saudi Arabia | 5649 (2722 to 11372) | 938.34 (453.19 to 1889.9) | 18900 (9303 to 38371) | 1111.42 (546.32 to 2250.59) | 0.28 (0.16 to 0.39) |
| Senegal | 3318 (1600 to 6667) | 903.03 (436.15 to 1814.06) | 8819 (4260 to 17754) | 1008.67 (487.52 to 2032.66) | 0.32 (0.31 to 0.34) |
| Serbia | 14642 (7069 to 29563) | 1034.44 (499.94 to 2098.6) | 26112 (12668 to 52847) | 1155.69 (560.68 to 2338.38) | 0.42 (0.4 to 0.44) |
| Seychelles | 66 (32 to 134) | 915.68 (442.79 to 1846.3) | 147 (72 to 296) | 1052.93 (513.46 to 2128.28) | 0.44 (0.39 to 0.49) |
| Sierra Leone | 2032 (987 to 4053) | 844.45 (410.67 to 1682.86) | 3962 (1913 to 7982) | 967.84 (468.11 to 1948.71) | 0.45 (0.41 to 0.48) |
| Singapore | 3900 (1884 to 7848) | 1561.83 (754.24 to 3145.87) | 17889 (8704 to 36275) | 1620.66 (788.54 to 3288.94) | 0.12 (0.1 to 0.14) |
| Slovakia | 8950 (4341 to 18104) | 1147.96 (556.94 to 2320.93) | 16100 (7846 to 32763) | 1251.61 (609.91 to 2546.47) | 0.27 (0.24 to 0.29) |
| Slovenia | 3607 (1758 to 7293) | 1151.05 (561.65 to 2327.54) | 7278 (3539 to 14801) | 1228.51 (596.41 to 2497.44) | 0.19 (0.17 to 0.22) |
| Solomon Islands | 129 (62 to 263) | 900.28 (436.2 to 1834.27) | 366 (177 to 738) | 1046.24 (506.16 to 2109.38) | 0.47 (0.45 to 0.49) |
| Somalia | 1762 (852 to 3553) | 833.64 (403.35 to 1686.16) | 5292 (2545 to 10645) | 873.25 (420.6 to 1756.97) | 0.19 (0.18 to 0.2) |
| South Africa | 28932 (14115 to 58679) | 1211.46 (591.08 to 2458.43) | 71021 (34758 to 143059) | 1301.23 (637.07 to 2624.01) | 0.27 (0.25 to 0.29) |
| South Sudan | 2374 (1152 to 4742) | 798.3 (388.35 to 1592.16) | 3265 (1591 to 6535) | 855.54 (417.3 to 1711.74) | 0.27 (0.25 to 0.28) |
| Spain | 87056 (42398 to 177530) | 1188.51 (579.1 to 2421.86) | 159850 (78221 to 322937) | 1279.06 (625.87 to 2579.5) | 0.19 (0.14 to 0.23) |
| Sri Lanka | 9515 (4572 to 19113) | 784.45 (377.96 to 1575.7) | 32452 (15733 to 66071) | 923.1 (448.1 to 1876.62) | 0.55 (0.53 to 0.56) |
| Sudan | 8112 (3893 to 16419) | 769.32 (370.57 to 1555.4) | 19502 (9361 to 39347) | 944.21 (453.93 to 1905.09) | 0.7 (0.65 to 0.74) |
| Suriname | 354 (171 to 713) | 1197.73 (576.37 to 2415.92) | 996 (485 to 2007) | 1292.01 (629.04 to 2604.19) | 0.29 (0.27 to 0.3) |
| Sweden | 21238 (10228 to 43971) | 1047.5 (504.51 to 2164.17) | 31918 (15393 to 65024) | 1131.89 (545.4 to 2301.4) | 0.4 (0.17 to 0.62) |
| Switzerland | 16189 (7931 to 32945) | 1196.27 (586.37 to 2430.62) | 28632 (14033 to 58104) | 1231.68 (603.21 to 2498.41) | 0.09 (0.09 to 0.1) |
| Syrian Arab Republic | 5132 (2488 to 10421) | 886.21 (429.35 to 1800.91) | 15924 (7665 to 31764) | 1027.51 (494.58 to 2053.02) | 0.52 (0.49 to 0.54) |
| Taiwan (Province of China) | 20280 (9771 to 41189) | 1043.82 (503.88 to 2118.43) | 69837 (33522 to 141418) | 1228.41 (589.2 to 2488.36) | 0.58 (0.56 to 0.6) |
| Tajikistan | 3046 (1474 to 6130) | 967.26 (468.21 to 1946.94) | 6520 (3164 to 13114) | 1045.95 (507.94 to 2107.72) | 0.28 (0.21 to 0.35) |
| Thailand | 30273 (14739 to 60693) | 779.88 (379.82 to 1564.86) | 136633 (66234 to 275075) | 967.48 (468.9 to 1948.92) | 0.74 (0.73 to 0.75) |
| Timor-Leste | 181 (88 to 365) | 697.67 (338.98 to 1405.45) | 881 (426 to 1790) | 835.84 (404.37 to 1694.2) | 0.71 (0.66 to 0.76) |
| Togo | 1131 (547 to 2275) | 876.02 (423.93 to 1763.55) | 3966 (1928 to 7948) | 1012.91 (492.58 to 2036.14) | 0.47 (0.44 to 0.49) |
| Tokelau | 2 (1 to 4) | 996.38 (480.82 to 2008.54) | 2 (1 to 5) | 1184.67 (580.48 to 2409.52) | 0.59 (0.56 to 0.62) |
| Tonga | 66 (32 to 134) | 1017.59 (489.3 to 2063.56) | 110 (53 to 222) | 1144.17 (551.59 to 2311.62) | 0.33 (0.28 to 0.38) |
| Trinidad and Tobago | 1239 (600 to 2484) | 1200.8 (581.72 to 2406.7) | 3298 (1615 to 6744) | 1291.07 (632.1 to 2639.91) | 0.28 (0.25 to 0.3) |
| Tunisia | 5422 (2638 to 11081) | 916.28 (446.44 to 1870.45) | 17694 (8584 to 35675) | 1062.5 (516.05 to 2145.39) | 0.49 (0.48 to 0.51) |
| Turkey | 35979 (17264 to 72644) | 916.24 (440.09 to 1851.52) | 127490 (62461 to 259367) | 1092.36 (535.11 to 2222.67) | 0.57 (0.48 to 0.66) |
| Turkmenistan | 2301 (1107 to 4604) | 1065.42 (513.64 to 2133.6) | 5757 (2736 to 11452) | 1270.96 (605.81 to 2528.64) | 0.63 (0.56 to 0.7) |
| Tuvalu | 8 (4 to 16) | 999.17 (482.43 to 2030.84) | 15 (7 to 31) | 1167.53 (565.87 to 2392.15) | 0.5 (0.47 to 0.53) |
| Uganda | 5846 (2814 to 11734) | 819.22 (395.5 to 1646) | 14180 (6881 to 28415) | 921.26 (447.8 to 1849.62) | 0.4 (0.39 to 0.41) |
| Ukraine | 114891 (55961 to 233614) | 1223.63 (596.08 to 2489.43) | 141079 (68352 to 282977) | 1333.53 (646.19 to 2674.51) | 0.33 (0.3 to 0.36) |
| United Arab Emirates | 327 (157 to 660) | 988.93 (476.86 to 1995.87) | 3300 (1609 to 6551) | 1054.44 (513.23 to 2108.95) | 0.21 (0.17 to 0.25) |
| United Kingdom | 158262 (77480 to 321892) | 1301.36 (637.93 to 2642.92) | 233205 (114287 to 470669) | 1381.39 (677.59 to 2785.27) | 0.06 (-0.08 to 0.2) |
| United Republic of Tanzania | 11063 (5346 to 22442) | 904.86 (438.02 to 1835.56) | 26649 (12861 to 53794) | 971.52 (469.41 to 1962.34) | 0.12 (0.07 to 0.17) |
| United States of America | 619408 (303714 to 1254598) | 1459.88 (716.01 to 2953.62) | 1207544 (595095 to 2437772) | 1529.34 (753.72 to 3086.66) | -0.04 (-0.2 to 0.12) |
| United States Virgin Islands | 122 (59 to 246) | 1270.56 (618.87 to 2568.4) | 345 (167 to 701) | 1357.7 (658.37 to 2753.6) | 0.22 (0.19 to 0.24) |
| Uruguay | 6440 (3106 to 13018) | 1241.8 (599.23 to 2508.15) | 9846 (4803 to 19702) | 1359.06 (662.83 to 2715.28) | 0.29 (0.28 to 0.31) |
| Uzbekistan | 14610 (7097 to 29549) | 1084.39 (527.42 to 2194.92) | 35921 (17260 to 71646) | 1232.64 (592.36 to 2462.44) | 0.4 (0.35 to 0.45) |
| Vanuatu | 57 (27 to 114) | 854.31 (410.15 to 1716.57) | 180 (88 to 365) | 966.93 (470.06 to 1957.6) | 0.39 (0.39 to 0.4) |
| Venezuela (Bolivarian Republic of) | 12735 (6137 to 25700) | 1153.09 (556.05 to 2327.79) | 47142 (22741 to 94771) | 1253.91 (604.87 to 2522.76) | 0.13 (0.06 to 0.21) |
| Viet Nam | 34842 (16930 to 69830) | 713.74 (347.14 to 1431.4) | 99755 (47976 to 200626) | 847.49 (408.23 to 1706.62) | 0.61 (0.59 to 0.63) |
| Yemen | 3951 (1906 to 8053) | 750.95 (363.3 to 1528.82) | 13434 (6414 to 26973) | 886.87 (424.46 to 1779.88) | 0.63 (0.59 to 0.67) |
| Zambia | 2729 (1311 to 5533) | 913.13 (439.64 to 1852.25) | 6786 (3270 to 13690) | 976.47 (471.86 to 1970.1) | 0.26 (0.2 to 0.32) |
| Zimbabwe | 4177 (2028 to 8493) | 914.86 (444.19 to 1858.1) | 7042 (3447 to 14086) | 948.49 (464.2 to 1897.88) | 0.02 (-0.02 to 0.06) |
